# A Copper Cage‐Complex as Mimic of the pMMO Cu_C_ Site

**DOI:** 10.1002/anie.202206120

**Published:** 2022-07-19

**Authors:** Sarah C. Bete, Leander K. May, Philipp Woite, Michael Roemelt, Matthias Otte

**Affiliations:** ^1^ Institute of Inorganic Chemistry University of Goettingen Tammannstraße 4 37077 Göttingen Germany; ^2^ Institut für Chemie Humboldt-Universität zu Berlin Brook-Taylor-Straße 2 12489 Berlin Germany

**Keywords:** Biomimetic Synthesis, Cage Compounds, Enzyme Models, Oxidation

## Abstract

The active site of particulate methane monooxygenase (pMMO) and its mechanism of action are not known. Recently, the Cu_C_ site emerged as a potential active site, but to date it lacks any study on biomimetic resemblance of the coordination environment provided by the enzyme. Here, the synthesis of a cage ligand providing such an environment is reported. Copper is incorporated, and coordination occurs by the two imidazole and one carboxylate group offered by the ligand. Depending on the oxidation state, it can adopt different coordination modes, as evidenced by the solid‐state structures and computational investigation. The copper(I) state readily reacts with dioxygen and thereby undergoes CH activation. Moreover, the catalytic aerobic oxidation of hydroquinones as ubiquinol mimics is shown. Clean one‐electron oxidation occurs under mild conditions and EPR analysis of the copper(II) state in the presence of water reveals striking similarities to the data obtained from pMMO.

## Graphical abstract

A series of copper complexes that replicate the Cu_C_ site of particulate methane monooxygenase is presented. Although this site is of great interest in the debate about the nature of the active site, it has not yet been modeled in a biomimetic study. The detailed replication leads to striking EPR similarities of the copper(II) state to purified pMMO. Further aerobic oxidation of hydroquinones as bioinspired substrates is shown.

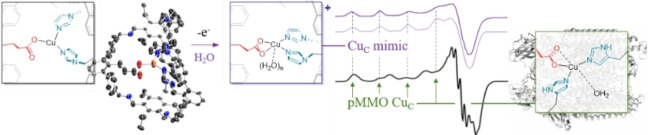

The development of catalysts for selective aerobic methane oxidation to methanol is very desirable, especially from environmental perspectives.[^1^, ^2^] In nature, this challenging transformation proceeds in a very controlled fashion, which evokes interest in the process itself and the potential applicability of the underlying principles.^[3]^ Two types of enzymes are known to catalyze methane hydroxylation, called particulate and soluble methane monooxygenase (pMMO, and sMMO), the latter one being relatively well understood on the one hand,^[4]^ but also the less efficient on the other.^[5]^ Obtaining reliable information on the more potent pMMO is very challenging. Its nature as a membrane‐bound protein, the fact that it has several copper binding sites allocated to the different subunits (PmoA, PmoB and PmoC) and its tendency to lose its metal cofactors upon isolation seem to be the main factors that impede identification of the active site. As there are differing protocols for preparatory treatment, researchers draw contradictory conclusions on the structure and location of the active site, and accordingly also on the operating principle for methane hydroxylation. Even within the last year several opposing reports have been published on that topic.[^6^, ^7^, ^8^]

Copper occupancy of PmoC was shown to be crucial for catalytic activity in 2019 already,^[9]^ and very recently again by cryo‐electron microscopy studies on samples that still showed catalytic activity.^[8]^ The activity goes along with an unusually high preservation of the enzyme structure, which also enables observation of the PmoC region by imaging techniques, and that for the first time in the absence of zinc.^[10]^ Thereby, the already widely accepted constellation of Cu_C_ with coordination of two imidazole and one carboxylate was confirmed (see Figure 1).

**Figure 1:**
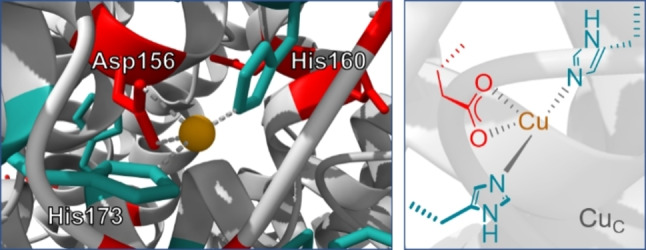
Cu_C_ site in Mc. Sp. Str. Rockwell pMMO.[^8^, ^21^]

The activity dependence on an undamaged PmoC assembly and the conservation among all types of pMMOs as well as the redox behavior of the embedded copper strongly supports the active site actually being located in the PmoC subunit. Functional mimicry on the other hand still points towards involvement of several copper ions, as at least stoichiometric aerobic methane oxidation was observed with (semi)‐synthetic tricopper clusters,^[11]^ structures that are described to form at the PmoA/ PmoC interface.

For Cu_C_ as the active site, a full catalytic cycle is proposed and underlined by computational methods,^[12]^ but the biomimetic contribution is low. This is due to the fact that pMMO biomimicry has long been inspired by the Cu_B_ site, possessing the histidine brace motif, as this was believed to be the active site.^[13]^ There are complexes with NNO(O) coordination to copper,[^14^, ^15^] but to our knowledge none is known to resemble the pMMO Cu_C_ site and notably, there is no copper complex with binding of one carboxylate and two imidazole ligands, as this motif would not self‐assemble without enforcement.

Here we provide a detailed structural and functional mimic of the Cu_C_ site. Thereby we exploit our recently developed approach towards quasi‐heteroleptic cage complexes.^[16]^ This concept allows us to replicate the pseudo‐chelating coordination environment provided by amino acid side chain residues. With this, we have previously reported on the resemblance of related enzymatic binding sites, specifically of the 2‐his‐1‐carboxylate facial triade coordination motif that is found in iron oxygenases.

We now employ cage **1** as a ligand for copper. As the one we used for our studies on iron, **1** is an endohedrally functionalized cage that offers two imidazoles and one carboxylate, it simply misses a methylene group in the carboxylate linker. Apart from a more efficient synthesis, the alteration adds more advantages such as stronger solubility differences that generally ease purification. Scheme 1 depicts the synthesis of **1**.

**Scheme 1:**
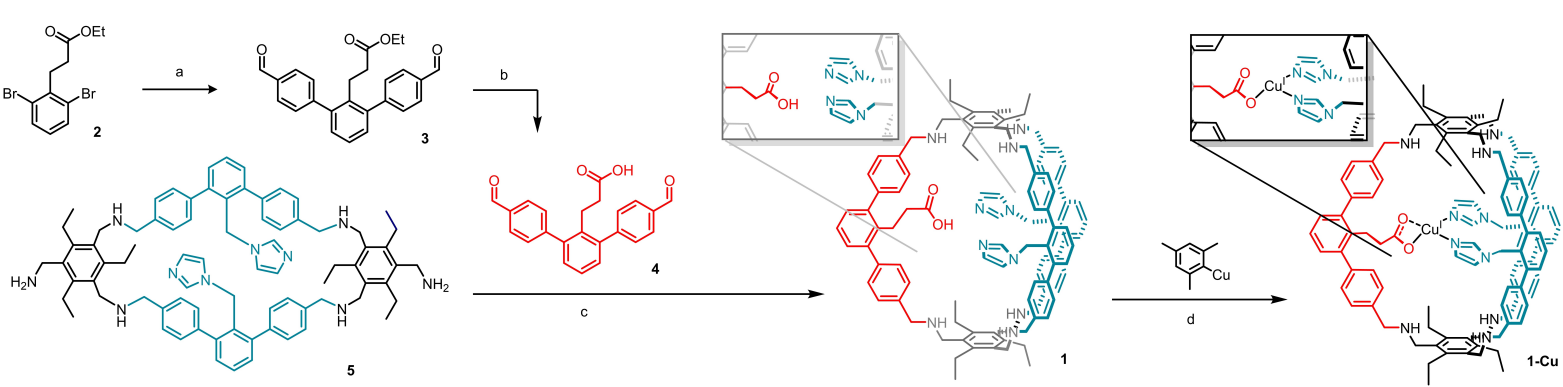
Synthesis of **1** and reaction towards **1‐Cu**. a) 5 mol % Pd(PPh_3_)_4_, 3 equiv 4‐formylphenylboronic acid, Na_2_CO_3_, toluene, EtOH, H_2_O, 100 °C, 72 h, (81 %); b) 5 equiv pTsOH, MeCN, H_2_O, 100 °C, 72 h (95 %), c) DCM, MeOH, rt, 48 h, then NaBH_4_, aq. work‐up (72 %), d) 0.9 equiv mesityl copper, C_6_H_6_, rt, 30 min (86 %).

We started with literature known building block **2** that can undergo a Suzuki coupling to give the dialdehyde and ester‐functionalized building block **3** with 81 % yield. Acid‐catalyzed ester hydrolysis quantitatively yields the corresponding carboxylic acid **4** that was further reacted with macrocycle **5** via reductive amination to give **1** in 72 % yield. **1** is treated with mesityl copper in benzene. Via this protocol we could exclude any other unit to coordinate. A shift of all ^1^H NMR resonances, primarily of the ones assigned to the endohedral functionalities, is observed, already suggesting coordination of both the carboxy group and the imidazole moieties (see Figure 2, imidazole functionality related signals are enlightened in turquoise, carboxyl linker related ones in red). In comparison, deprotonation to alkali‐metal salts does not strongly shift the ethylene group signals. Excess of strong bases, and therefore also excess of mesityl copper leads to unselective further reaction. Surprisingly, but to our delight, excess ligand precipitates from the reaction solution, allowing for a highly facilitated isolation of **1**.

**Figure 2:**
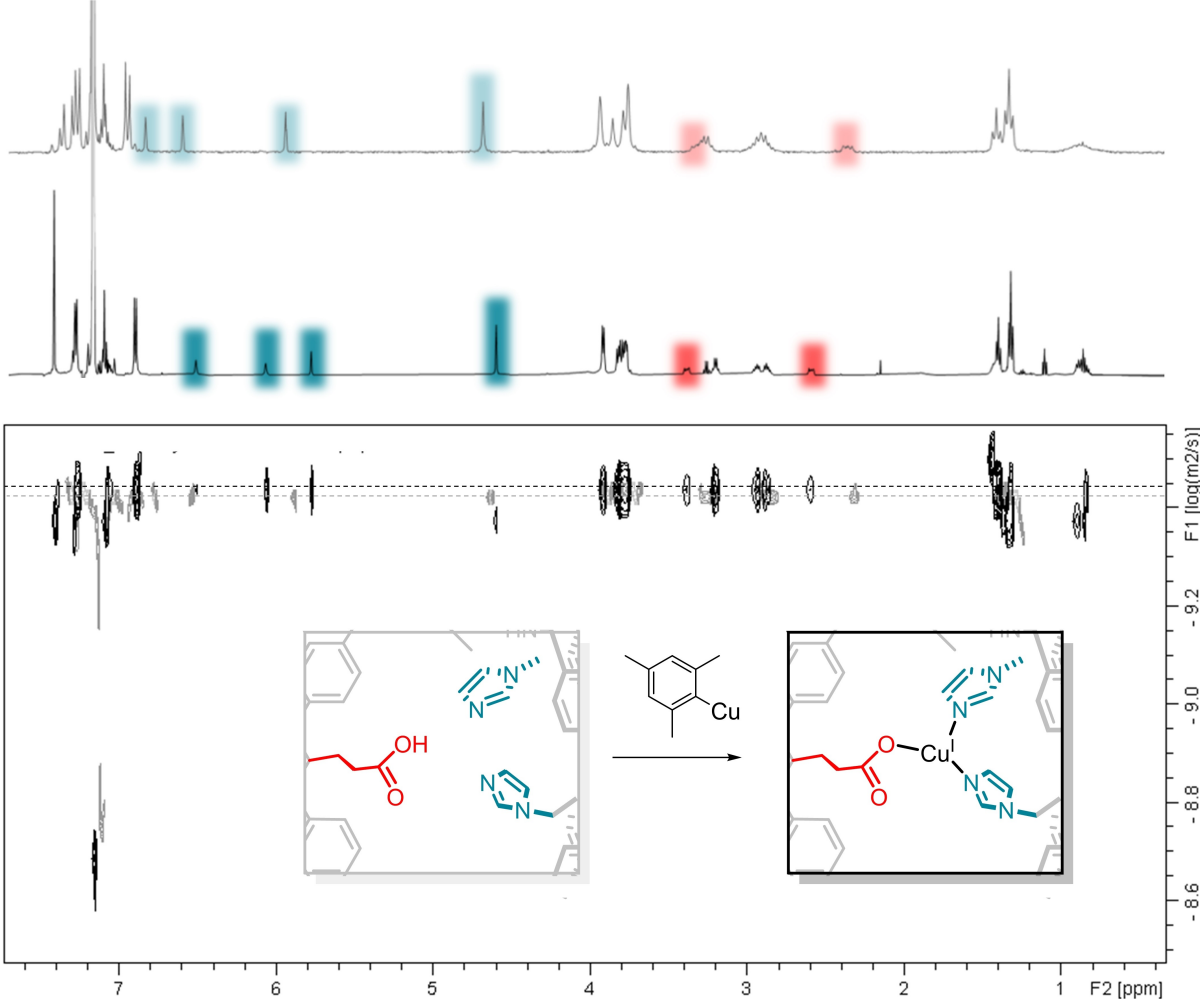
^1^H (top) and ^1^H DOSY NMR spectra (bottom) of **1** (grey) and **1‐Cu** (black) in C_6_D_6_.

High‐resolution ESI MS connects [C_101_H_107_N_10_O_2_Cu]^+^ to the most prominent signal, a complex that would result from oxidation under measurement conditions. IR spectroscopy does not detect the COOH carbonyl stretching vibration of the ligand, confirming complete conversion, and ruling out an equilibrium with the ligand on the NMR timescale. Thus, we can determine a diffusion coefficient for **1‐Cu** by DOSY NMR which gives a value of 3.65×10^−10^ m^2^ s^−1^, and thereby an estimated hydrodynamic radius of 9.6 Å, which is somewhat smaller than the ligand itself with 10.0 Å. This trend could be provoked by a more rigid geometry upon coordination. Size and general shape are preserved and unlike was observed before for the NNO‐chelate to copper interaction,^[15]^ any kind of dimerization can be excluded. This nicely demonstrates a big advantage of our ligand design, as also for the previous system we have observed this particular—and for these kinds of studies very beneficial—difference to complexes with similar coordinating residues.

Chemical oxidation of **1‐Cu** with an excess of ferrocenium salt (Figure 3a) leads to the disappearance of the sharp NMR signals and afterwards only broad resonances, though all of them in the diamagnetic region, are observed. Mass spectrometry gives the same spectrum as before, as for **1‐Cu** we already observed the oxidized compound. Analysis by DOSY NMR shows that there is no change in the diffusion coefficient, accounting for a monomeric species also in the case of copper(II) (See Figures S21 and S28).

**Figure 3:**
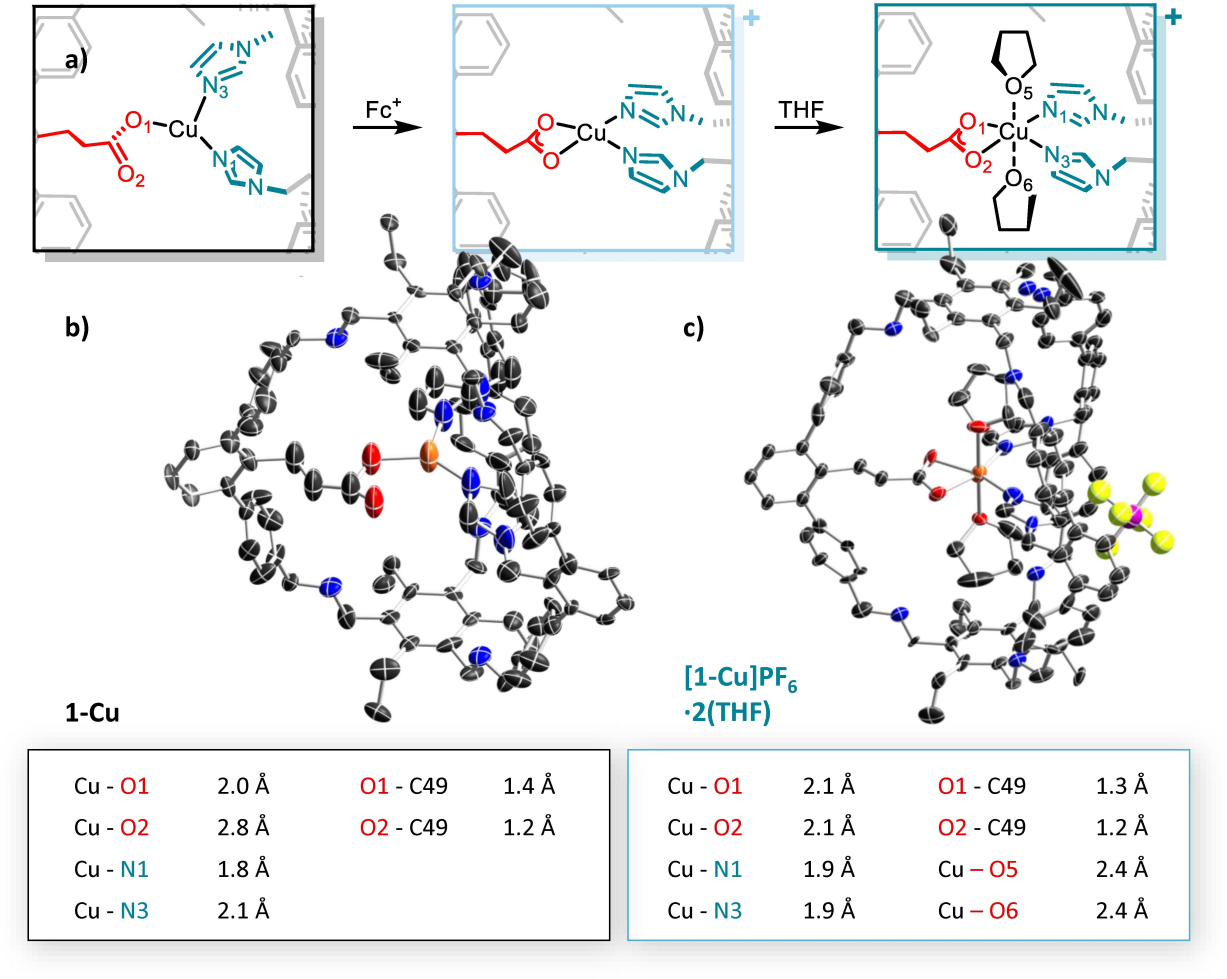
a) Oxidation of **1‐Cu**, proposed structure of [**1‐Cu]PF_6_** and reaction with THF, solid‐state structures of b) **1‐Cu** and c) [**1‐Cu]PF_6_** ⋅**2 (THF)** and corresponding selected bond lengths. Hydrogen atoms are omitted for clarity.

We managed to get solid‐state structures of both **1‐Cu** (Figure 3b) and the THF adduct of the oxidized complex **[1‐Cu]PF_6_** ⋅**2 (THF)** (Figure 3c) at resolutions of 0.95 Å and 1.04 Å, respectively. Unfortunately, the structures are of poor quality and many restraints and constraints had to be applied. The SQUEEZE protocol was employed to remove electron density.^[17]^ Despite the low quality of the crystals, the connectivity of **1‐Cu** and **[1‐Cu]PF_6_** ⋅**2 (THF)** can be assigned. Most importantly, coordination occurs by all three functional groups. **1‐Cu** appears to have monodentate carboxylate coordination, as to conclude from the strong difference in the obtained Cu−O bond lengths (2.0 Å and 2.8 Å). There is no indication for additional ligands, giving a three‐coordinate copper complex as we have reported for the related tris‐imidazole ligand.^[18]^ The complex changes its geometry upon oxidation, and carboxylate coordination gets bidentate in **[1‐Cu]PF_6_** ⋅**2 (THF)** (Cu−O bond lengths 2.1 Å and 2.1 Å). We have observed that unusual binding fashion already for the mentioned iron(II)‐cage complex.^[16]^ Two THF moieties point towards the copper center, resulting in a strongly distorted octahedral geometry, and showing that there is enough space for additional ligands or guest incorporation.

The structural assignments of **1‐Cu** and **[1‐Cu]PF_6_** ⋅**2 (THF)** are confirmed by density functional theory calculations. In the optimized geometry of **1‐Cu** shown in Figure 4, the carboxylate group coordinates the Cu center in an η^1^‐fashion with a Cu−O distance of 2.00 Å which coincides exactly with the experimental value. Also the computed bond lengths of the two imidazole rings that complete the biomimetic trigonal coordination environment agree reasonably well with the crystal structure (2.00 Å and 1.94 Å). In the case of **[1‐Cu]PF_6_** ⋅**2 (THF)** our calculations predict a distorted octahedral coordination of the Cu center (Figure 4; right). Accordingly, the carboxylate acts as a bidentate ligand in the equatorial plane, while two THF molecules symmetrically bind along the vertical axis. As for **1‐Cu** the predicted bond lengths are in good agreement with the crystallographic data (see Tables S5 and S6).

**Figure 4:**
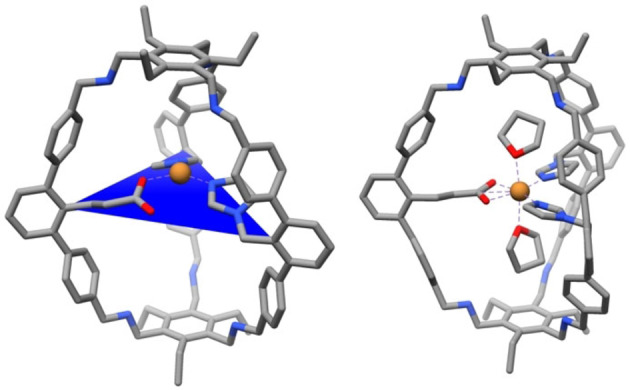
Left: Optimized structure of **1‐Cu** that features a trigonally coordinated Cu center. Right: Optimized structure of **[1‐Cu]PF_6_** ⋅**2 (THF)** that features a distorted octahedral Cu center. Carbon: grey, oxygen: red; nitrogen: blue; hydrogens omitted for clarity.

For pMMO, structural data of metal‐occupied Cu_C_ sites have been reported. For copper, a resolution of 2.16 Å has been obtained and for pMMO with a zinc occupied Cu_C_ site a structure with 2.80 Å resolution has been reported.[^8^, ^19^] For the structures of the enzymes, metal–nitrogen bond lengths within a range between 2.0 and 2.1 Å have been observed. We observe Cu−N bond lengths of 1.8 Å and 2.1 Å in **1‐Cu** and 1.9 Å in **[1‐Cu]PF_6_** ⋅**2 (THF)**. The Cu−O bond lengths in **1‐Cu** and **[1‐Cu]PF_6_** ⋅**2 (THF)** are with 2.0 Å and 2.1 Å shorter compared to the values of 2.4 Å to 2.6 Å reported for the pMMOs structures (see Table S3).

The pocket of the pMMO Cu_C_ site appears to be quite flexible and enables coordination motifs with very different angles. The His‐M‐His bonding angles are 171° for Cu and 95° for Zn while the O−M−His angles lie between 79° and 114°. Also, our cage ligand **1** is able to show a high degree of flexibility enabling different coordination modes highlighting the biomimicking approach we follow to study enzymatic active site pockets (see Table S4).

Having established the structure of **1‐Cu**, we focused on its reactivity. In fact, **1‐Cu** readily reacts with oxygen, but that results in a non‐defined product mixture, and also in the presence of potential substrates such as toluene no oxidation products could be identified. Analysis of the reaction outcome by mass spectrometry led us to the conclusion that instead the cage backbone is oxidized, presumably at the benzamine moieties, as we observe signals that correspond to species that lack the mass of two, four, six, and eight hydrogen atoms. Further proof is obtained by IR, as an additional vibration frequency at 1643 cm^−1^ fits exactly what one expects for a CN stretching vibration frequency of the resulting benzimine function and thus shows the capability of **1‐Cu** to engage in C−H activation. The observed ligand oxidation, in absence and presence of external substrates, hints towards a very reactive copper oxygen species being formed initially. Unfortunately, all our approaches to obtain information on the oxygen activation product from **1‐Cu** via low‐temperature UV/Vis (see Figure S41) or EPR were not successful.

We wondered if selective oxidation could be achieved with functionalized substrates that would preorganize within the cavity. Inspired by pMMO ubiquinol activity and the proposed catalytic cycle on hydroquinone co‐oxidation,^[12]^ we explored the reactivity of **1‐Cu** and **[1‐Cu]PF_6_** in the presence of trimethylhydroquinone (TMHQ). As seen in Table 1, both cage‐complexes catalyze the transformation towards the quinone, **1‐Cu** gives quantitative yields and **[1‐Cu](PF_6_)** a yield of 90 % (entries 1 and 2). Replacing the cage complexes for Cu(MeCN)_4_PF_6_ as an alternate copper source results in no conversion (entry 3). It has very recently been shown that amines, although at higher temperatures, can engage in the oxidation of TMHQ.^[20]^ Indeed, empty cage **1** is also able to catalyze the transformation resulting in 33 % oxidation product (see Figure S37). Knowing that **1** is an active catalyst by itself, the quantitative conversion of TMHQ with **1‐Cu** points towards an interplay of metal center, substrate and amines. This is reminiscent to the H‐bond donating or accepting amino acid residues located in enzymatic active pockets causing substrate preorganization or activation. In addition to TMHQ, we investigated also the oxidation of 2,3‐dimethylhydroquinone (entries 5 and 6), 2‐methylhydroquinone (entries 7 and 8) and 2‐methoxyhydroquinione (entries 9 and 10) as more challenging substrates with **1‐Cu** and **1**. For all substrates investigated **1‐Cu** performs better than **1**. For the three substrates, yields of 71 %, 86 % and 51 % have been obtained with **1‐Cu** (entries 5,7 and 9) while **1** gave only 12 %, 20 % and in the case of 2‐methoxyhydroquinione 0 % yield (entries 6, 8 and 10). The low yields obtained with empty cage **1** is in accordance to other reports employing amines as catalysts for these reactions.^[20]^

**Table 1:** **1‐Cu** and **[1‐Cu]PF_6_** catalyzed aerobic oxidation of hydroquinones.^[a]^

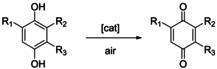					
Entry	[cat]	R_1_	R_2_	R_3_	Yield [%]
1	**1‐Cu**	Me	Me	Me	quant.
2	**[1‐Cu]PF_6_**	Me	Me	Me	90
3	Cu(MeCN)_4_PF_6_	Me	Me	Me	0
4	**1**	Me	Me	Me	33
5	**1‐Cu**	H	Me	Me	71
6	**1**	H	Me	Me	12
7	**1‐Cu**	H	Me	H	86
8	**1**	H	Me	H	20
9	**1‐Cu**	H	OMe	H	51
10	**1**	H	OMe	H	0

[a] 5 mol % [cat], DCM‐D_2_, −20 °C, 20 min.

Insights on the extent of structural resemblance of the natural binding site are provided by EPR analysis. Figure 5 top shows the EPR spectrum of **[1‐Cu]PF_6_** in frozen solution. First of all, we can confirm coordination of both imidazole ligands, as the data can pleasently be fitted with rather strong coupling to two nitrogen atoms, additional to a copper hyperfine splitting of 533 MHz. Very interestingly, after reacting [**1‐Cu]PF_6_** with water, the observed A_II_ coupling tensor decreases to 461 MHz, a value that is very comparable to the one found for the copper center in the PmoC subunit of purified Mc. sp. str. Rockwell pMMO (440 MHz), assigned to Cu_C._ Also the g‐values we obtain after addition of water (2.05, 2.08 and 2.31) are very similar to the ones reported therein (2.05, 2.07 and 2.31,^[6]^ for visual comparison see Figure 5 bottom). This behavior is explained very nicely by the observations of previous crystallographic analyses and ^1^H ENDOR spectroscopy studies that account for a water molecule in the second coordination sphere of Cu_C_, accounting for a similarly weak interaction of **[1‐Cu]PF_6_** to water, and thereby again a big commonality. NMR reveals slight changes of the resonances of **[1‐Cu](PF_6_)** in the presence of water, the diffusion coefficient remains the same (see Figures S27 and S28). These results demonstrate nicely that the cage‐complexes shown here display spectroscopic features similar to those of the pMMO Cu_C_ site.

**Figure 5:**
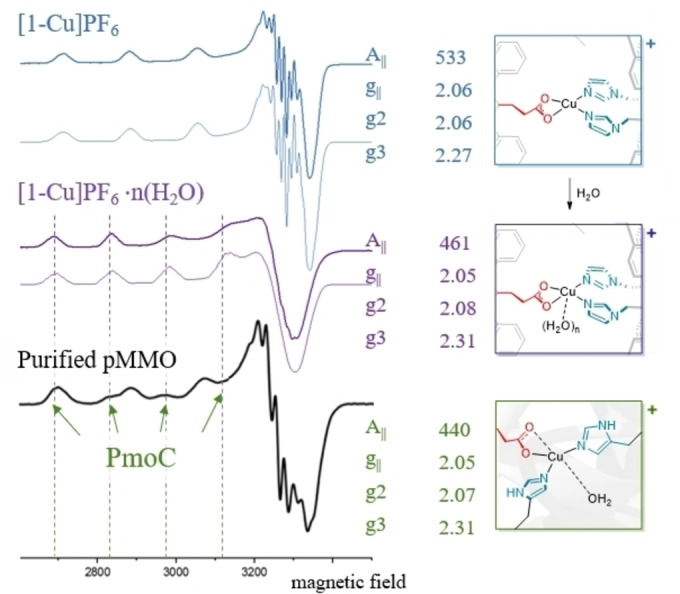
CW X‐band EPR spectra (bold) and simulations (light) of **[1‐Cu]PF_6_**, **[1‐Cu]PF_6_** ⋅ ***n*** **(H_2_O**) in toluene at 147 K and 9.4 GHz and purified Mc. sp. str. Rockwell pMMO (Reprinted with permission from ref. [6]. Copyright **2021** American Chemical Society) with selected simulation parameters.

To conclude, we present an endohedrally functionalized cage as a chelating ligand to copper, giving rise to the first study on a structural mimic of the Cu_C_ site of pMMO. The mimic is exceptionally accurate through the detailed structural replication and the embedment within a cavity. This results in copper complexes with facile accessibility of both Cu^I^ and Cu^II^. Solid‐state and computational analysis of **1‐Cu** and the THF adduct of **[1‐Cu]PF_6_** confirm the proposed structures with each two imidazole and one carboxylate function coordinating to the copper ion, and the ability to adopt different coordination modes. The complexes are capable of catalytically oxidizing biorelevant hydroquinones such as TMHQ under aerobic conditions. Additionally, the secondary amines seem to be catalytically active as well. The possibility for an interplay of the metal center and remote functional groups underlines our cage design to be a conceptually nice mimic of enzymatic active site pockets. In the absence of hydroquinones, we observe backbone oxidation of the ligand upon exposure to molecular oxygen, accounting for its activity also in CH activation. Moreover, EPR analysis of the oxidized complex in the presence of water reveals striking similarities to the natural paragon. Overall, we believe that our conceptual approach towards unprecedented synthetic coordination spheres results in a kind of imitation that is exceptionally accurate and can therefore contribute to a better understanding of enzymatic processes.

## Conflict of interest

The authors declare no conflict of interest.

## Supporting information

As a service to our authors and readers, this journal provides supporting information supplied by the authors. Such materials are peer reviewed and may be re‐organized for online delivery, but are not copy‐edited or typeset. Technical support issues arising from supporting information (other than missing files) should be addressed to the authors.

Supporting Information

Supporting Information

Supporting Information

Supporting Information

Supporting Information

## Data Availability

The data that support the findings of this study are available in the Supporting Information of this article.
