# Supplementary material for: A Copper Cage‐Complex as Mimic of the pMMO CuC Site
Source: Angew Chem Int Ed Engl. 2022 Jul 19;61(35):e202206120. doi: 10.1002/anie.202206120 (PMC9544873; doi:10.1002/anie.202206120)

checkCIF/PLATON report

Structure factors have been supplied for datablock(s) SB_220404_MO

THIS REPORT IS FOR GUIDANCE ONLY. IF USED AS PART OF A REVIEW PROCEDURE FOR PUBLICATION, IT SHOULD NOT REPLACE THE EXPERTISE OF AN EXPERIENCED CRYSTALLOGRAPHIC REFEREE.

No syntax errors found. CIF dictionary Interpreting this report

Datablock: SB_220404_MO

```
Bond precision:   C-C = 0.0212 A          Wavelength=0.71073

Cell:             a=25.785(2)     b=22.8050(18)       c=18.2362(14)
                  alpha=90        beta=101.118(3)     gamma=90
Temperature:      100 K
```

	Calculated	Reported
Volume	10522.1(14)	10522.1(15)
Space group	C 2	C 1 2 1
Hall group	C 2y	C 2y
Moiety formula	C101 H107 Cu N10 O2 [+ solvent]	?
Sum formula	C101 H107 Cu N10 O2 [+ solvent]	C101 H107 Cu N10 O2
Mr	1556.52	1556.50
Dx,g cm-3	0.983	0.983
Z	4	4
Mu (mm-1)	0.254	0.254
F000	3312.0	3312.0
F000'	3314.33	
h,k,lmax	27,24,19	27,24,19
Nref	13068[6738]	12813
Tmin,Tmax	0.987,0.989	0.910,0.990
Tmin'	0.986	

```
Correction method= # Reported T Limits: Tmin=0.910 Tmax=0.990
AbsCorr = MULTI-SCAN

Data completeness= 1.90/0.98        Theta(max)= 22.080
```

R(reflections)= 0.1076(10518) wR2(reflections)= 0.3113(12813)

S = 1.079 Npar= 986

The following ALERTS were generated. Each ALERT has the format
test-name_ALERT_alert-type_alert-level.
Click on the hyperlinks for more details of the test.

Alert level A

```
SHFSU01_ALERT_2_A  The absolute value of parameter shift to su ratio > 0.20
            Absolute value of the parameter shift to su ratio given   1.957
            Additional refinement cycles may be required.
THETM01_ALERT_3_A  The value of sine(theta_max)/wavelength is less than 0.550
            Calculated sin(theta_max)/wavelength =    0.5289
PLAT080_ALERT_2_A Maximum Shift/Error ............................       1.96 Why ?
PLAT203_ALERT_2_A Negative Isotropic ADP for H906      ...........     -0.001 Report
PLAT203_ALERT_2_A Negative Isotropic ADP for H910      ...........     -0.001 Report
PLAT213_ALERT_2_A Atom C45             has ADP max/min Ratio .....        5.8 prolat
PLAT213_ALERT_2_A Atom C54             has ADP max/min Ratio .....        5.7 oblate
PLAT213_ALERT_2_A Atom C89             has ADP max/min Ratio .....        6.0 oblate
PLAT414_ALERT_2_A Short Intra D-H..H-X       H17A      ..H905     .       1.61 Ang.
                                                 x,y,z  =      1_555 Check
PLAT414_ALERT_2_A Short Intra D-H..H-X       H80B      ..H905     .       1.67 Ang.
                                                 x,y,z  =      1_555 Check
PLAT770_ALERT_2_A Suspect C-H Bond in CIF:  C17      --H905      .       1.54 Ang.
PLAT770_ALERT_2_A Suspect C-H Bond in CIF:  C80      --H905      .       1.53 Ang.
```

Alert level B

```
PLAT213_ALERT_2_B Atom C32             has ADP max/min Ratio .....        4.8 prolat
PLAT213_ALERT_2_B Atom C71             has ADP max/min Ratio .....        4.3 prolat
PLAT213_ALERT_2_B Atom C91             has ADP max/min Ratio .....        4.1 oblate
PLAT341_ALERT_3_B Low Bond Precision on  C-C Bonds ..............    0.02124 Ang.
PLAT350_ALERT_3_B Short  C-H (X0.96,N1.08A)  C99       - H908     .       0.72 Ang.
PLAT410_ALERT_2_B Short Intra H...H Contact  H78B      ..H80A     .       1.88 Ang.
                                                 x,y,z  =      1_555 Check
PLAT414_ALERT_2_B Short Intra D-H..H-X       H64A      ..H909     .       1.83 Ang.
                                                 x,y,z  =      1_555 Check
PLAT414_ALERT_2_B Short Intra D-H..H-X       H83B      ..H909     .       1.82 Ang.
                                                 x,y,z  =      1_555 Check
PLAT987_ALERT_1_B The Flack x is >> 0 -  Do a BASF/TWIN Refinement     Please Check
```

Alert level C

```
STRVA01_ALERT_4_C           Flack test results are ambiguous.
           From the CIF: _refine_ls_abs_structure_Flack    0.509
           From the CIF: _refine_ls_abs_structure_Flack_su    0.009
PLAT082_ALERT_2_C High R1 Value ..................................       0.11 Report
PLAT084_ALERT_3_C High wR2 Value (i.e. > 0.25) ...................       0.31 Report
PLAT090_ALERT_3_C Poor Data / Parameter Ratio (Zmax > 18) ........       6.77 Note
PLAT213_ALERT_2_C Atom C13             has ADP max/min Ratio .....        3.1 prolat
PLAT213_ALERT_2_C Atom C31             has ADP max/min Ratio .....        3.9 oblate
PLAT213_ALERT_2_C Atom C56             has ADP max/min Ratio .....        3.4 prolat
PLAT213_ALERT_2_C Atom C64             has ADP max/min Ratio .....        3.4 prolat
```

```
PLAT220_ALERT_2_C NonSolvent   Resd 1  C   Ueq(max)/Ueq(min) Range        4.4 Ratio
PLAT222_ALERT_3_C NonSolvent Resd 1  H   Uiso(max)/Uiso(min) Range       10.0 Ratio
PLAT234_ALERT_4_C Large Hirshfeld Difference N2       --C3       .       0.21 Ang.
PLAT234_ALERT_4_C Large Hirshfeld Difference N5       --C17      .       0.18 Ang.
PLAT234_ALERT_4_C Large Hirshfeld Difference N6       --C24      .       0.16 Ang.
PLAT234_ALERT_4_C Large Hirshfeld Difference N7       --C41      .       0.20 Ang.
PLAT234_ALERT_4_C Large Hirshfeld Difference N8       --C48      .       0.16 Ang.
PLAT234_ALERT_4_C Large Hirshfeld Difference N9       --C64      .       0.18 Ang.
PLAT234_ALERT_4_C Large Hirshfeld Difference N10      --C71      .       0.19 Ang.
PLAT234_ALERT_4_C Large Hirshfeld Difference C2       --C3       .       0.18 Ang.
PLAT234_ALERT_4_C Large Hirshfeld Difference C4       --C5       .       0.19 Ang.
PLAT234_ALERT_4_C Large Hirshfeld Difference C7       --C8       .       0.20 Ang.
PLAT234_ALERT_4_C Large Hirshfeld Difference C8       --C9       .       0.20 Ang.
PLAT234_ALERT_4_C Large Hirshfeld Difference C9       --C10      .       0.17 Ang.
PLAT234_ALERT_4_C Large Hirshfeld Difference C11      --C12      .       0.18 Ang.
PLAT234_ALERT_4_C Large Hirshfeld Difference C13      --C14      .       0.20 Ang.
PLAT234_ALERT_4_C Large Hirshfeld Difference C37      --C38      .       0.18 Ang.
PLAT234_ALERT_4_C Large Hirshfeld Difference C52      --C53      .       0.19 Ang.
PLAT234_ALERT_4_C Large Hirshfeld Difference C55      --C56      .       0.16 Ang.
PLAT234_ALERT_4_C Large Hirshfeld Difference C87      --C92      .       0.19 Ang.
PLAT234_ALERT_4_C Large Hirshfeld Difference C88      --C89      .       0.19 Ang.
PLAT234_ALERT_4_C Large Hirshfeld Difference C90      --C91      .       0.18 Ang.
PLAT234_ALERT_4_C Large Hirshfeld Difference C91      --C92      .       0.18 Ang.
PLAT241_ALERT_2_C High   'MainMol' Ueq as Compared to Neighbors of         C2 Check
PLAT241_ALERT_2_C High   'MainMol' Ueq as Compared to Neighbors of        C13 Check
PLAT241_ALERT_2_C High   'MainMol' Ueq as Compared to Neighbors of        C39 Check
PLAT241_ALERT_2_C High   'MainMol' Ueq as Compared to Neighbors of        C43 Check
PLAT241_ALERT_2_C High   'MainMol' Ueq as Compared to Neighbors of        C46 Check
PLAT241_ALERT_2_C High   'MainMol' Ueq as Compared to Neighbors of        C62 Check
PLAT241_ALERT_2_C High   'MainMol' Ueq as Compared to Neighbors of        C64 Check
PLAT241_ALERT_2_C High   'MainMol' Ueq as Compared to Neighbors of        C70 Check
PLAT242_ALERT_2_C Low    'MainMol' Ueq as Compared to Neighbors of         C3 Check
PLAT242_ALERT_2_C Low    'MainMol' Ueq as Compared to Neighbors of        C10 Check
PLAT242_ALERT_2_C Low    'MainMol' Ueq as Compared to Neighbors of        C11 Check
PLAT242_ALERT_2_C Low    'MainMol' Ueq as Compared to Neighbors of        C14 Check
PLAT242_ALERT_2_C Low    'MainMol' Ueq as Compared to Neighbors of        C21 Check
PLAT242_ALERT_2_C Low    'MainMol' Ueq as Compared to Neighbors of        C29 Check
PLAT242_ALERT_2_C Low    'MainMol' Ueq as Compared to Neighbors of        C38 Check
PLAT242_ALERT_2_C Low    'MainMol' Ueq as Compared to Neighbors of        C52 Check
PLAT242_ALERT_2_C Low    'MainMol' Ueq as Compared to Neighbors of        C61 Check
PLAT242_ALERT_2_C Low    'MainMol' Ueq as Compared to Neighbors of        C65 Check
PLAT242_ALERT_2_C Low    'MainMol' Ueq as Compared to Neighbors of        C69 Check
PLAT245_ALERT_2_C U(iso) H907      Smaller than U(eq) N7        by      0.029 Ang**2
PLAT245_ALERT_2_C U(iso) H909      Smaller than U(eq) N9        by      0.049 Ang**2
PLAT303_ALERT_2_C Full Occupancy Atom H908      with # Connections       2.00 Check
PLAT334_ALERT_2_C Small Aver. Benzene C-C Dist C11      -C16             1.37 Ang.
PLAT334_ALERT_2_C Small Aver. Benzene C-C Dist C18      -C23             1.37 Ang.
PLAT360_ALERT_2_C Short  C(sp3)-C(sp3) Bond  C78      - C79      .       1.43 Ang.
PLAT362_ALERT_2_C Short  C(sp3)-C(sp2) Bond  C38      - C41      .       1.38 Ang.
PLAT369_ALERT_2_C Long   C(sp2)-C(sp2) Bond  C6       - C11      .       1.53 Ang.
PLAT369_ALERT_2_C Long   C(sp2)-C(sp2) Bond  C34      - C35      .       1.53 Ang.
PLAT410_ALERT_2_C Short Intra H...H Contact  H93B     ..H94A     .       1.97 Ang.
                                                      x,y,z  =      1_555 Check
PLAT414_ALERT_2_C Short Intra D-H..H-X       H82A     ..H905     .       1.93 Ang.
                                                      x,y,z  =      1_555 Check
PLAT420_ALERT_2_C D-H Bond Without Acceptor  N5       --H905     .     Please Check
PLAT420_ALERT_2_C D-H Bond Without Acceptor  N6       --H906     .     Please Check
```

```
PLAT420_ALERT_2_C D-H Bond Without Acceptor  N7       --H907     .     Please Check
PLAT420_ALERT_2_C D-H Bond Without Acceptor  N8       --H908     .     Please Check
PLAT420_ALERT_2_C D-H Bond Without Acceptor  N9       --H909     .     Please Check
PLAT420_ALERT_2_C D-H Bond Without Acceptor  N10      --H910     .     Please Check
PLAT907_ALERT_2_C Flack x > 0.5, Structure Needs to be Inverted? .       0.51 Check
PLAT910_ALERT_3_C Missing # of FCF Reflection(s) Below Theta(Min).          7 Note
PLAT911_ALERT_3_C Missing FCF Refl Between Thmin & STh/L=    0.529         58 Report
PLAT934_ALERT_3_C Number of (Iobs-Icalc)/Sigma(W) > 10 Outliers ..          1 Check
```

Alert level G

```
PLAT002_ALERT_2_G Number of Distance or Angle Restraints on AtSite         28 Note
PLAT003_ALERT_2_G Number of Uiso or Uij Restrained non-H Atoms ...          6 Report
PLAT033_ALERT_4_G Flack x Value Deviates > 3.0 * sigma from Zero .      0.509 Note
PLAT072_ALERT_2_G SHELXL First  Parameter in WGHT  Unusually Large       0.15 Report
PLAT083_ALERT_2_G SHELXL Second Parameter in WGHT  Unusually Large      65.72 Why ?
PLAT111_ALERT_2_G ADDSYM Detects New (Pseudo) Centre of Symmetry .        100 %Fit
PLAT112_ALERT_2_G ADDSYM Detects New (Pseudo) Symm. Elem         m        100 %Fit
PLAT113_ALERT_2_G ADDSYM Suggests Possible Pseudo/New  Space Group       C2/m Check
PLAT171_ALERT_4_G The CIF-Embedded .res File Contains EADP Records          3 Report
PLAT172_ALERT_4_G The CIF-Embedded .res File Contains DFIX Records          4 Report
PLAT176_ALERT_4_G The CIF-Embedded .res File Contains SADI Records          7 Report
PLAT178_ALERT_4_G The CIF-Embedded .res File Contains SIMU Records          1 Report
PLAT187_ALERT_4_G The CIF-Embedded .res File Contains RIGU Records          6 Report
PLAT230_ALERT_2_G Hirshfeld Test Diff for    N2       --C1A      .        5.1 s.u.
PLAT230_ALERT_2_G Hirshfeld Test Diff for    C51A     --C52      .       12.0 s.u.
PLAT230_ALERT_2_G Hirshfeld Test Diff for    C52      --C28      .       10.3 s.u.
PLAT301_ALERT_3_G Main Residue  Disorder ..............(Resd  1  )        12% Note
PLAT335_ALERT_2_G Check Large C6 Ring C-C Range C5       -C10            0.18 Ang.
PLAT335_ALERT_2_G Check Large C6 Ring C-C Range C58      -C63            0.16 Ang.
PLAT335_ALERT_2_G Check Large C6 Ring C-C Range C65      -C70            0.15 Ang.
PLAT343_ALERT_2_G Unusual          Angle Range in Main Residue for        C99 Check
PLAT410_ALERT_2_G Short Intra H...H Contact  H28D     ..H40      .       2.14 Ang.
                                                  x,y,z  =      1_555 Check
PLAT606_ALERT_4_G Solvent Accessible VOID(S) in Structure ........          ! Info
PLAT720_ALERT_4_G Number of Unusual/Non-Standard Labels ..........          2 Note
PLAT722_ALERT_1_G Angle   Calc      109.00, Rep      113.20 Dev...       4.20 Degree
              C29     -C51     -H51A       1_555  1_555  1_555     #  405 Check
PLAT722_ALERT_1_G Angle   Calc      110.00, Rep      113.10 Dev...       3.10 Degree
              C50     -C51     -H51A       1_555  1_555  1_555     #  406 Check
PLAT722_ALERT_1_G Angle   Calc      108.00, Rep      104.10 Dev...       3.90 Degree
              C29     -C51     -H51B       1_555  1_555  1_555     #  407 Check
PLAT722_ALERT_1_G Angle   Calc      108.00, Rep      104.10 Dev...       3.90 Degree
              C50     -C51     -H51B       1_555  1_555  1_555     #  408 Check
PLAT722_ALERT_1_G Angle   Calc      105.00, Rep      100.70 Dev...       4.30 Degree
              C51     -C50     -H50A       1_555  1_555  1_555     #  412 Check
PLAT722_ALERT_1_G Angle   Calc      105.00, Rep      109.60 Dev...       4.60 Degree
              C51     -C50     -H50B       1_555  1_555  1_555     #  414 Check
PLAT722_ALERT_1_G Angle   Calc      110.00, Rep      106.60 Dev...       3.40 Degree
              N4      -C28     -H28A       1_555  1_555  1_555     #  420 Check
PLAT722_ALERT_1_G Angle   Calc      109.00, Rep      106.50 Dev...       2.50 Degree
              C52     -C28     -H28A       1_555  1_555  1_555     #  421 Check
PLAT722_ALERT_1_G Angle   Calc      111.00, Rep      113.00 Dev...       2.00 Degree
              N4      -C28     -H28B       1_555  1_555  1_555     #  422 Check
PLAT722_ALERT_1_G Angle   Calc      110.00, Rep      112.80 Dev...       2.80 Degree
              C52     -C28     -H28B       1_555  1_555  1_555     #  423 Check
PLAT722_ALERT_1_G Angle   Calc      108.00, Rep      110.50 Dev...       2.50 Degree
```

```
              C50A    -C51A    -H51C       1_555  1_555  1_555     #  458 Check
PLAT722_ALERT_1_G Angle   Calc      108.00, Rep      110.10 Dev...       2.10 Degree
              C52     -C51A    -H51C       1_555  1_555  1_555     #  459 Check
PLAT722_ALERT_1_G Angle   Calc      109.00, Rep      106.40 Dev...       2.60 Degree
              C50A    -C51A    -H51D       1_555  1_555  1_555     #  460 Check
PLAT722_ALERT_1_G Angle   Calc      109.00, Rep      106.30 Dev...       2.70 Degree
              C52     -C51A    -H51D       1_555  1_555  1_555     #  461 Check
PLAT722_ALERT_1_G Angle   Calc      110.00, Rep      112.90 Dev...       2.90 Degree
              N4A     -C28A    -H28C       1_555  1_555  1_555     #  474 Check
PLAT722_ALERT_1_G Angle   Calc      109.00, Rep      112.90 Dev...       3.90 Degree
              C29     -C28A    -H28C       1_555  1_555  1_555     #  475 Check
PLAT722_ALERT_1_G Angle   Calc      110.00, Rep      106.60 Dev...       3.40 Degree
              N4A     -C28A    -H28D       1_555  1_555  1_555     #  476 Check
PLAT722_ALERT_1_G Angle   Calc      110.00, Rep      106.20 Dev...       3.80 Degree
              C29     -C28A    -H28D       1_555  1_555  1_555     #  477 Check
PLAT722_ALERT_1_G Angle   Calc      107.00, Rep      109.40 Dev...       2.40 Degree
              C51A    -C50A    -H50C       1_555  1_555  1_555     #  496 Check
PLAT722_ALERT_1_G Angle   Calc      108.00, Rep      105.40 Dev...       2.60 Degree
              C51A    -C50A    -H50D       1_555  1_555  1_555     #  498 Check
PLAT767_ALERT_4_G INS Embedded LIST 6 Instruction Should be LIST 4     Please Check
PLAT773_ALERT_2_G Check long C-C Bond in CIF: C26      --C25             1.91 Ang.
PLAT773_ALERT_2_G Check long C-C Bond in CIF: C27      --C25             2.01 Ang.
PLAT779_ALERT_4_G Suspect or Irrelevant (Bond) Angle(s) in CIF ...      19.00 Deg.
              C99  -N8   -H908    1_555   1_555   1_555 ......     #   42 Check
PLAT779_ALERT_4_G Suspect or Irrelevant (Bond) Angle(s) in CIF ...      34.00 Deg.
              N5   -C17  -H905    1_555   1_555   1_555 ......     #   75 Check
PLAT779_ALERT_4_G Suspect or Irrelevant (Bond) Angle(s) in CIF ...      33.00 Deg.
              N5   -C80  -H905    1_555   1_555   1_555 ......     #  275 Check
PLAT779_ALERT_4_G Suspect or Irrelevant (Bond) Angle(s) in CIF ...      23.00 Deg.
              N8   -C99  -H908    1_555   1_555   1_555 ......     #  366 Check
PLAT779_ALERT_4_G Suspect or Irrelevant (Bond) Angle(s) in CIF ...      37.00 Deg.
              N3   -C26  -C25     1_555   1_555   1_555 ......     #  426 Check
PLAT779_ALERT_4_G Suspect or Irrelevant (Bond) Angle(s) in CIF ...      41.20 Deg.
              N4   -C27  -C25     1_555   1_555   1_555 ......     #  433 Check
PLAT779_ALERT_4_G Suspect or Irrelevant (Bond) Angle(s) in CIF ...      36.00 Deg.
              N3   -C25  -C26     1_555   1_555   1_555 ......     #  444 Check
PLAT779_ALERT_4_G Suspect or Irrelevant (Bond) Angle(s) in CIF ...      42.50 Deg.
              C26  -C25  -C27     1_555   1_555   1_555 ......     #  448 Check
PLAT860_ALERT_3_G Number of Least-Squares Restraints ............        438 Note
PLAT869_ALERT_4_G ALERTS Related to the Use of SQUEEZE  Suppressed         ! Info
PLAT883_ALERT_1_G No Info/Value for _atom_sites_solution_primary .     Please Do !
PLAT909_ALERT_3_G Percentage of I>2sig(I) Data at Theta(Max) Still        72% Note
PLAT978_ALERT_2_G Number C-C Bonds with Positive Residual Density.          2 Info
```

---

12 **ALERT level A** = Most likely a serious problem - resolve or explain
9 **ALERT level B** = A potentially serious problem, consider carefully
71 **ALERT level C** = Check. Ensure it is not caused by an omission or oversight
60 **ALERT level G** = General information/check it is not something unexpected

22 ALERT type 1 CIF construction/syntax error, inconsistent or missing data
78 ALERT type 2 Indicator that the structure model may be wrong or deficient
12 ALERT type 3 Indicator that the structure quality may be low
40 ALERT type 4 Improvement, methodology, query or suggestion
0 ALERT type 5 Informative message, check

It is advisable to attempt to resolve as many as possible of the alerts in all categories. Often the minor alerts point to easily fixed oversights, errors and omissions in your CIF or refinement strategy, so attention to these fine details can be worthwhile. In order to resolve some of the more serious problems it may be necessary to carry out additional measurements or structure refinements. However, the purpose of your study may justify the reported deviations and the more serious of these should normally be commented upon in the discussion or experimental section of a paper or in the "special_details" fields of the CIF. checkCIF was carefully designed to identify outliers and unusual parameters, but every test has its limitations and alerts that are not important in a particular case may appear. Conversely, the absence of alerts does not guarantee there are no aspects of the results needing attention. It is up to the individual to critically assess their own results and, if necessary, seek expert advice.

Publication of your CIF in IUCr journals

A basic structural check has been run on your CIF. These basic checks will be run on all CIFs submitted for publication in IUCr journals (*Acta Crystallographica*, *Journal of Applied Crystallography*, *Journal of Synchrotron Radiation*); however, if you intend to submit to *Acta Crystallographica Section C* or *E* or *IUCrData*, you should make sure that full publication checks are run on the final version of your CIF prior to submission.

Publication of your CIF in other journals

Please refer to the *Notes for Authors* of the relevant journal for any special instructions relating to CIF submission.

PLATON version of 19/02/2022; check.def file version of 19/02/2022

Datablock SB_220404_MO - ellipsoid plot

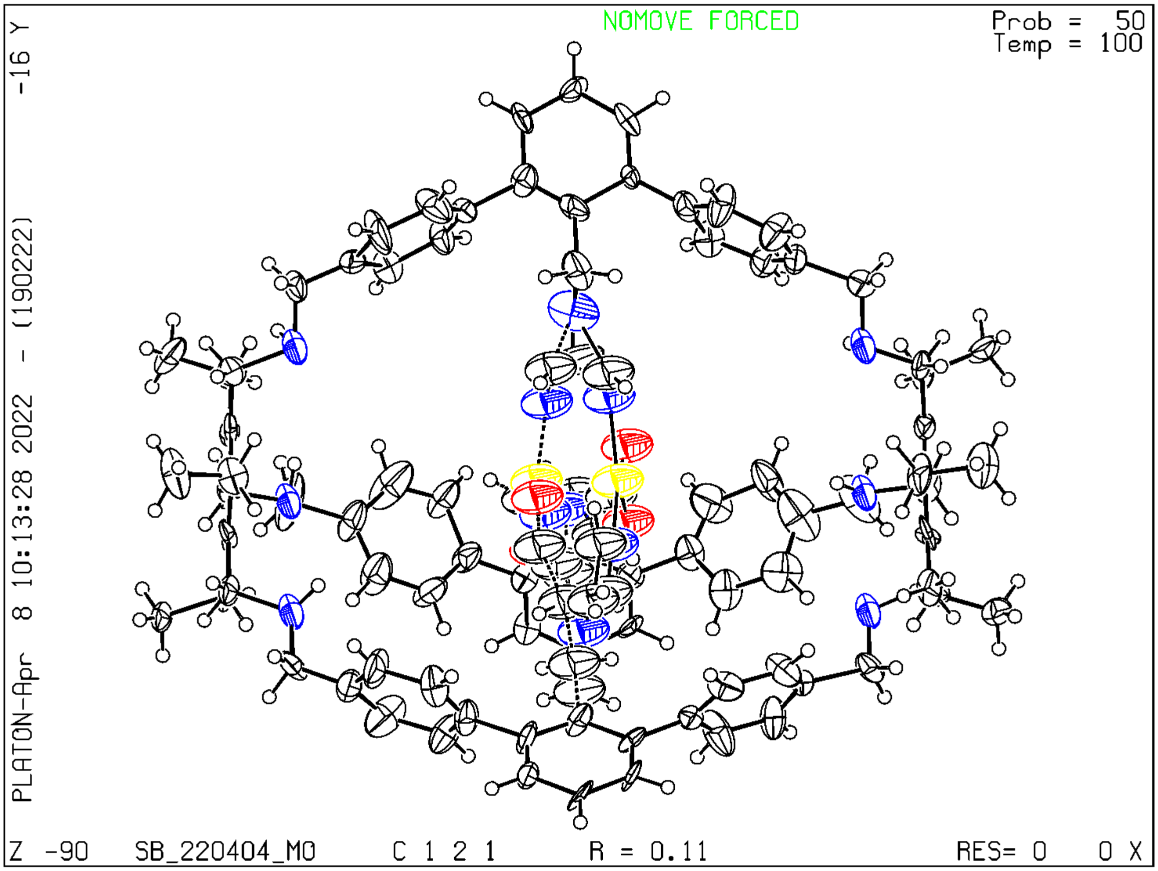

Supplement: Supplementary file 2 — Supporting Information [file ANIE-61-0-s005.pdf]
