# Supplementary material for: A Copper Cage‐Complex as Mimic of the pMMO CuC Site
Source: Angew Chem Int Ed Engl. 2022 Jul 19;61(35):e202206120. doi: 10.1002/anie.202206120 (PMC9544873; doi:10.1002/anie.202206120)

checkCIF/PLATON report

Structure factors have been supplied for datablock(s) SB_040322_4_MO

THIS REPORT IS FOR GUIDANCE ONLY. IF USED AS PART OF A REVIEW PROCEDURE FOR PUBLICATION, IT SHOULD NOT REPLACE THE EXPERTISE OF AN EXPERIENCED CRYSTALLOGRAPHIC REFEREE.

No syntax errors found. CIF dictionary Interpreting this report

Datablock: SB_040322_4_MO

```
Bond precision:   C-C = 0.0230 A          Wavelength=0.71073

Cell:           a=21.8689(18)      b=23.944(2)        c=26.510(2)
                alpha=94.580(4)    beta=108.970(4)    gamma=99.957(4)
Temperature:    100 K
```

	Calculated	Reported
Volume	12791.1(18)	12791.1(19)
Space group	P -1	P -1
Hall group	-P 1	-P 1
Moiety formula	C109 H122 Cu N10 O4, C109 H124 Cu N10 O4, 2(F6 P) [+ solvent]	?
Sum formula	C218 H246 Cu2 F12 N20 O8 P2 [+ solvent]	C218 H246 Cu2 F12 N20 O8 P2
Mr	3691.40	3691.44
Dx,g cm-3	0.958	0.958
Z	2	2
Mu (mm-1)	0.236	0.236
F000	3908.0	3908.0
F000’	3911.28	
h,k,lmax	21,23,26	21,23,25
Nref	25891	24753
Tmin,Tmax	0.956,0.981	0.950,0.980
Tmin’	0.946	

```
Correction method= # Reported T Limits: Tmin=0.950 Tmax=0.980
AbsCorr = MULTI-SCAN

Data completeness= 0.956            Theta(max)= 20.570
```

R(reflections)= 0.2061(20069)   wR2(reflections)= 0.5932(24753)

S = 3.037   Npar= 1916

The following ALERTS were generated. Each ALERT has the format
test-name_ALERT_alert-type_alert-level.
Click on the hyperlinks for more details of the test.

Alert level A

```
SHFSU01_ALERT_2_A  The absolute value of parameter shift to su ratio > 0.20
            Absolute value of the parameter shift to su ratio given   3.290
            Additional refinement cycles may be required.
THETM01_ALERT_3_A  The value of sine(theta_max)/wavelength is less than 0.550
            Calculated sin(theta_max)/wavelength =    0.4944
PLAT080_ALERT_2_A Maximum Shift/Error ...........................       3.29 Why ?
PLAT082_ALERT_2_A High R1 Value .................................       0.21 Report
PLAT084_ALERT_3_A High wR2 Value (i.e. > 0.25) ..................       0.59 Report
PLAT213_ALERT_2_A Atom C18             has ADP max/min Ratio .....       7.2 prolat
PLAT213_ALERT_2_A Atom C69             has ADP max/min Ratio .....       5.3 oblate
PLAT213_ALERT_2_A Atom C140            has ADP max/min Ratio .....       6.6 prolat
PLAT316_ALERT_2_A Too many H on C in C=N Moiety in Main Residue ..      C165 Check
PLAT934_ALERT_3_A Number of (Iobs-Icalc)/Sigma(W) > 10 Outliers ..       153 Check
```

Alert level B

```
PLAT029_ALERT_3_B _diffrn_measured_fraction_theta_full value Low .     0.956 Why?
PLAT097_ALERT_2_B Large Reported Max.  (Positive) Residual Density      3.93 eA-3
PLAT213_ALERT_2_B Atom C19             has ADP max/min Ratio .....       4.7 prolat
PLAT213_ALERT_2_B Atom C83             has ADP max/min Ratio .....       4.6 prolat
PLAT213_ALERT_2_B Atom C91             has ADP max/min Ratio .....       4.8 prolat
PLAT213_ALERT_2_B Atom C93             has ADP max/min Ratio .....       4.6 prolat
PLAT213_ALERT_2_B Atom C204            has ADP max/min Ratio .....       4.1 prolat
PLAT213_ALERT_2_B Atom C202            has ADP max/min Ratio .....       4.5 prolat
PLAT213_ALERT_2_B Atom C214            has ADP max/min Ratio .....       4.1 prolat
PLAT220_ALERT_2_B NonSolvent   Resd 1  C   Ueq(max)/Ueq(min) Range       7.3 Ratio
PLAT220_ALERT_2_B NonSolvent   Resd 2  C   Ueq(max)/Ueq(min) Range       9.0 Ratio
PLAT241_ALERT_2_B High   'MainMol' Ueq as Compared to Neighbors of      C204 Check
PLAT241_ALERT_2_B High   'MainMol' Ueq as Compared to Neighbors of      C209 Check
PLAT241_ALERT_2_B High   'MainMol' Ueq as Compared to Neighbors of      C214 Check
PLAT242_ALERT_2_B Low    'MainMol' Ueq as Compared to Neighbors of       C82 Check
PLAT242_ALERT_2_B Low    'MainMol' Ueq as Compared to Neighbors of      C213 Check
PLAT341_ALERT_3_B Low Bond Precision on  C-C Bonds ..............    0.02304 Ang.
PLAT362_ALERT_2_B Short  C(sp3)-C(sp2) Bond  C204      - C205     .       1.26 Ang.
PLAT369_ALERT_2_B Long   C(sp2)-C(sp2) Bond  C158      - C159     .       1.57 Ang.
PLAT410_ALERT_2_B Short Intra H...H Contact  H18G      ..H18H     .       1.86 Ang.
                                                       x,y,z  =      1_555 Check
PLAT414_ALERT_2_B Short Intra D-H..H-X       H10A      ..H908     .       1.85 Ang.
                                                       x,y,z  =      1_555 Check
PLAT414_ALERT_2_B Short Intra D-H..H-X       H16A      ..H905     .       1.85 Ang.
                                                       x,y,z  =      1_555 Check
PLAT414_ALERT_2_B Short Intra D-H..H-X       H23A      ..H906     .       1.87 Ang.
                                                       x,y,z  =      1_555 Check
PLAT414_ALERT_2_B Short Intra D-H..H-X       H78B      ..H905     .       1.88 Ang.
                                                       x,y,z  =      1_555 Check
```

```
PLAT414_ALERT_2_B Short Intra D-H..H-X       H93B     ..H906     .        1.86 Ang.
                                                     x,y,z  =      1_555 Check
PLAT415_ALERT_2_B Short Inter D-H..H-X       H112     ..H916     .        1.93 Ang.
                                                  -x,2-y,1-z  =      2_576 Check
PLAT910_ALERT_3_B Missing # of FCF Reflection(s) Below Theta(Min).          16 Note
```

Alert level C

```
DIFMN02_ALERT_2_C  The minimum difference density is < -0.1*ZMAX*0.75
            _refine_diff_density_min given =     -2.722
            Test value =     -2.175
DIFMN03_ALERT_1_C  The minimum difference density is < -0.1*ZMAX*0.75
            The relevant atom site should be identified.
DIFMX02_ALERT_1_C  The maximum difference density is > 0.1*ZMAX*0.75
            The relevant atom site should be identified.
GOODF01_ALERT_2_C  The least squares goodness of fit parameter lies
            outside the range 0.80 <> 2.00
            Goodness of fit given =      3.037
PLAT087_ALERT_2_C Unsatisfactory S value (Too High) .............        3.04 Check
PLAT098_ALERT_2_C Large Reported Min.  (Negative) Residual Density      -2.72 eA-3
PLAT213_ALERT_2_C Atom C10              has ADP max/min Ratio .....       4.0 oblate
PLAT213_ALERT_2_C Atom C20              has ADP max/min Ratio .....       3.6 oblate
PLAT213_ALERT_2_C Atom C49              has ADP max/min Ratio .....       3.3 prolat
PLAT213_ALERT_2_C Atom C88              has ADP max/min Ratio .....       3.2 oblate
PLAT213_ALERT_2_C Atom N11              has ADP max/min Ratio .....       3.3 oblate
PLAT213_ALERT_2_C Atom C103             has ADP max/min Ratio .....       3.6 prolat
PLAT213_ALERT_2_C Atom C116             has ADP max/min Ratio .....       3.1 prolat
PLAT213_ALERT_2_C Atom C186             has ADP max/min Ratio .....       3.2 prolat
PLAT213_ALERT_2_C Atom C192             has ADP max/min Ratio .....       3.6 oblate
PLAT213_ALERT_2_C Atom C193             has ADP max/min Ratio .....       3.1 prolat
PLAT213_ALERT_2_C Atom C212             has ADP max/min Ratio .....       3.1 prolat
PLAT213_ALERT_2_C Atom C213             has ADP max/min Ratio .....       4.0 prolat
PLAT222_ALERT_3_C NonSolvent Resd 1  H   Uiso(max)/Uiso(min) Range       10.0 Ratio
PLAT222_ALERT_3_C NonSolvent Resd 2  H   Uiso(max)/Uiso(min) Range       10.0 Ratio
PLAT241_ALERT_2_C High   'MainMol' Ueq as Compared to Neighbors of        C40 Check
PLAT241_ALERT_2_C High   'MainMol' Ueq as Compared to Neighbors of        C64 Check
PLAT241_ALERT_2_C High   'MainMol' Ueq as Compared to Neighbors of        C67 Check
PLAT241_ALERT_2_C High   'MainMol' Ueq as Compared to Neighbors of        C69 Check
PLAT241_ALERT_2_C High   'MainMol' Ueq as Compared to Neighbors of       C207 Check
PLAT241_ALERT_2_C High   'MainMol' Ueq as Compared to Neighbors of       C112 Check
PLAT241_ALERT_2_C High   'MainMol' Ueq as Compared to Neighbors of       C148 Check
PLAT241_ALERT_2_C High   'MainMol' Ueq as Compared to Neighbors of       C163 Check
PLAT241_ALERT_2_C High   'MainMol' Ueq as Compared to Neighbors of       C211 Check
PLAT241_ALERT_2_C High   'MainMol' Ueq as Compared to Neighbors of       C212 Check
PLAT242_ALERT_2_C Low    'MainMol' Ueq as Compared to Neighbors of         O6 Check
PLAT242_ALERT_2_C Low    'MainMol' Ueq as Compared to Neighbors of        C20 Check
PLAT242_ALERT_2_C Low    'MainMol' Ueq as Compared to Neighbors of        C68 Check
PLAT242_ALERT_2_C Low    'MainMol' Ueq as Compared to Neighbors of        C94 Check
PLAT242_ALERT_2_C Low    'MainMol' Ueq as Compared to Neighbors of       C203 Check
PLAT242_ALERT_2_C Low    'MainMol' Ueq as Compared to Neighbors of       C206 Check
PLAT242_ALERT_2_C Low    'MainMol' Ueq as Compared to Neighbors of       C208 Check
PLAT242_ALERT_2_C Low    'MainMol' Ueq as Compared to Neighbors of        Cu2 Check
PLAT242_ALERT_2_C Low    'MainMol' Ueq as Compared to Neighbors of         O7 Check
PLAT242_ALERT_2_C Low    'MainMol' Ueq as Compared to Neighbors of       C111 Check
PLAT242_ALERT_2_C Low    'MainMol' Ueq as Compared to Neighbors of       C120 Check
PLAT242_ALERT_2_C Low    'MainMol' Ueq as Compared to Neighbors of       C164 Check
PLAT242_ALERT_2_C Low    'MainMol' Ueq as Compared to Neighbors of       C169 Check
```

```
PLAT242_ALERT_2_C Low    'MainMol' Ueq as Compared to Neighbors of       C177 Check
PLAT245_ALERT_2_C U(iso) H909      Smaller than U(eq) N9        by      0.021 Ang**2
PLAT245_ALERT_2_C U(iso) H21J      Smaller than U(eq) C214      by      0.040 Ang**2
PLAT245_ALERT_2_C U(iso) H21R      Smaller than U(eq) C218      by      0.046 Ang**2
PLAT245_ALERT_2_C U(iso) H917      Smaller than U(eq) N17       by      0.026 Ang**2
PLAT245_ALERT_2_C U(iso) H919      Smaller than U(eq) N19       by      0.028 Ang**2
PLAT260_ALERT_2_C Large Average Ueq of Residue Including        P1      0.109 Check
PLAT260_ALERT_2_C Large Average Ueq of Residue Including        P2      0.109 Check
PLAT360_ALERT_2_C Short  C(sp3)-C(sp3) Bond  C212     - C213     .       1.42 Ang.
PLAT360_ALERT_2_C Short  C(sp3)-C(sp3) Bond  C213     - C214     .       1.43 Ang.
PLAT362_ALERT_2_C Short  C(sp3)-C(sp2) Bond  C152     - C153     .       1.39 Ang.
PLAT362_ALERT_2_C Short  C(sp3)-C(sp2) Bond  C156     - C157     .       1.35 Ang.
PLAT369_ALERT_2_C Long   C(sp2)-C(sp2) Bond  C9       - C10      .       1.54 Ang.
PLAT410_ALERT_2_C Short Intra H...H Contact  H27A     ..H42      .       1.98 Ang.
                                                      x,y,z  =     1_555 Check
PLAT410_ALERT_2_C Short Intra H...H Contact  H81B     ..H82A     .       1.97 Ang.
                                                      x,y,z  =     1_555 Check
PLAT410_ALERT_2_C Short Intra H...H Contact  H82B     ..H84A     .       1.91 Ang.
                                                      x,y,z  =     1_555 Check
PLAT410_ALERT_2_C Short Intra H...H Contact  H97B     ..H99A     .       1.99 Ang.
                                                      x,y,z  =     1_555 Check
PLAT410_ALERT_2_C Short Intra H...H Contact  H18N     ..H18O     .       1.97 Ang.
                                                      x,y,z  =     1_555 Check
PLAT410_ALERT_2_C Short Intra H...H Contact  H19H     ..H19K     .       1.97 Ang.
                                                      x,y,z  =     1_555 Check
PLAT413_ALERT_2_C Short Inter XH3 .. XHn     H18R     ..H23B     .       2.14 Ang.
                                                    x,y,1+z  =     1_556 Check
PLAT414_ALERT_2_C Short Intra D-H..H-X       H47A     ..H908     .       1.99 Ang.
                                                      x,y,z  =     1_555 Check
PLAT414_ALERT_2_C Short Intra D-H..H-X       H64B     ..H909     .       1.95 Ang.
                                                      x,y,z  =     1_555 Check
PLAT414_ALERT_2_C Short Intra D-H..H-X       H97A     ..H910     .       1.95 Ang.
                                                      x,y,z  =     1_555 Check
PLAT420_ALERT_2_C D-H Bond Without Acceptor  N5       --H905     .    Please Check
PLAT420_ALERT_2_C D-H Bond Without Acceptor  N6       --H906     .    Please Check
PLAT420_ALERT_2_C D-H Bond Without Acceptor  N7       --H907     .    Please Check
PLAT420_ALERT_2_C D-H Bond Without Acceptor  N8       --H908     .    Please Check
PLAT420_ALERT_2_C D-H Bond Without Acceptor  N9       --H909     .    Please Check
PLAT420_ALERT_2_C D-H Bond Without Acceptor  N10      --H910     .    Please Check
PLAT420_ALERT_2_C D-H Bond Without Acceptor  N15      --H915     .    Please Check
PLAT420_ALERT_2_C D-H Bond Without Acceptor  N16      --H916     .    Please Check
PLAT420_ALERT_2_C D-H Bond Without Acceptor  N17      --H917     .    Please Check
PLAT420_ALERT_2_C D-H Bond Without Acceptor  N18      --H918     .    Please Check
PLAT420_ALERT_2_C D-H Bond Without Acceptor  N19      --H919     .    Please Check
PLAT420_ALERT_2_C D-H Bond Without Acceptor  N20      --H920     .    Please Check
PLAT906_ALERT_3_C Large K Value in the Analysis of Variance ......      7.488 Check
PLAT906_ALERT_3_C Large K Value in the Analysis of Variance ......      2.529 Check
PLAT911_ALERT_3_C Missing FCF Refl Between Thmin & STh/L=    0.494       1096 Report
PLAT918_ALERT_3_C Reflection(s) with I(obs) much Smaller I(calc) .         33 Check
PLAT973_ALERT_2_C Check Calcd Positive Resid. Density on       Cu2       1.05 eA-3
PLAT977_ALERT_2_C Check Negative Difference Density on H15A      .      -0.34 eA-3
PLAT977_ALERT_2_C Check Negative Difference Density on H15C      .      -0.42 eA-3
PLAT977_ALERT_2_C Check Negative Difference Density on H15D      .      -0.37 eA-3
PLAT977_ALERT_2_C Check Negative Difference Density on H20M      .      -0.34 eA-3
PLAT977_ALERT_2_C Check Negative Difference Density on H21C      .      -0.40 eA-3
PLAT977_ALERT_2_C Check Negative Difference Density on H21J      .      -0.61 eA-3
PLAT977_ALERT_2_C Check Negative Difference Density on H21Q      .      -0.33 eA-3
```

```
PLAT977_ALERT_2_C Check Negative Difference Density on H51A      .      -0.31 eA-3
PLAT977_ALERT_2_C Check Negative Difference Density on H71B      .      -0.38 eA-3
PLAT977_ALERT_2_C Check Negative Difference Density on H83B      .      -0.36 eA-3
PLAT977_ALERT_2_C Check Negative Difference Density on H83C      .      -0.48 eA-3
PLAT977_ALERT_2_C Check Negative Difference Density on H96B      .      -0.43 eA-3
PLAT977_ALERT_2_C Check Negative Difference Density on H168      .      -0.39 eA-3
PLAT977_ALERT_2_C Check Negative Difference Density on H906      .      -0.41 eA-3
```

Alert level G

```
PLAT002_ALERT_2_G Number of Distance or Angle Restraints on AtSite         72 Note
PLAT066_ALERT_1_G Predicted and Reported Tmin&Tmax Range Identical          ? Check
PLAT072_ALERT_2_G SHELXL First  Parameter in WGHT  Unusually Large       0.20 Report
PLAT154_ALERT_1_G The s.u.'s on the Cell Angles are Equal ..(Note)      0.004 Degree
PLAT171_ALERT_4_G The CIF-Embedded .res File Contains EADP Records         13 Report
PLAT172_ALERT_4_G The CIF-Embedded .res File Contains DFIX Records          4 Report
PLAT175_ALERT_4_G The CIF-Embedded .res File Contains SAME Records          1 Report
PLAT176_ALERT_4_G The CIF-Embedded .res File Contains SADI Records          7 Report
PLAT180_ALERT_4_G Check Cell Rounding: # of Values Ending with 0 =          3 Note
PLAT187_ALERT_4_G The CIF-Embedded .res File Contains RIGU Records          1 Report
PLAT301_ALERT_3_G Main Residue  Disorder .............(Resd  1  )          1% Note
PLAT302_ALERT_4_G Anion/Solvent/Minor-Residue Disorder (Resd  3  )        57% Note
PLAT333_ALERT_2_G Large Aver C6-Ring C-C Dist C87      -C92      .       1.42 Ang.
PLAT333_ALERT_2_G Large Aver C6-Ring C-C Dist C135     -C140     .       1.42 Ang.
PLAT335_ALERT_2_G Check Large C6 Ring C-C Range C4       -C9             0.15 Ang.
PLAT335_ALERT_2_G Check Large C6 Ring C-C Range C52      -C57            0.15 Ang.
PLAT335_ALERT_2_G Check Large C6 Ring C-C Range C65      -C70            0.18 Ang.
PLAT335_ALERT_2_G Check Large C6 Ring C-C Range C87      -C92            0.17 Ang.
PLAT335_ALERT_2_G Check Large C6 Ring C-C Range C111     -C116           0.19 Ang.
PLAT414_ALERT_2_G Short Intra D-H..H-X       H40A     ..H977     .       1.60 Ang.
                                                  x,y,z  =     1_555 Check
PLAT414_ALERT_2_G Short Intra D-H..H-X       H82B     ..H977     .       1.99 Ang.
                                                  x,y,z  =     1_555 Check
PLAT414_ALERT_2_G Short Intra D-H..H-X       H84A     ..H977     .       1.57 Ang.
                                                  x,y,z  =     1_555 Check
PLAT432_ALERT_2_G Short Inter X...Y Contact  F2       ..C49      .       2.97 Ang.
                                                  x,y,z  =     1_555 Check
PLAT432_ALERT_2_G Short Inter X...Y Contact  F10      ..C40      .       2.89 Ang.
                                                  x,y,z  =     1_555 Check
PLAT432_ALERT_2_G Short Inter X...Y Contact  F2A      ..C49      .       2.76 Ang.
                                                  x,y,z  =     1_555 Check
PLAT432_ALERT_2_G Short Inter X...Y Contact  F2A      ..C50      .       2.83 Ang.
                                                  x,y,z  =     1_555 Check
PLAT432_ALERT_2_G Short Inter X...Y Contact  F6A      ..C146     .       2.92 Ang.
                                                  x,y,z  =     1_555 Check
PLAT606_ALERT_4_G Solvent Accessible VOID(S) in Structure ........          ! Info
PLAT767_ALERT_4_G INS Embedded LIST 6 Instruction Should be LIST 4     Please Check
PLAT794_ALERT_5_G Tentative Bond Valency for Cu1       (III)     .       2.64 Info
PLAT794_ALERT_5_G Tentative Bond Valency for Cu2       (III)     .       2.67 Info
PLAT860_ALERT_3_G Number of Least-Squares Restraints ............         100 Note
PLAT869_ALERT_4_G ALERTS Related to the Use of SQUEEZE  Suppressed          ! Info
PLAT883_ALERT_1_G No Info/Value for _atom_sites_solution_primary .     Please Do !
PLAT909_ALERT_3_G Percentage of I>2sig(I) Data at Theta(Max) Still        57% Note
PLAT913_ALERT_3_G Missing # of Very Strong Reflections in FCF ....          1 Note
PLAT930_ALERT_2_G FCF-based   Twin Law ( 0 1-1)         Est.d BASF       0.27 Check
PLAT931_ALERT_5_G CIFcalcFCF  Twin Law ( 0 1-1)         Est.d BASF       0.33 Check
PLAT978_ALERT_2_G Number C-C Bonds with Positive Residual Density.          0 Info
```

```
 10 ALERT level A = Most likely a serious problem - resolve or explain
 27 ALERT level B = A potentially serious problem, consider carefully
 97 ALERT level C = Check. Ensure it is not caused by an omission or oversight
 39 ALERT level G = General information/check it is not something unexpected

  5 ALERT type 1 CIF construction/syntax error, inconsistent or missing data
139 ALERT type 2 Indicator that the structure model may be wrong or deficient
 16 ALERT type 3 Indicator that the structure quality may be low
 10 ALERT type 4 Improvement, methodology, query or suggestion
  3 ALERT type 5 Informative message, check
```

It is advisable to attempt to resolve as many as possible of the alerts in all categories. Often the minor alerts point to easily fixed oversights, errors and omissions in your CIF or refinement strategy, so attention to these fine details can be worthwhile. In order to resolve some of the more serious problems it may be necessary to carry out additional measurements or structure refinements. However, the purpose of your study may justify the reported deviations and the more serious of these should normally be commented upon in the discussion or experimental section of a paper or in the "special_details" fields of the CIF. checkCIF was carefully designed to identify outliers and unusual parameters, but every test has its limitations and alerts that are not important in a particular case may appear. Conversely, the absence of alerts does not guarantee there are no aspects of the results needing attention. It is up to the individual to critically assess their own results and, if necessary, seek expert advice.

Publication of your CIF in IUCr journals

A basic structural check has been run on your CIF. These basic checks will be run on all CIFs submitted for publication in IUCr journals (*Acta Crystallographica*, *Journal of Applied Crystallography*, *Journal of Synchrotron Radiation*); however, if you intend to submit to *Acta Crystallographica Section C* or *E* or *IUCrData*, you should make sure that full publication checks are run on the final version of your CIF prior to submission.

Publication of your CIF in other journals

Please refer to the *Notes for Authors* of the relevant journal for any special instructions relating to CIF submission.

PLATON version of 19/02/2022; check.def file version of 19/02/2022

Datablock SB_040322_4_MO - ellipsoid plot

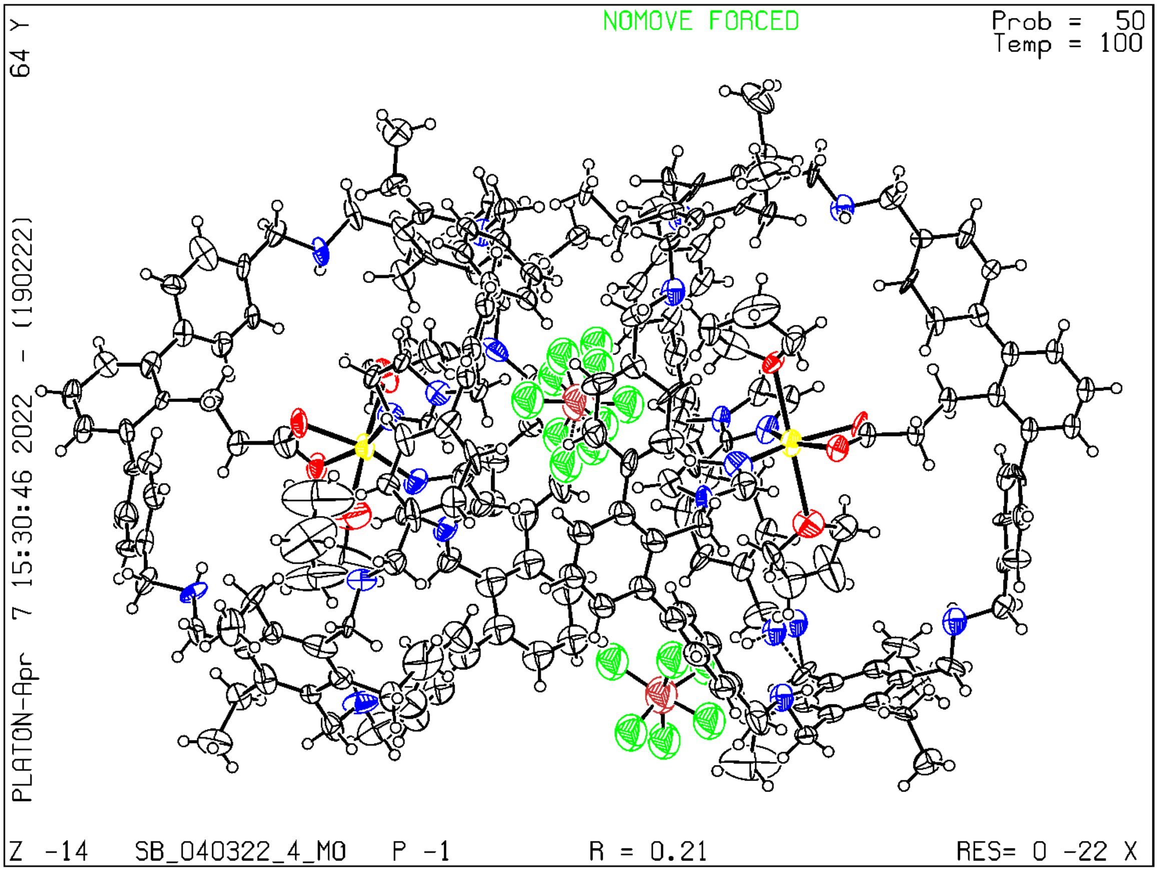

Supplement: Supplementary file 4 — Supporting Information [file ANIE-61-0-s003.pdf]
