# Supplementary material for: A Copper Cage‐Complex as Mimic of the pMMO CuC Site
Source: Angew Chem Int Ed Engl. 2022 Jul 19;61(35):e202206120. doi: 10.1002/anie.202206120 (PMC9544873; doi:10.1002/anie.202206120)
Supplement: Supplementary file 5 — Supporting Information [file ANIE-61-0-s004.pdf]

Angewandte Chemie
Eine Zeitschrift der Gesellschaft Deutscher Chemiker

Supporting Information

A Copper Cage-Complex as Mimic of the pMMO Cu_C Site

*S. C. Bete, L. K. May, P. Woite, M. Roemelt, M. Otte**

Table of Contents

Table of Contents 2
Experimental Procedures 3
General Information 3
Synthesis and discussion of 1 3
Synthesis of **3** 3
Synthesis of **4** 4
Synthesis of **1** 5
Discussion of the analytical data of **1** 5
Synthesis of 1-Cu 6
Synthesis of [1-Cu]PF_6 7
EPR simulation data 7
Synthesis of [1-Cu]PF_6·n(H_2O) 7
EPR simulation data 7
Reaction procedure for dioxygen exposure of 1-Cu 8
General procedure for aerobic oxidation of hydroquinones 8
Spectra 9
X-ray Single-Crystal Structure Analysis 31
Crystallographic Details 31
X-ray Single-Crystal Structure Analysis of 1-Cu. 31
X-ray Single-Crystal Structure Analysis of [1-Cu]PF_6· 2(THF). 33
Comparison of data obtained from X-ray Single-Crystal Structure Analysis of 1-Cu and [1-Cu]PF_6· 2(THF) with data obtained from pMMO 34
Computational Methods 35
References 36
Author Contributions 36

Experimental Procedures

General Information

All reactions involving air- or moisture sensitive compounds were carried out under argon using either standard Schlenk and vacuum line techniques or in UNILAB Glovebox from MBraun. All reagents were purchased from commercial sources and used as received unless otherwise stated. Methanol was stored over molecular sieves for two days and destilled afterwards. Tetrahydrofurane (THF), dichloromethane (DCM), acetonitrile and benzene were taken from a MBRAUN MB SPS-800 solvent purification system. Deuterated DCM (CD_2Cl_2) and benzene (C_6D_6) were degassed using freeze pump thaw cycles and dried over calcium hydride and NaK, respectively. Deuterated tetrahydrofurane (THF-d_8) and Methanol (CD_3OD) were degassed using the freeze thaw pump cycles and subsequently stored over molecular sieves. Compounds **2**[1] and **5**[2] were synthesized as described in literature.
^{1}H and ^{13}C NMR spectra were recorded on a Bruker Avance 300, Avance 400, Avance 500 or Avance 600 spectrometer at 25 °C. ^{1}H and ^{13}C NMR chemical shifts are reported in ppm relative to TMS using the residual solvent resonance as internal standard. Infrared spectra were recorded using an ALPHA Platinum-ATR FTIR spectrometer from Bruker. X-band EPR spectra were recorded on a *Bruker ELEXSYS-II E500* CW-EPR spectrometer equipped with a digital temperature control system (*ER 4131VT*) using liquid nitrogen as coolant. The recorded data were simulated using the *EasySpin* package for *Matlab* (v5.2.28).[3] Liquid samples were prepared in an argon-filled glovebox and filled into air-tight, Teflon stoppered quartz glass *J-Young* NMR or EPR tubes. MS measurements were performed at the analytic laboratory of the chemistry department.

Synthesis and discussion of 1

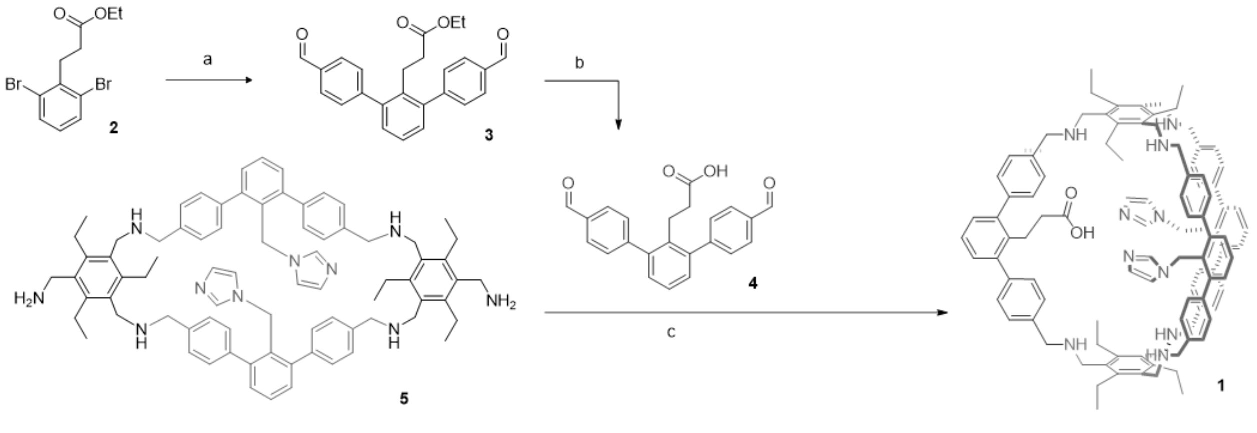

Scheme S1. Synthesis route towards **1**.
We started with literature known **2** that can undergo a Suzuki coupling to give the dialdehyde and ester-functionalized building block **3** with 81% yield. Acid-catalyzed ester hydrolysis quantitatively yields the corresponding carboxylic acid **4** that was further reacted with macrocycle **5** to give **1** in 72% yield.

Synthesis of ***3***

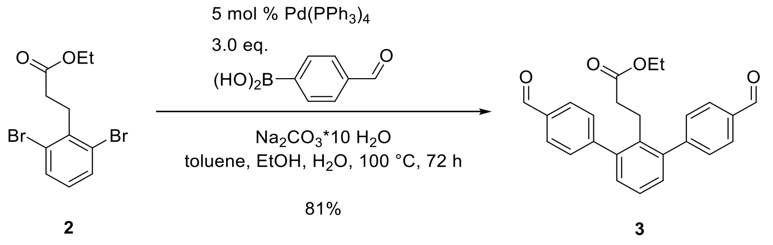

Scheme S2. Synthesis of **3**.

Toluene (70 mL), ethanol (35 mL) and water (15 mL) were added to a 2-necked Schlenk flask under argon. **2** (3.06 g, 9.11 mmol, 1.00 eq), 4-formylphenylboronic acid (4.10 mg, 27.3 mmol, 3.00 eq) and Na_2CO_3*10H_2O (20.8 g, 800 mmol, 87.8 eq) were added to the mixture. While stirring, argon was bubbled through the mixture for 30 minutes. $Pd(PPh_3)_4$ (1.05 g, 0.911 mmol, 5 mol%) was added and the mixture was stirred under argon for 72 h at 100 °C (oil bath temperature). Afterwards, the mixture was allowed to cool to room temperature. Water (100 mL) and ethyl acetate (100 mL) were added and the phases were separated. The organic phase was washed with water (2 x 50 mL) and dried over $MgSO_4$. The solvent of the filtrate was removed under reduced pressure to give the crude product that was extracted with DCM (400 ml) and treated with aqueous potassium hydroxide solution (2.5 %, 400 ml), the phases were separated and the organic phase was washed with water (3 x 100 ml). The product was obtained as a white solid (2.84 g, 7.35 mmol, 81%).

^{1}H NMR (400 MHz, CD2Cl2) δ 10.07 (s, 2H), 7.96 (d, J = 8.0 Hz, 4H), 7.56 (d, J = 7.8 Hz, 4H), 7.37 (t, J = 7.6 Hz, 1H), 7.25 (d, J = 7.6 Hz, 2H), 3.82 (q, J = 7.2 Hz, 2H), 3.01 – 2.80 (m, 2H), 2.11 – 1.95 (m, 2H), 1.02 (t, J = 7.2 Hz, 3H).

^{13}C NMR (101 MHz, CD_2Cl_2) δ 192.18, 172.07, 148.43, 142.30, 142.00, 135.79, 135.73, 130.39, 130.28, 129.97, 126.60, 60.52, 34.68, 25.56, 14.18.

IR (ATR-FTIR): 2990 (w), 2900 (w), 2850 (w), 2750 (w), 1720, 1700 (s), 1605, 1565, 1455, 1430, 1385, 1360, 1300, 1265, 1240, 1205 (s), 1195, 1165, 1100, 1075, 1035, 1015, 1010, 995, 950, 840, 830, 810, 790 (s), 760, 730, 695, 650, 625, 600, 570, 530,495.

Exact mass ESI MS: $C_{25}H_{22}O_4$ $[M+H]^+$ calculated: 387.1591 found: 387.1584.

*Synthesis of **4***

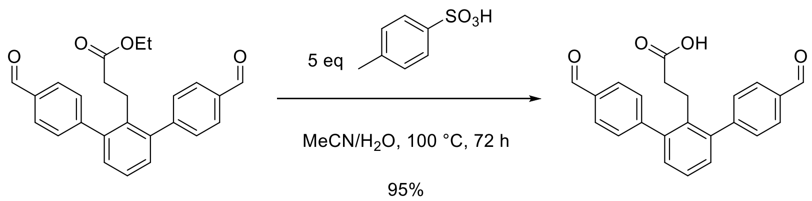

3 **4**

Scheme S3. Synthesis of 4.

3 (487 mg, 1.26 mmol, 1.00 eq) was dissolved in acetonitrile (200 ml) and placed in a 2-necked Schlenk flask under argon. Water (200 ml) was added and argon was bubbled through the mixture for 30 minutes. p-Toluene sulfonic acid monohydrate (1.09 g, 6.31 mmol, 5.00 eq) was added and the reaction mixture was heated to 100 °C and stirred for 72 h. After cooling, acetonitrile was evaporated and the resulting suspension was filtered. The precipitate was washed with water (2 x 100 ml) and extracted with acetonitrile. After evaporating the solvent, the residue was collected with dichloromethane, precipitated with pentane and stored at 5 °C for 2 hours. The product was collected as a colourless crystalline solid (429 mg, 1.20 mmol, 95%).

^{1}H NMR (400 MHz, CD_2Cl_2) δ 10.05 (s, 2H), 7.93 (d, J = 7.7 Hz, 4H), 7.53 (d, J = 7.7 Hz, 4H), 7.37 (t, J = 7.6 Hz, 1H), 7.24 (d, J = 7.5 Hz, 2H), 2.96 – 2.77 (m, 2H), 2.13 – 1.96 (m, 2H).

^{13}C NMR (101 MHz, CD_2Cl_2) δ 192.24, 177.50, 148.30, 142.29, 135.80, 135.20, 130.35, 130.32, 130.02, 126.75, 34.18, 25.20.

IR (ATR-FTIR): 3030 (w), 2963 (w), 2905 (w), 2845 (w), 2730 (w), 2685 (w), 1695 (s), 1605, 1580, 1565, 1455, 1435, 1400, 1385, 1350, 1300, 1285, 1265, 1245, 1205 (s),1170, 1105, 1080, 1035, 1010, 975, 935, 850, 830 (s), 800, 785, 775, 740, 730, 685, 645, 630, 605, 580, 550, 510, 500.

Exact mass ESI MS: $C_{23}H_{18}O_4$ $[M-H]^-$ calculated: 357.1132 found: 357.1132.

*Synthesis of **1***

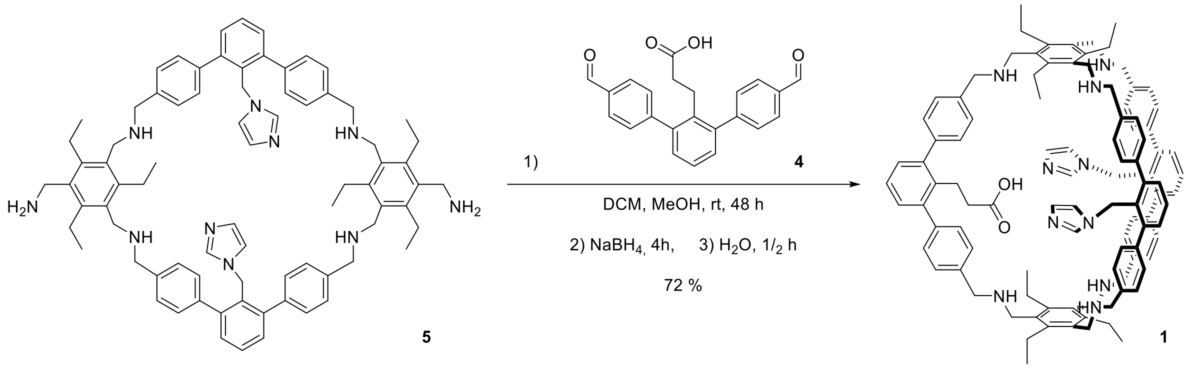

Scheme S4. Synthesis of **1**.

5 (507 mg, 0.434 mmol, 1.00 Äq.) and **4** (156 mg, 0.435 mmol, 1.00 Äq.) were placed in a Schlenk flask under argon. Dry dichloromethane (40 mL) and dry methanol (6 mL) were added and the reaction mixture was stirred for 48 h at room temperature. $NaBH_4$ (81.3 mg, 2.15 mmol, 4.95 Äq.) was added and the resulting mixture was stirred for 4 h at room temperature. After addition of water (40 ml) the mixture was stirred for 10 minutes and the organic solvent was removed in vacuo. The precipitate was filtered off, washed with water (2 x 20 ml), acetonitrile (2 x 20 ml) and extracted with a mixture of dichloromethane (20 ml) and methanol (5 ml). The solvents were removed under vacuum and the solid was collected with DCM, and afterwards treated with concentrated hydrochloric acid (20 ml). The mixture was filtered and the residue was washed with dichloromethane (2 x 10 ml), HCl (aq, conc, 5 ml) and afterwards extracted with diluted hydrochloric acid (50 ml). Triethylamine was added until a solid precipitated from the basic solution that was filtered off. The solid was washed with Triethylamine in water (5%, 2 x 20 ml), acetonitrile (2 x 10 ml), diethyl ether (20 ml) and finally extracted with benzene (50 ml). The product was obtained as a white solid (465 mg, 0.311 mmol, 72%).

^{1}H NMR (500 MHz, C_6D_6) δ 15.08 (s, 1H), 7.38 – 7.35 (m, 4H), 7.31 – 7.23 (m, 13H), 7.19 (d, J = 7.5 Hz, 2H), 7.13 – 7.03 (m, 8H), 6.94 (d, J = 8.1 Hz, 8H), 6.82 (6.82 (t, J = 1.3 Hz, 2H), 6.59 (t, J = 1.1 Hz, 2H), 5.94 (t, J = 1.4 Hz, 2H), 4.68 (s, 4H), 3.94 (s, 4H), 3.93 (s, 4H), 3.86 (s, 4H), 3.79 (s, 4H), 3.77 (s, 4H), 3.76 (s, 4H), 3.36 – 3.30 (m, 2H), 3.27 (q, J = 7.4 Hz, 4H), 3.00 – 2.84 (m, 8H), 2.40 – 2.34 (m, 2H), 1.42 (t, J = 7.5 Hz, 6H), 1.34 (t, J = 7.5 Hz, 12H).

^{1}H DOSY NMR (500 MHz, C_6D_6): $D = 3.78 *10^{-10}\ m^2\ s^{-1}$.

^{13}C NMR (126 MHz, C_6D_6) δ 174.08, 144.51, 143.40, 142.92, 142.74, 141.62, 140.47, 139.70, 139.63, 137.31, 137.11, 134.57, 134.36, 130.60, 129.58, 129.02, 128.68, 128.44, 128.34, 128.25, 128.15, 125.90, 118.32, 55.75, 55.44, 49.14, 48.94, 45.28, 35.58, 26.14, 23.05, 22.98, 17.51, 17.45.

IR (ATR-FTIR): 3320 (w), 3050 (w), 3020 (w), 2960 (m), 2925 (m), 2865 (m), 1710 (m br), 1610, 1580, 1265, 1510, 1450 (s), 1400, 1370, 1355, 1290, 1225, 1180, 1155,1105 (s), 1075 (s), 1020, 1010, 995, 940, 905, 850, 800, 775, 725, 705, 660, 615, 560, 535.

Exact mass ESI MS: $C_{101}H_{109}N_{10}O_2$ $[M+H]^+$ calculated: 1493.8729 found: 1493.8720.

*Discussion of the analytical data of **1***

In the ^{1}H NMR spectrum of **1**, three triplets with small coupling constants are detected at chemical shifts of 6.82, 6.82 and 6.59 ppm that correspond to either one proton of the two imidazole units, which demonstrates their chemical equivalence on the NMR timescale. In the main article, Figure 2, top, these signals as well as the one for the methylene linker are enlightened in turquoise. The multiplets at 3.33 and 2.37 ppm, which are enlightened in red (main article, Figure 2, top), originate from the ethylene linker between the cage backbone and the carboxylic acid group. The presence of the carboxylic acid is further confirmed by a resonance at 174.08 ppm in the ^{13}C NMR and a signal at 1710 cm^{-1} in the IR spectrum (that comply with the carbon resonance at 174.05 ppm and the vibration frequency of 1710 cm^{-1} we observed for the previously reported ligand[2]). Main article, Figure 2 bottom shows the ^{1}H DOSY NMR of **1** in grey. Despite the NMR solvent signal all other observed signals belong to a species with a diffusion coefficient $3.78 \times 10^{-10}\ m^2\ s^{-1}$. Using the Stokes-Einstein equation for spherically shaped molecules and the dynamic viscosity of benzene as 0.6018[4] this diffusion coefficient translates to a hydrodynamic radius of 10.0 Å which is also in good agreement with the one found for the previously reported connatural ligand.[2]

Synthesis of 1-Cu

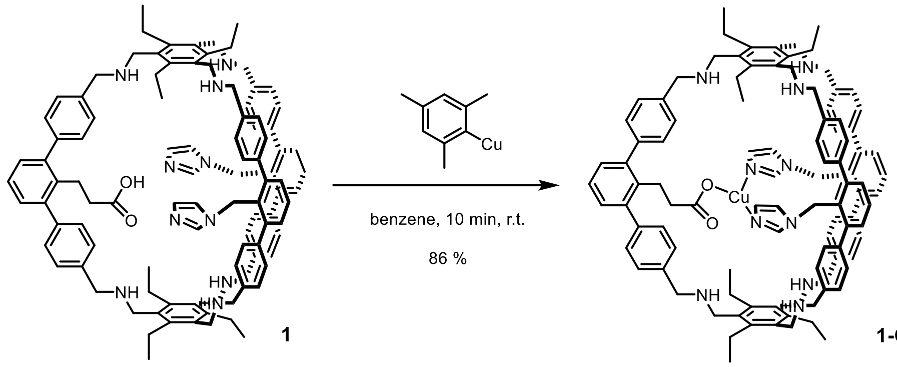

Scheme S5. Synthesis of 1-Cu.

1 (34.4 mg, 23.0 μmol, 1.11 eq) was placed in a vial in a glovebox and benzene (5 ml) was added. While stirring, mesityl copper (3.8 mg, 20.7 μmol, 1.00 eq) in benzene (2 ml) was added dropwise. The suspension was stirred for 10 minutes before it got filtered, and the solvent of the solution was removed afterwards under reduced pressure. The residue is dried in vacuo. Benzene (2 ml) is added, the precipitate was filtered off again and the solvent was again removed. **1-Cu** (27.7 mg, 17.7 μmol, 86%) was obtained as a white solid.

^{1}H NMR (600 MHz, C_6D_6) δ 7.41 (s, 8H), 7.27 (dd, J = 7.8, 2.2 Hz, 12H), 7.13 – 7.08 (m, 4H), 7.08 (d, J = 7.6 Hz, 5H), 6.93 – 6.87 (m, 8H), 6.51 (s, 3H), 6.07 (s, 2H), 5.78 (s, 2H), 4.60 (s, 4H), 3.93 (s, 4H), 3.92 (s, 4H), 3.83 - 3.77 (m, 16H), 3.44 – 3.37 (m, 2H), 3.21 (q, J = 7.5 Hz, 4H), 2.99 – 2.83 (m, 8H), 2.66 – 2.57 (m, 2H), 1.41 (t, J = 7.5 Hz, 6H), 1.33 (t, J = 7.4 Hz, 12H).
^{1}H DOSY NMR (600 MHz, C_6D_6): D = 3.65 *10^{-10} m^2 s^{-1}.

^{13}C NMR (126 MHz, C_6D_6) δ 178.17, 144.47, 144.14, 143.40, 143.16, 142.76, 142.66, 141.91, 140.57, 140.45, 139.63, 139.34, 139.27, 139.25, 137.18, 134.59, 134.31, 134.11, 130.32, 129.71, 129.67, 129.44, 128.35, 128.25, 128.17, 128.06, 127.97, 127.87, 125.37, 118.50, 65.92, 55.83, 55.41, 55.23, 49.18, 49.01, 45.44, 38.14, 34.44, 28.69, 22.99, 22.93, 22.73, 18.00, 17.45, 17.41, 17.37.

After reacting **1** with mesityl copper the resonances of the imidazole units, still integrating to two protons each, are detected at 6.51, 6.07 and 5.78 ppm, and are broadened to singlets. The corresponding methylene group signal shifts less strongly from 4.68 towards 4.60 ppm. The Aryl-bound methylene group shifts from 3.33 to 3.40 ppm, and the carboxyl-bound one from 2.37 to 2.62 ppm, by that, in line with the assignment, showing a stronger upfield shift for the protons more proximate to the metal center.

Exact mass ESI MS: $C_{101}H_{107}N_{10}O_2Cu$ [M-(e$^-$)]$^+$ (observed as Cu(II)): calculated: 1554.7869 found: 1554.7834.

IR (ATR-FTIR): 3140 (w), 3055 (w), 3020 (w), 2960, 2925, 2870, 2815, 1585, 1530, 1510, 1450, 1435, 1405, 1375, 1325, 1300, 1240, 1180, 1045, 1020, 955, 945, 850, 805, 780, 750, 735, 705, 660, 645, 615, 545, 500 (s), 485, 480.

Crystalline material of **1-Cu** was obtained by vapor diffusion of acetonitrile into a solution of the complex in benzene.

Synthesis of $[1\text{-}Cu]PF_6$

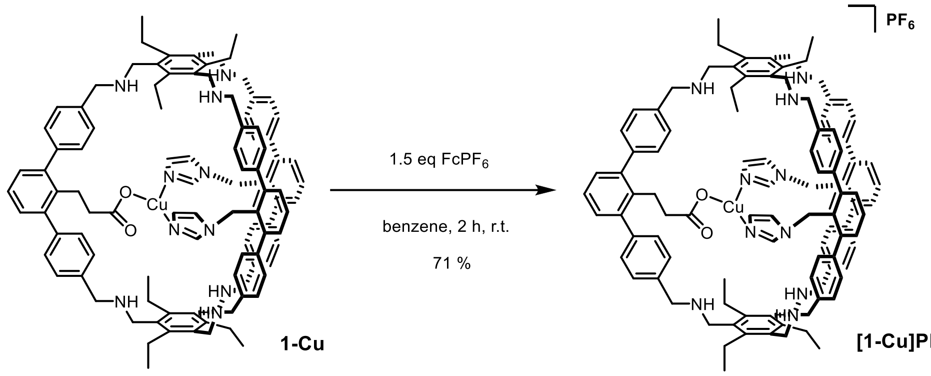

Scheme S6. Synthesis of **$[1\text{-}Cu]PF_6$**.

1-Cu (10.0 mg, 6.39 μmol, 1.00 eq) was placed in a vial in a glovebox and benzene (2 ml) was added. Ferrocenium hexafluorophosphate (9.59 μmol, 1.50 eq) was added and the suspension was stirred for 2 hours before it got filtered, and the solvent of the solution was removed afterwards under reduced pressure. The residue was washed with acetonitrile (2 x 2 ml) and extracted with benzene (2 ml). The solvent was removed to give **$1\text{-}CuPF_6$** (7.6 mg, 4.5 μmol, 71%) as light blue solid.

^{1}H NMR (600 MHz, C_6D_6) δ 7.66, 7.35, 6.79, 6.35, 4.19, 4.09, 4.04, 3.53, 3.42, 1.64, 1.61.

^{1}H DOSY NMR (600 MHz, C_6D_6): $D = 3.65 *10^{-10}$ m^2 s^{-1}.

Exact mass ESI MS: $C_{102}H_{109}N_{10}O_2Cu$ $[M]^+$ calculated: 1554.7869 found: 1554.7841.

IR (ATR-FTIR): 3320 (w), 3140 (w), 3050 (w), 2960, 2925, 2870, 2820, 2280, 1585, 1505, 1455, 1410, 1370, 1330, 1300, 1275, 1240, 1180, 1110, 1090, 1020, 1010, 995, 955, 835 (s), 810 (s), 775, 750, 730, 700, 680, 615, 575, 560, 540 (s), 520.

Crystalline material of **$[1\text{-}Cu]PF_6\cdot 2(THF)$** was obtained by vapor diffusion of pentane into a solution of the complex in THF.

EPR simulation data

S = 1/2; Nucs = 'Cu,N,N'
$g_{||}$ = 2.06 MHz, $g_{\perp,1}$ = 2.06 Hz, $g_{\perp,2}$ = 2.27
^{63}Cu $A_{||}$ = 533 MHz, ^{63}Cu $A_{\perp,1}$ = 61 MHz, ^{63}Cu $A_{\perp,2}$ = 23 MHz
$^{14}N_{1st}$ $A_{||}$ = 33 MHz, $^{14}N_{1st}$ $A_{\perp,1}$ = 38 MHz, $^{14}N_{1st}$ $A_{\perp,2}$ = 33 MHz
$^{14}N_{2nd}$ $A_{||}$ = 38 MHz, $^{14}N_{2nd}$ $A_{\perp,1}$ = 45 MHz, $^{14}N_{2nd}$ $A_{\perp,2}$ = 37 MHz

Synthesis of $[1\text{-}Cu]PF_6\cdot n(H_2O)$

Addition of water to **$[1\text{-}Cu]PF_6$** without further purification leads to a quantitative transformation followed by NMR, EPR and IR.

^{1}H NMR (600 MHz, C_6D_6): δ 7.68, 7.47, 7.06, 6.68, 6.45, 4.31, 4.22, 4.14, 4.05, 3.97, 3.94, 3.79, 3.59, 3.45, 3.26, 2.98, 1.61, 1.41.

^{1}H DOSY NMR (600 MHz, C_6D_6): $D = 3.65 *10^{-10}$ m^2 s^{-1}

EPR simulation data

S = 1/2; Nucs = 'Cu,N,N'
$g_{||}$ = 2.05 MHz, $g_{\perp,1}$ = 2.08 Hz, $g_{\perp,2}$ = 2.31
^{63}Cu $A_{||}$ = 461 MHz, ^{63}Cu $A_{\perp,1}$ = 8 MHz, ^{63}Cu $A_{\perp,2}$ = 4 MHz
$^{14}N_{1st}$ $A_{||}$ = 49 MHz, $^{14}N_{1st}$ $A_{\perp,1}$ = 67 MHz, $^{14}N_{1st}$ $A_{\perp,2}$ = 43 MHz
$^{14}N_{2nd}$ $A_{||}$ = 63 MHz, $^{14}N_{2nd}$ $A_{\perp,1}$ = 58 MHz, $^{14}N_{2nd}$ $A_{\perp,2}$ = 47 MHz

Reaction procedure for dioxygen exposure of 1-Cu

1-Cu (4.5 mg, 1.5 μmol) was placed in a *J-Young* NMR tube and dissolved in C_6D_6. The sample was degassed by two freeze-pump-thaw cycles, cooled to -196 °C, and vacuum was again applied. The sample was exposed to dioxygen gas (0.1 bar overpressure) and afterwards warmed to room temperature while shaking. The sample was analyzed by ^{1}H NMR spectroscopy and mass spectrometry.

General procedure for aerobic oxidation of hydroquinones

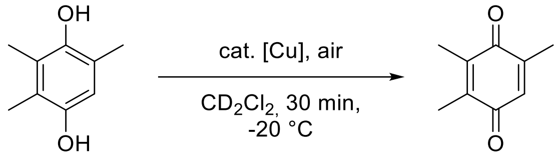

Scheme S7. Aerobic oxidation of trimethylhydroquinone

Trimethylhydroquinone (3.0 mg, 20 μmol) and the potential catalyst (1 μmol) were placed in a 50 ml schlenk tube and after addition of DCM (1 ml), the flask was cooled to -196 °C. Vacuum was applied, the flask was closed and placed in a -20 °C cooling bath. After temperature adjustment, the flask was filled with air by opening the valve and the mixture was stirred vigorously for 20 minutes at -20 °C, before it was again exposed to vacuum to remove O_2 and the solvent without warming up to room temperature. The residue was analyzed by ^{1}H NMR spectroscopy. This method was applied to table 1 entries 1 to 4.

The hydroquinone (20 μmol) and the potential catalyst (1 μmol) were placed in a glove box in a 20 ml vial and after addition of DCM-d_2 (1 ml) the closed vial was taken out of the glove box. The vial was placed in a cooling bath at -196 °C. After temperature adjustment, the vial was opened to air and argon was removed by a syringe. The mixture was stirred vigorously for 20 minutes at -20 °C, before 0.5 mL were placed in a young NMR tube. The solution was frozen and the atmosphere was via applying vacuum changed from air to argon. The solution was immediately analyzed by ^{1}H NMR spectroscopy. This method was applied to table 1 entries 1 and 4-10. For entries 1 and 4 identical results have been obtained for the procedures described.

Spectra

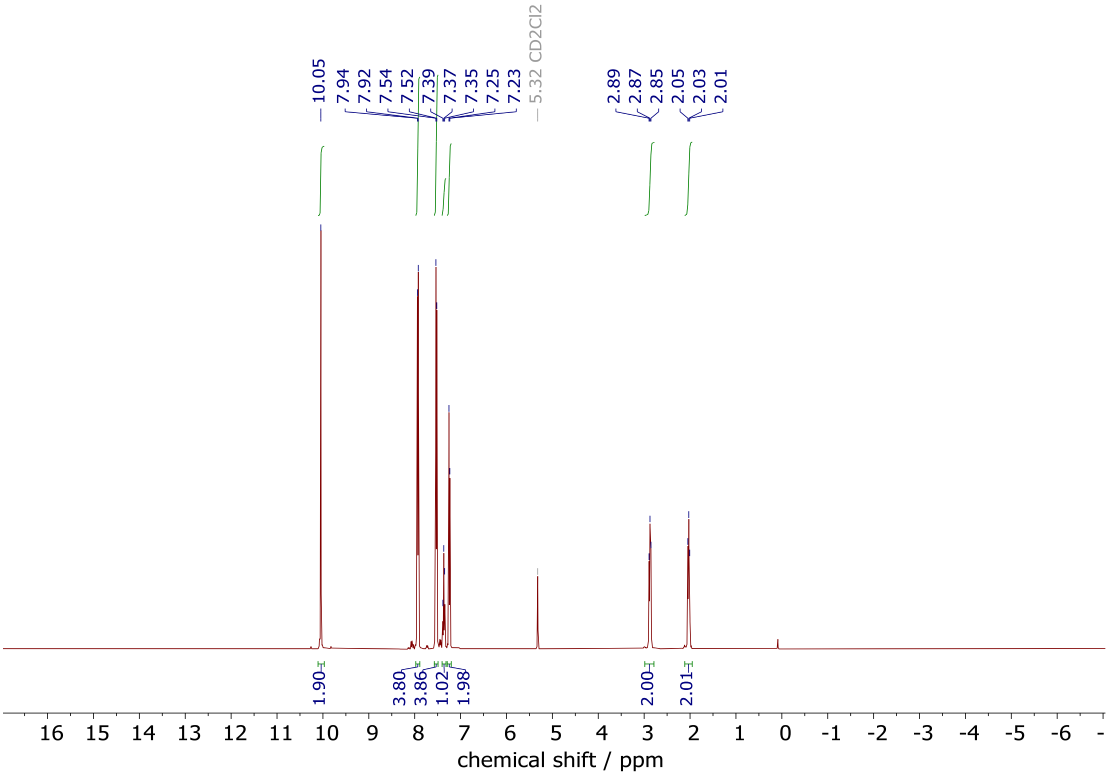

Figure S1. ^{1}H NMR spectrum of **3** in CD_2Cl_2 at rt.

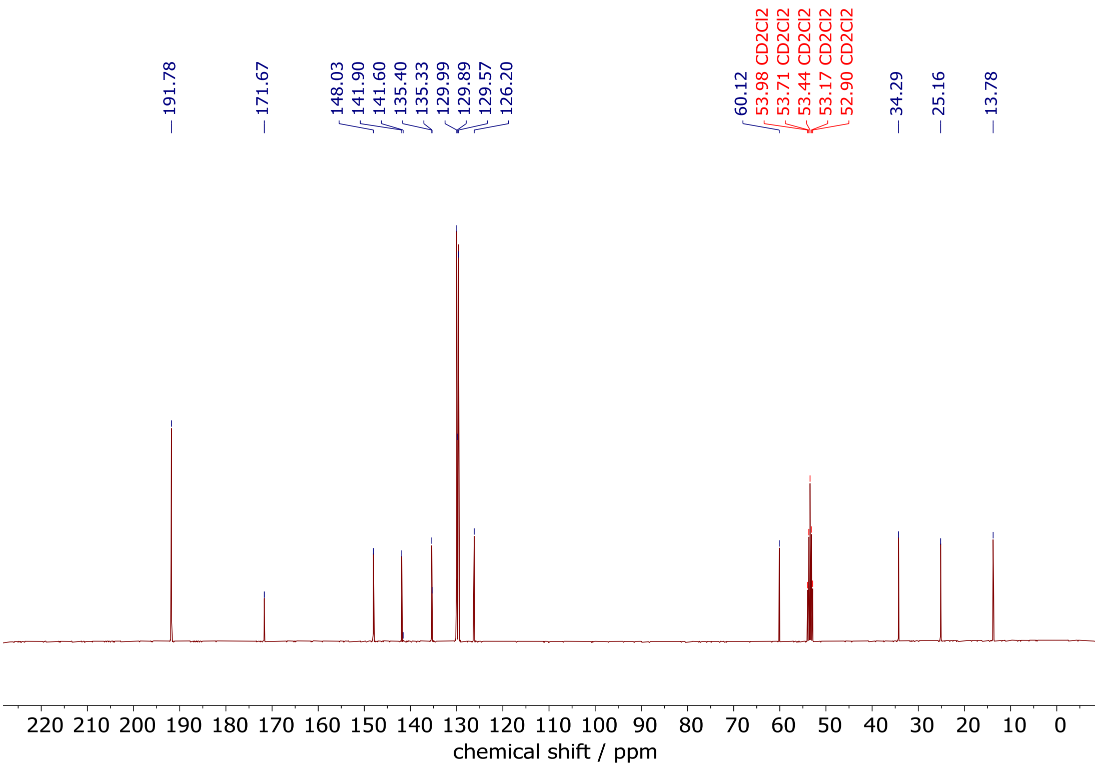

Figure S2. ^{13}C NMR Spectrum of **3** in CD_2Cl_2 at rt.

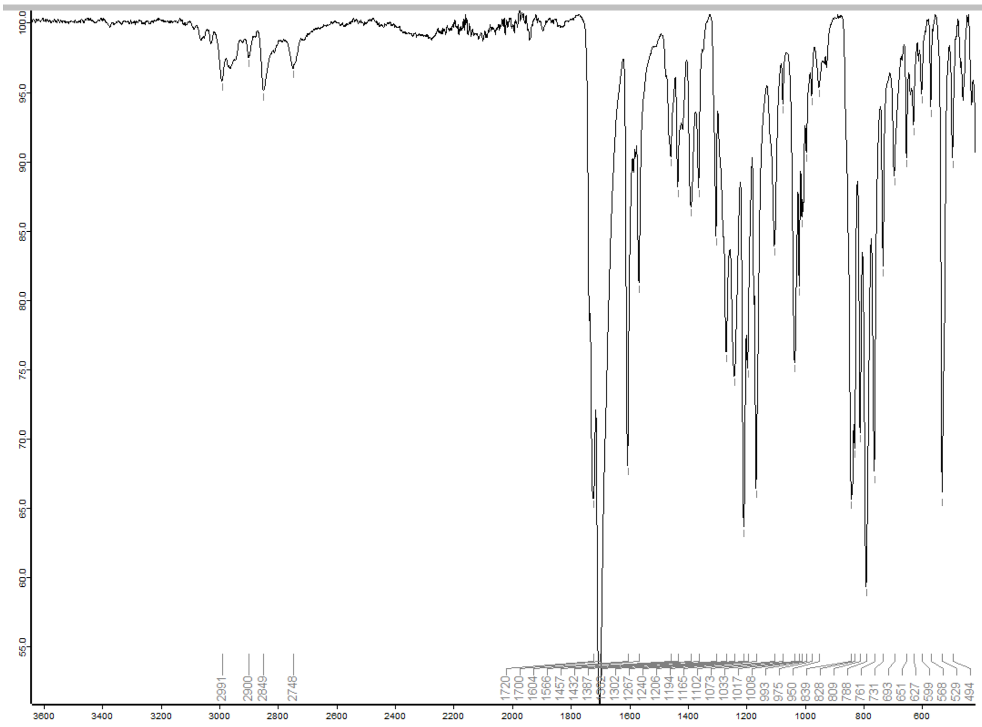

Figure S3. FTIR transmission spectrum of **3** at rt.

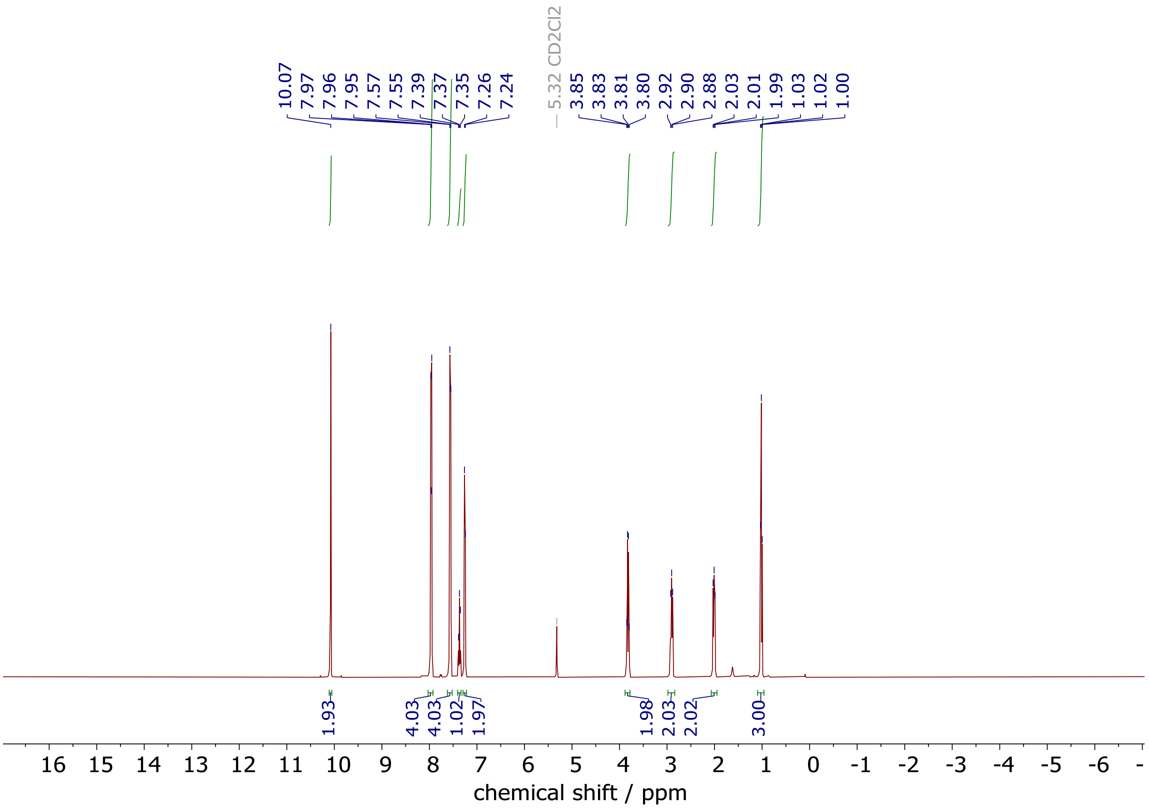

Figure S4. ^{1}H NMR Spectrum of **4** in CD_2Cl_2 at rt.

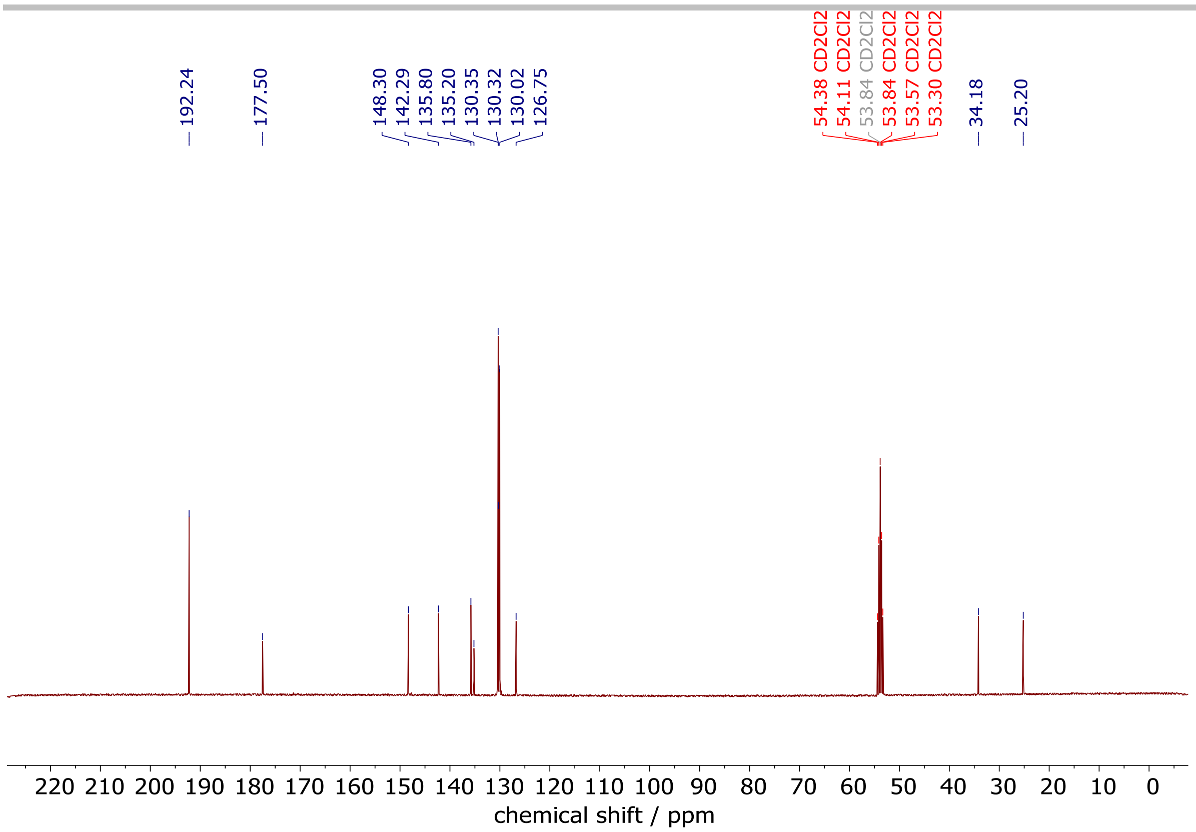

Figure S5. ^{13}C NMR Spectrum of **4** in CD_2Cl_2 at rt.

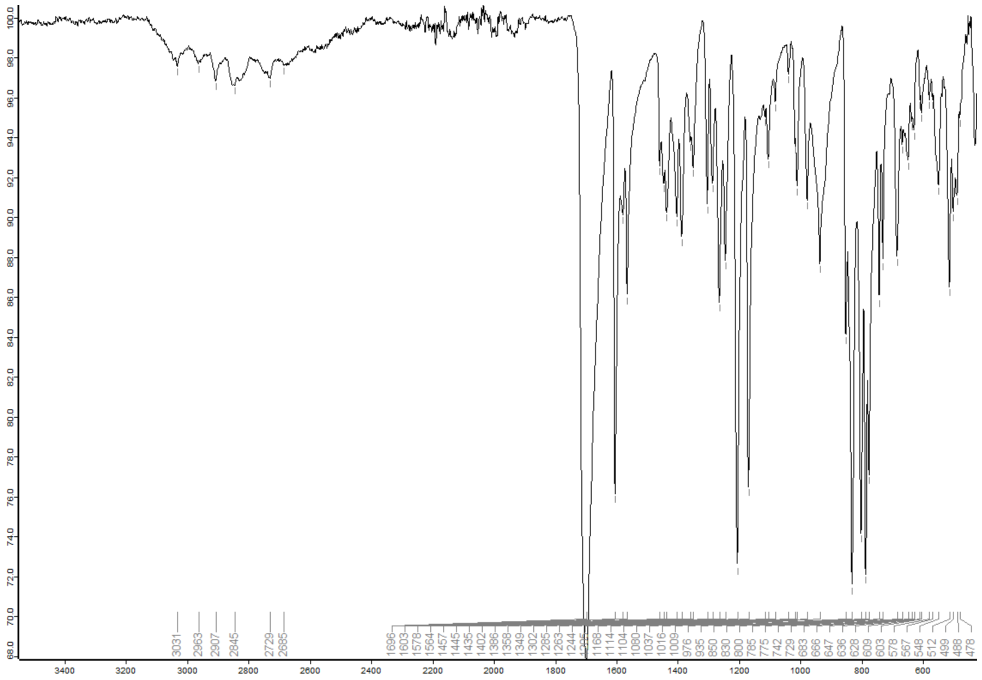

Figure S6. FTIR transmission spectrum of **3** at rt.

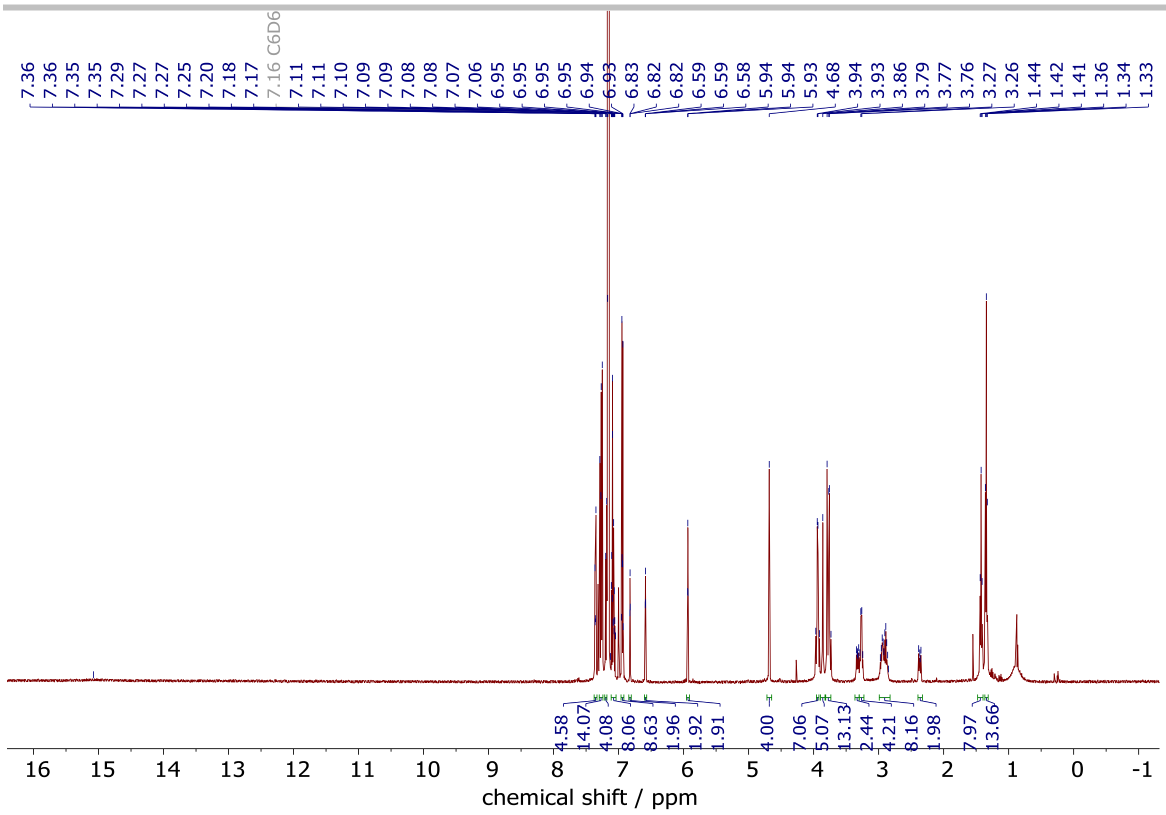

Figure S7. ^{1}H NMR Spectrum of **1** in C_6D_6 at rt.

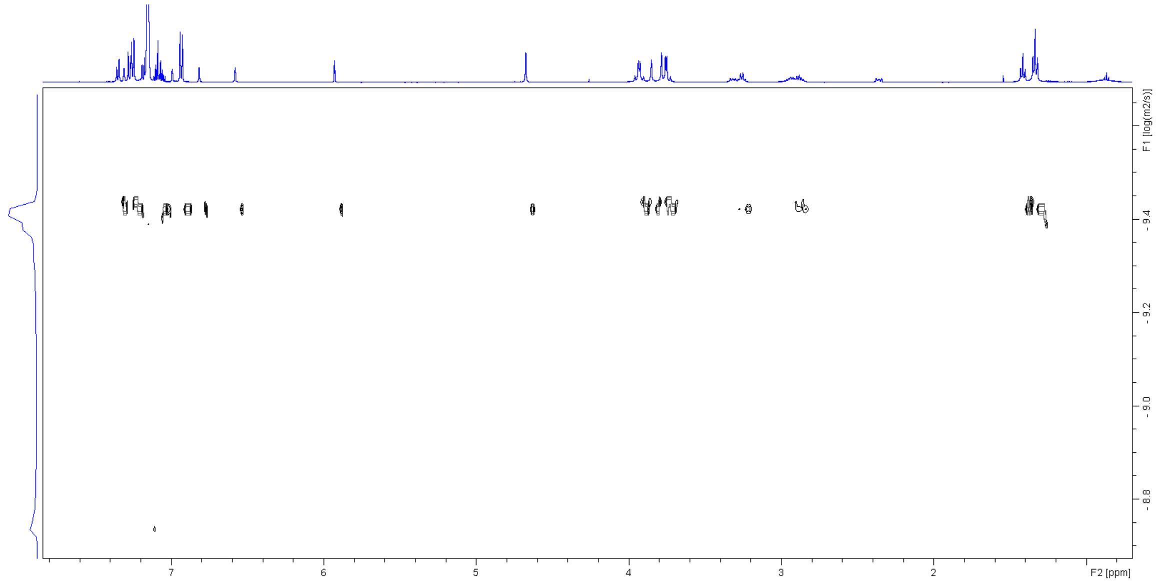

Figure S8. ^{1}H DOSY NMR Spectrum of **1** in C_6D_6 at rt.

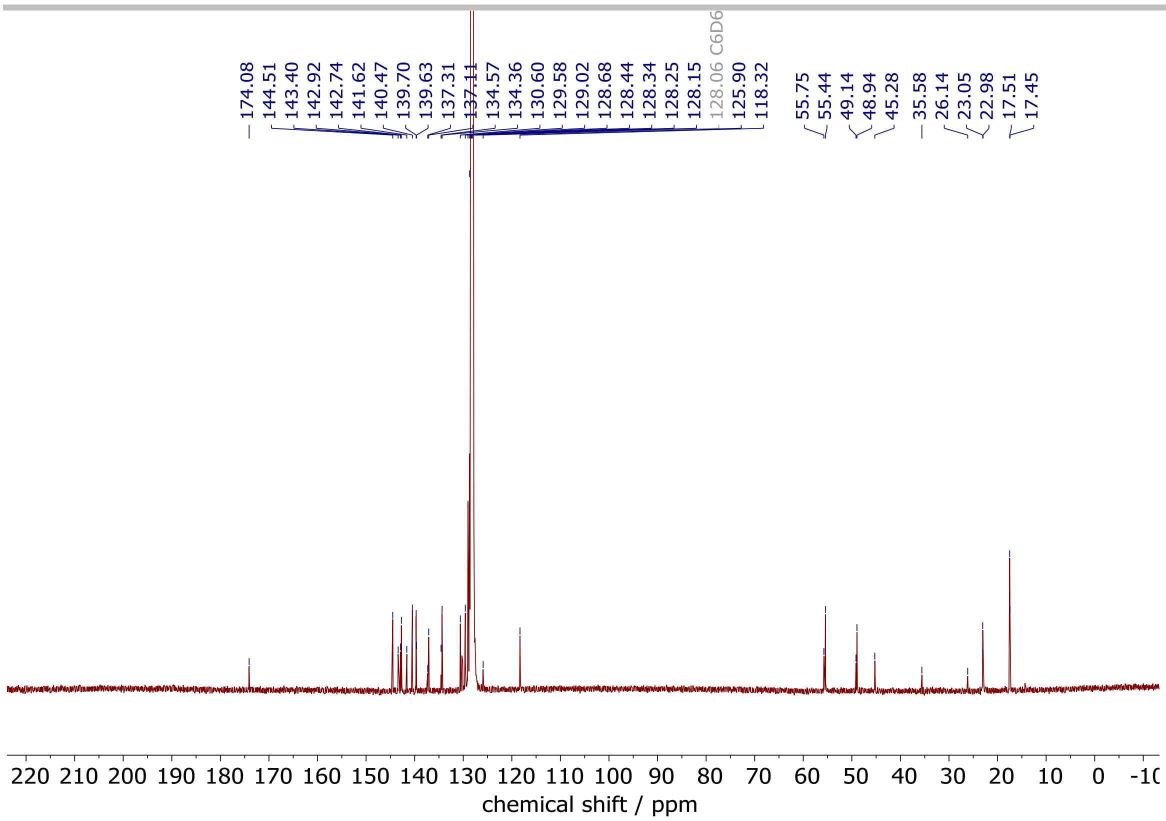

Figure S9. ^{13}C NMR Spectrum of **1** in C_6D_6 at rt.

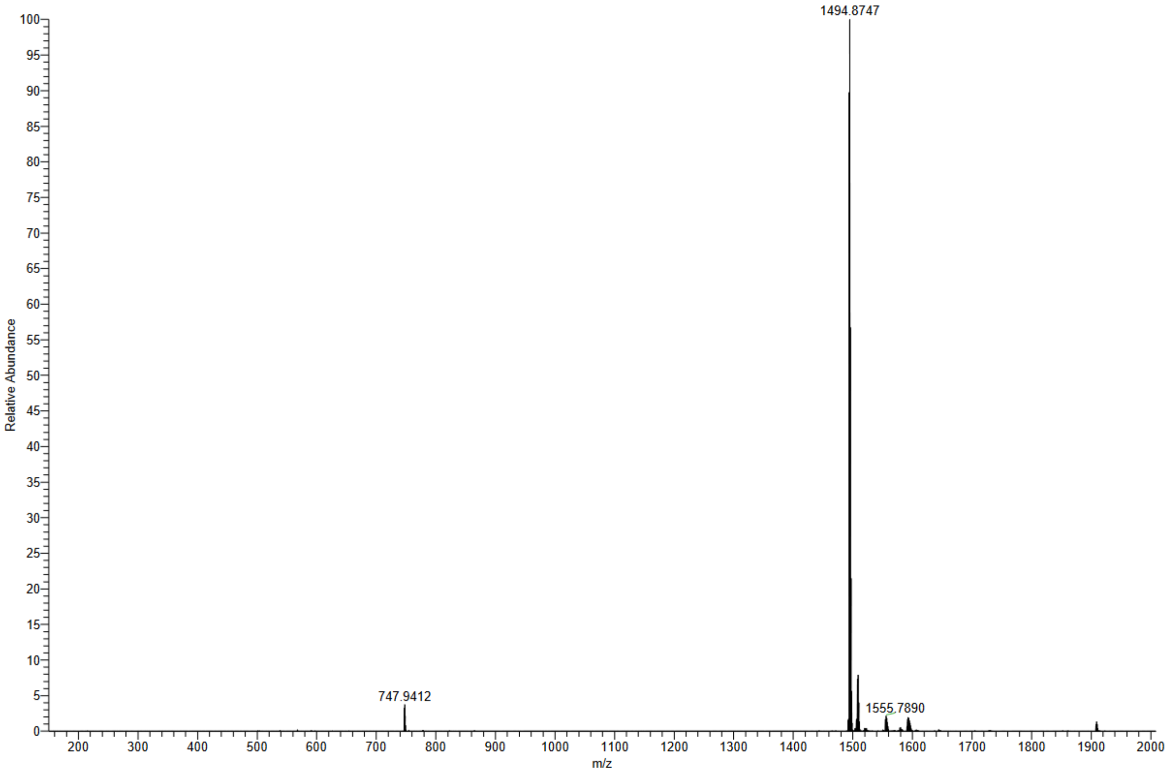

Figure S10. ESI-MS of **1**.

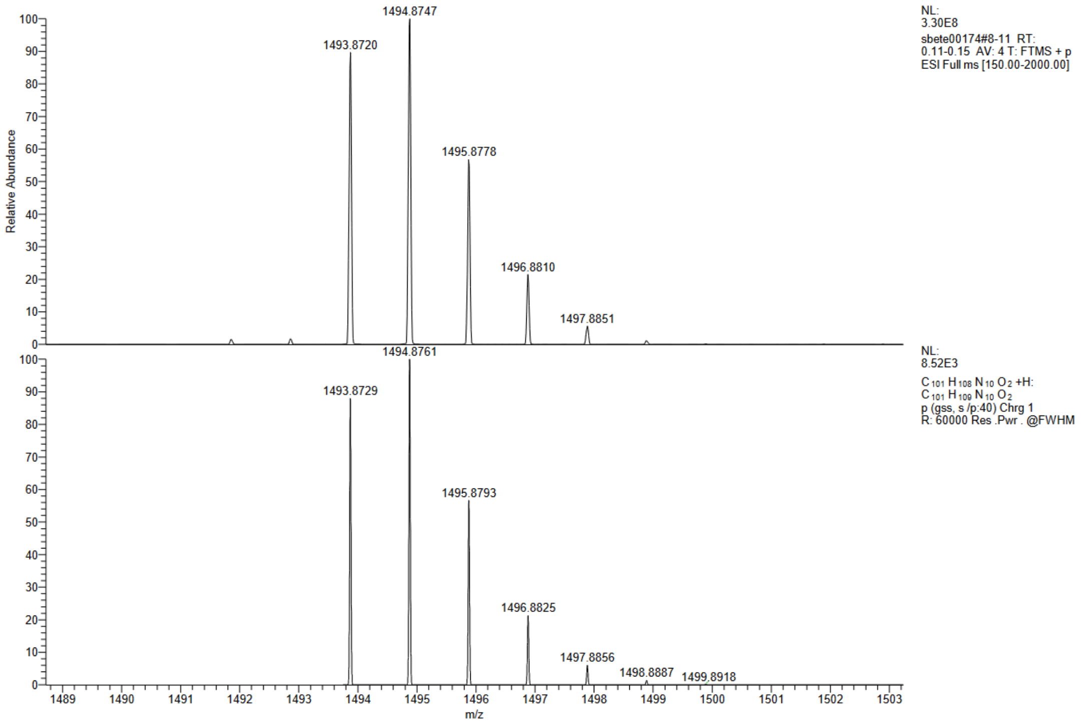

Figure S11. Measured and calculated ESI-MS of **1** for $[M+H]^{+}$.

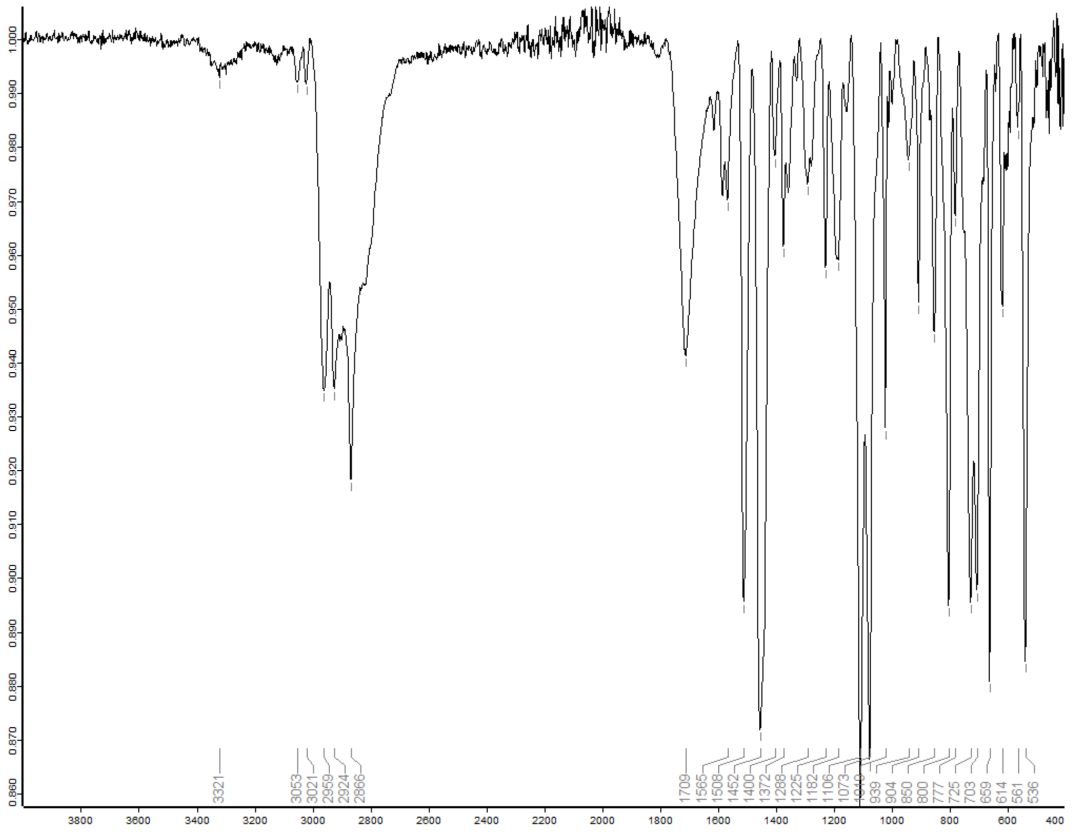

Figure S12. FTIR transmission spectrum of **1** at rt.

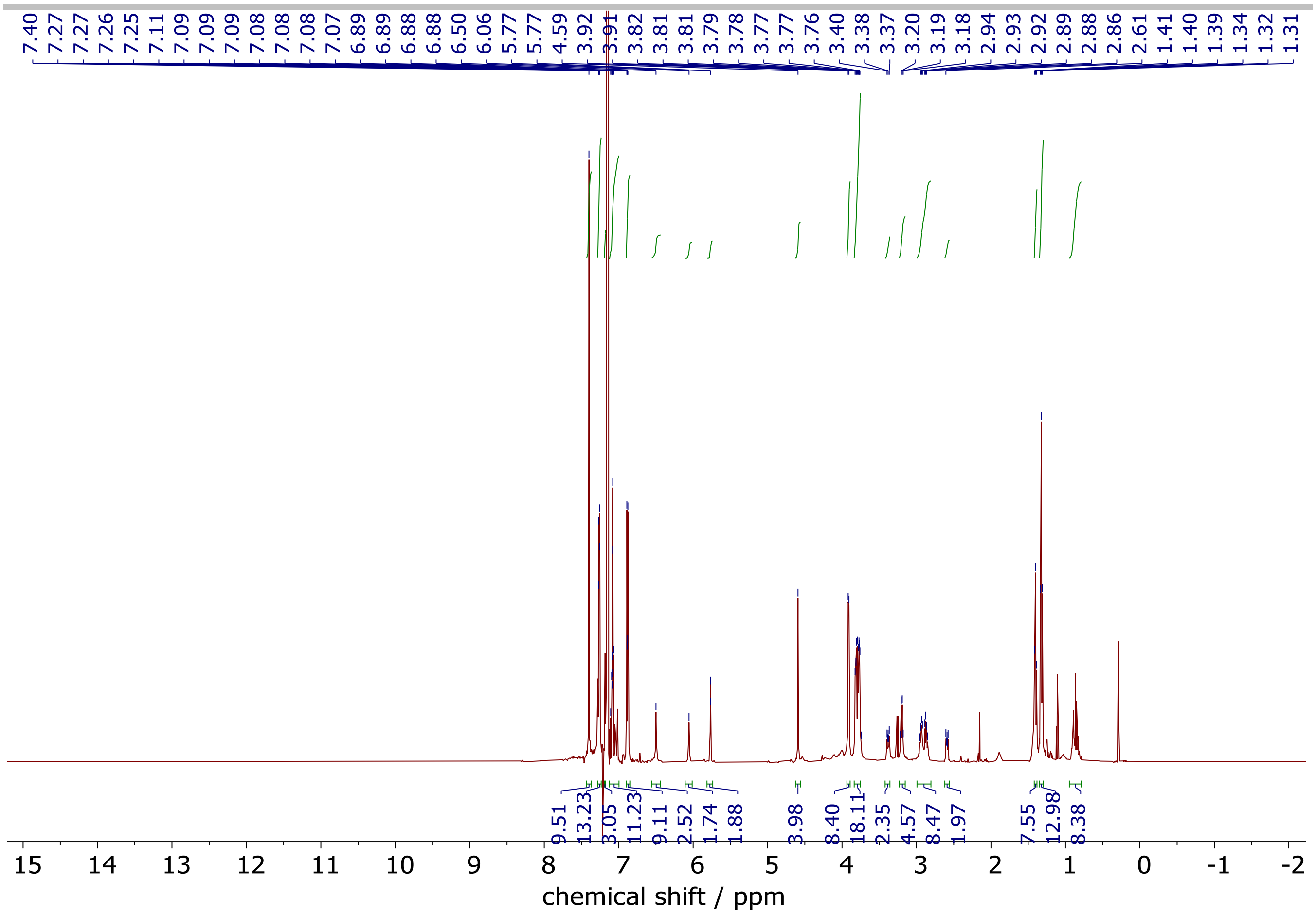

Figure S13. ^{1}H NMR Spectrum of **1-Cu** in C_6D_6 at rt.

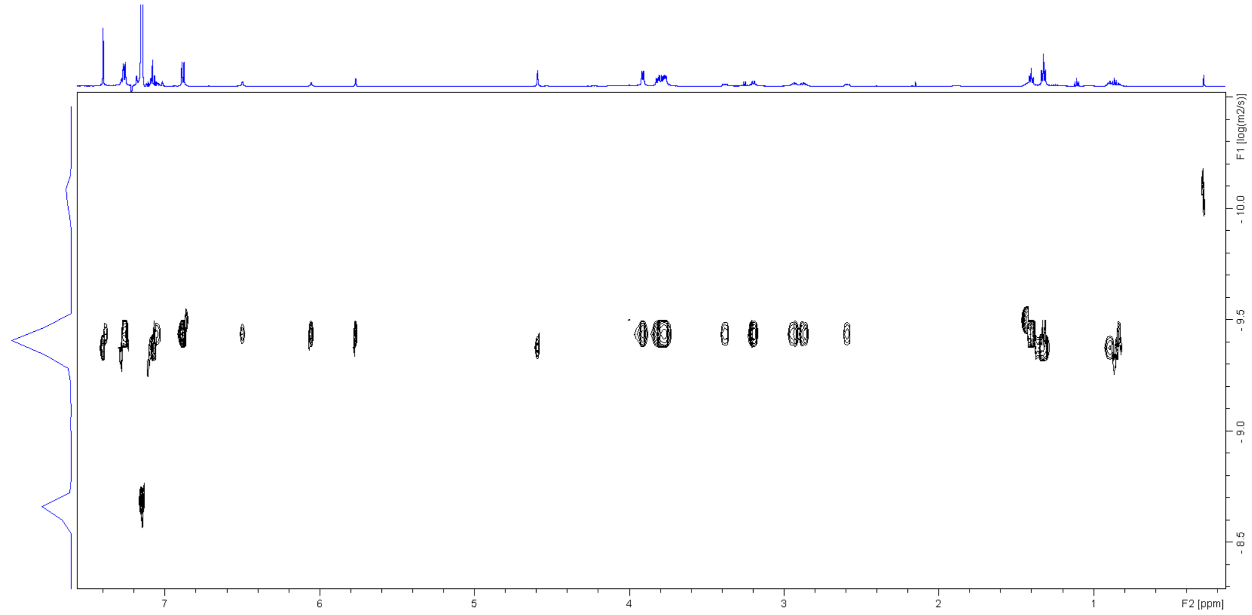

Figure S14. ^{1}H DOSY NMR Spectrum of **1-Cu** in C_6D_6 at rt.

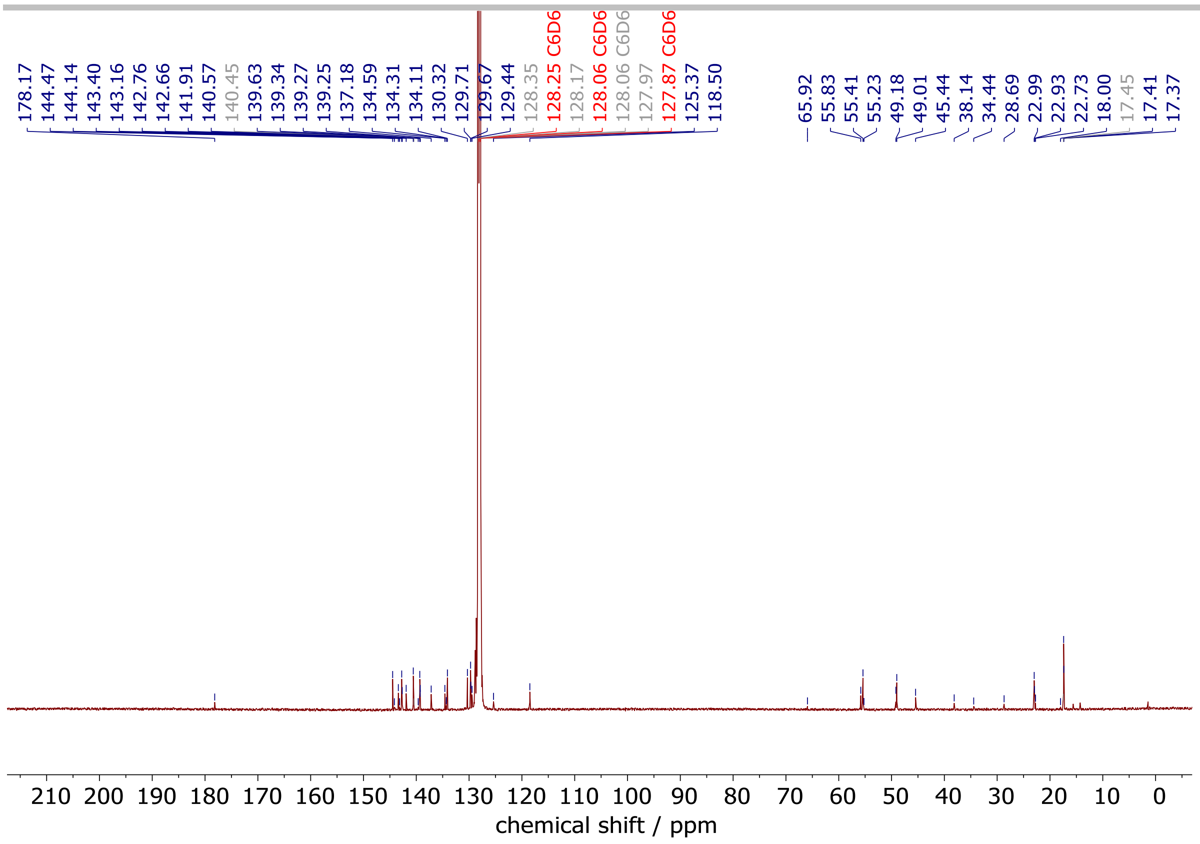

Figure S15. ^{13}C NMR Spectrum of **1-Cu** in C_6D_6 at rt.

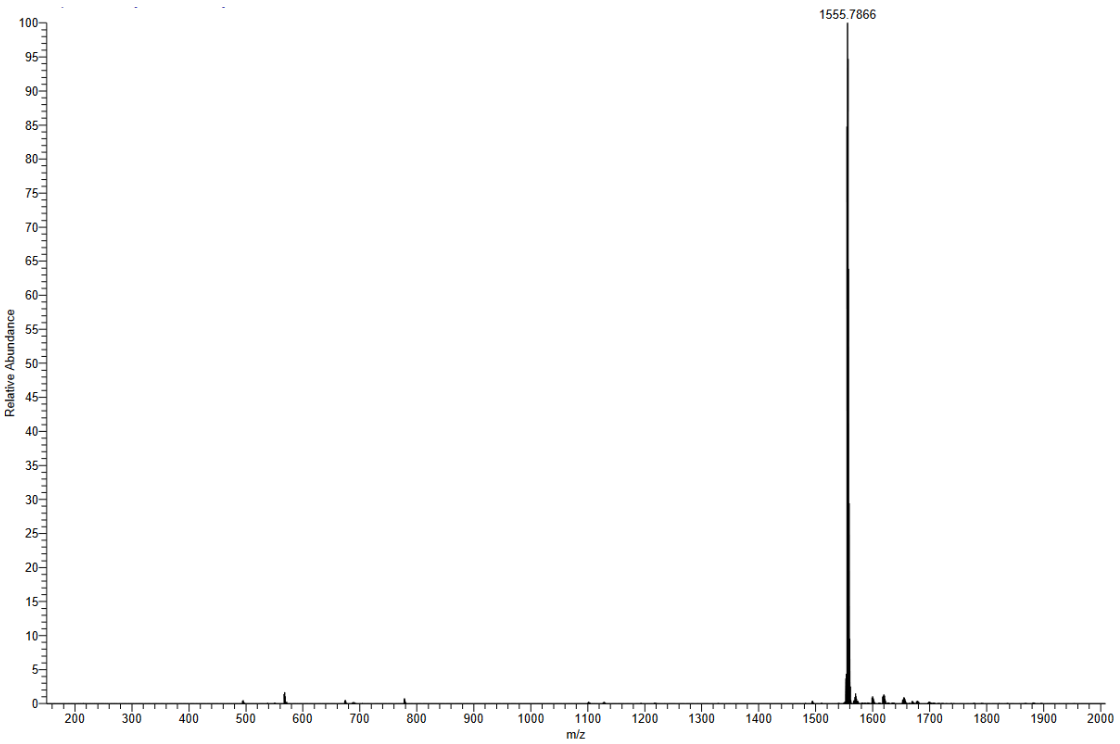

Figure S16. ESI-MS of **1-Cu**.

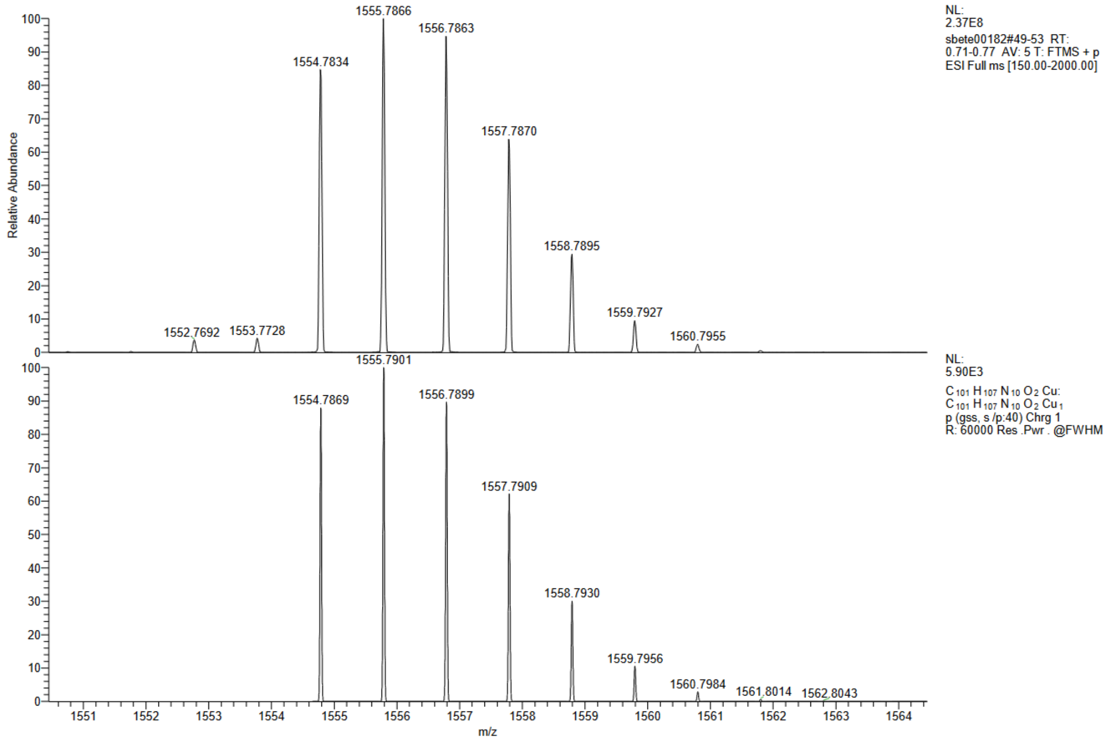

Figure S17. Measured and calculated ESI-MS of **1-Cu** for [M-e]$^+$.

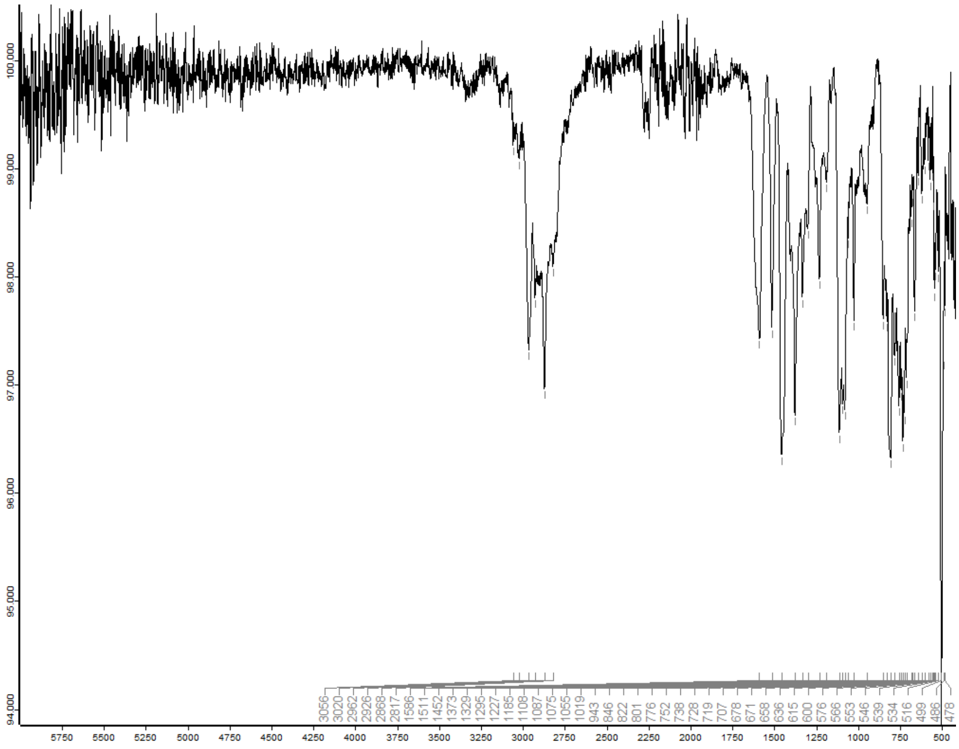

Figure S18. FTIR transmission spectrum of **1-Cu** at rt.

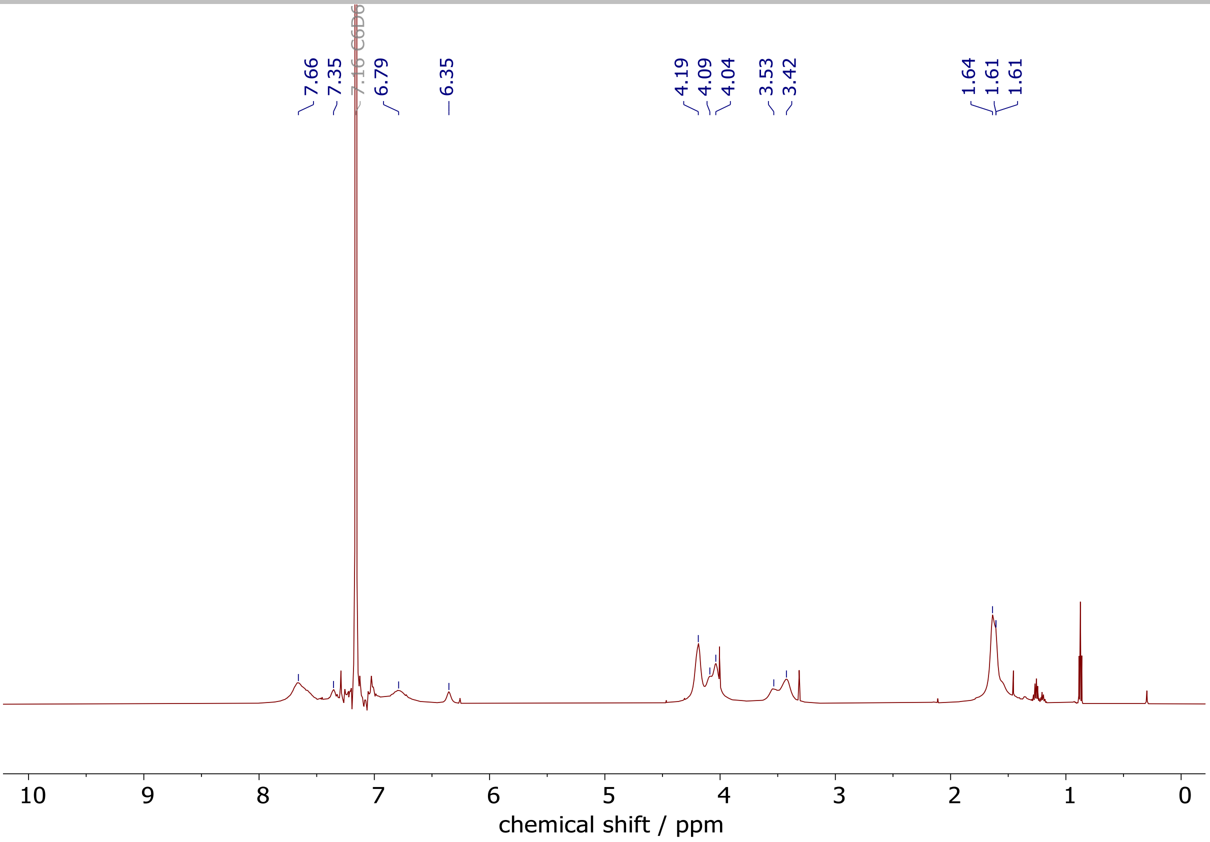

Figure S19. Diamagnetic region of the ^{1}H NMR Spectrum of [**1-Cu]PF₆** in C_6D_6 at rt.

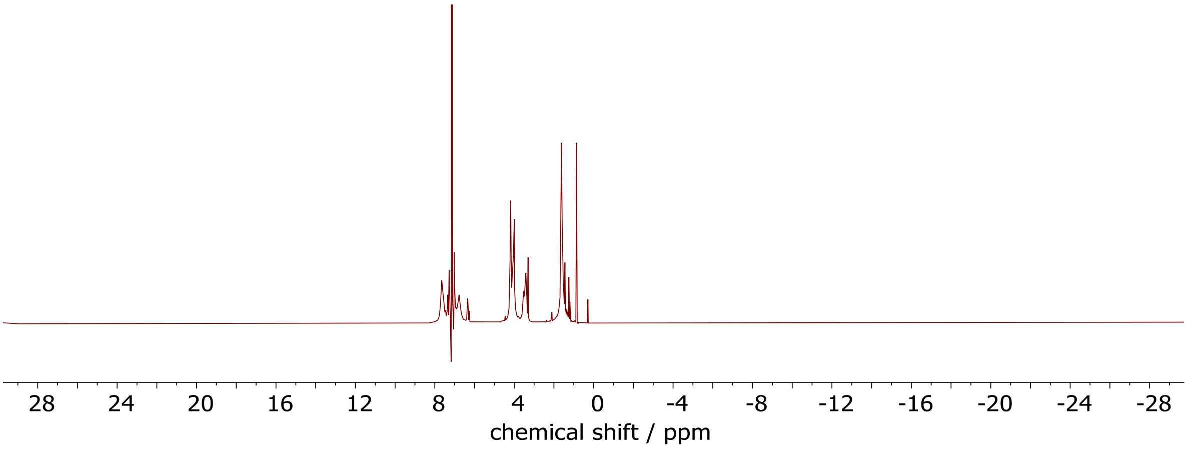

Figure S20. ^{1}H NMR Spectrum of [**1-Cu]PF$_6$** in C_6D_6 at rt.

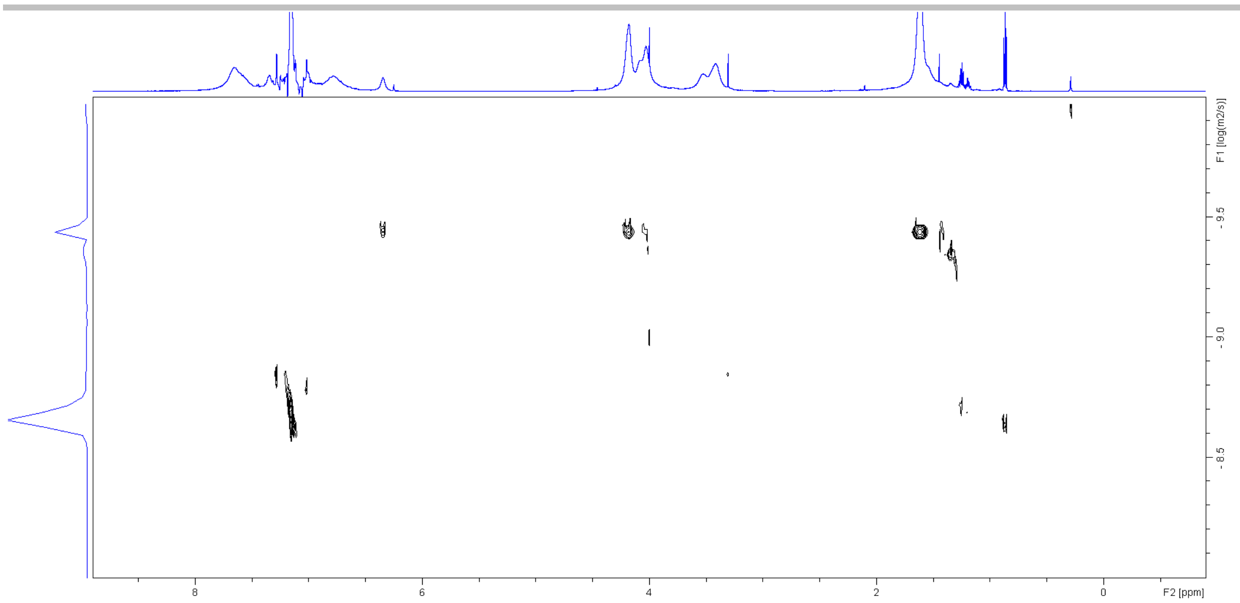

Figure S21. ^{1}H DOSY NMR Spectrum of [**1-Cu]PF6** in C_6D_6 at rt.

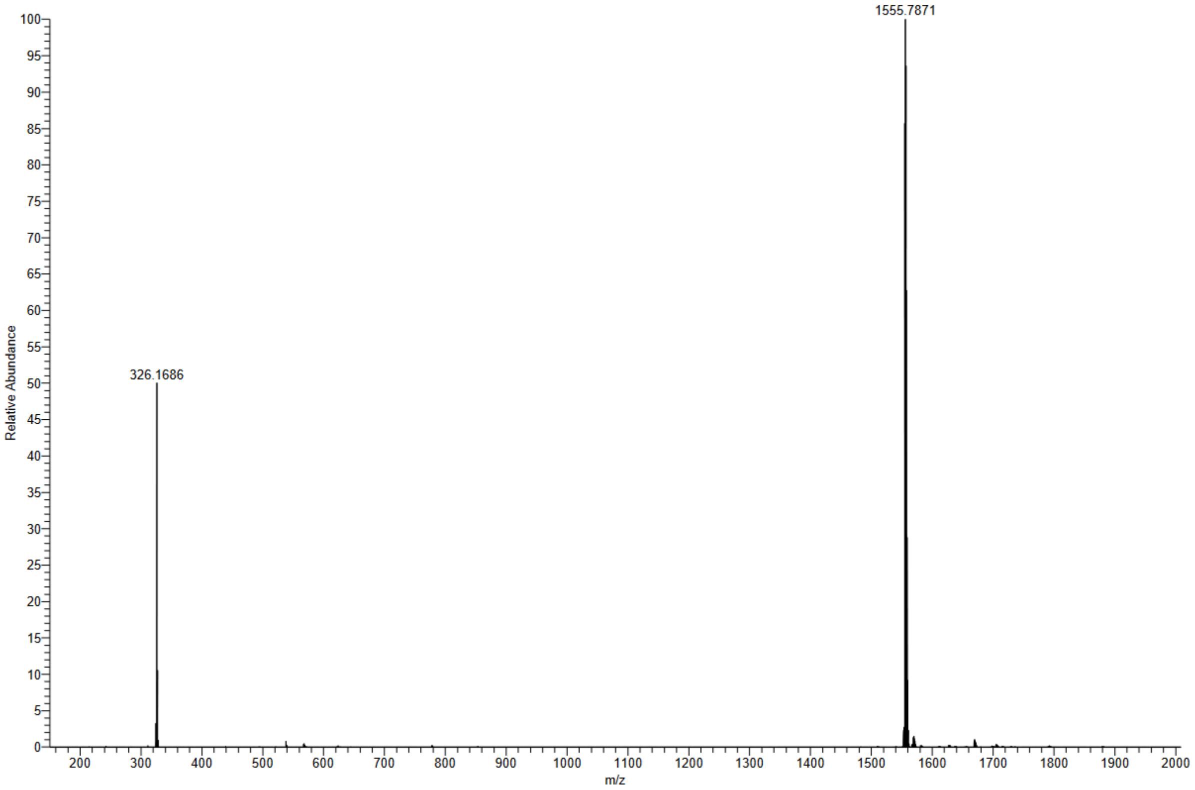

Figure S22. ESI-MS of **[1-Cu]PF6**.

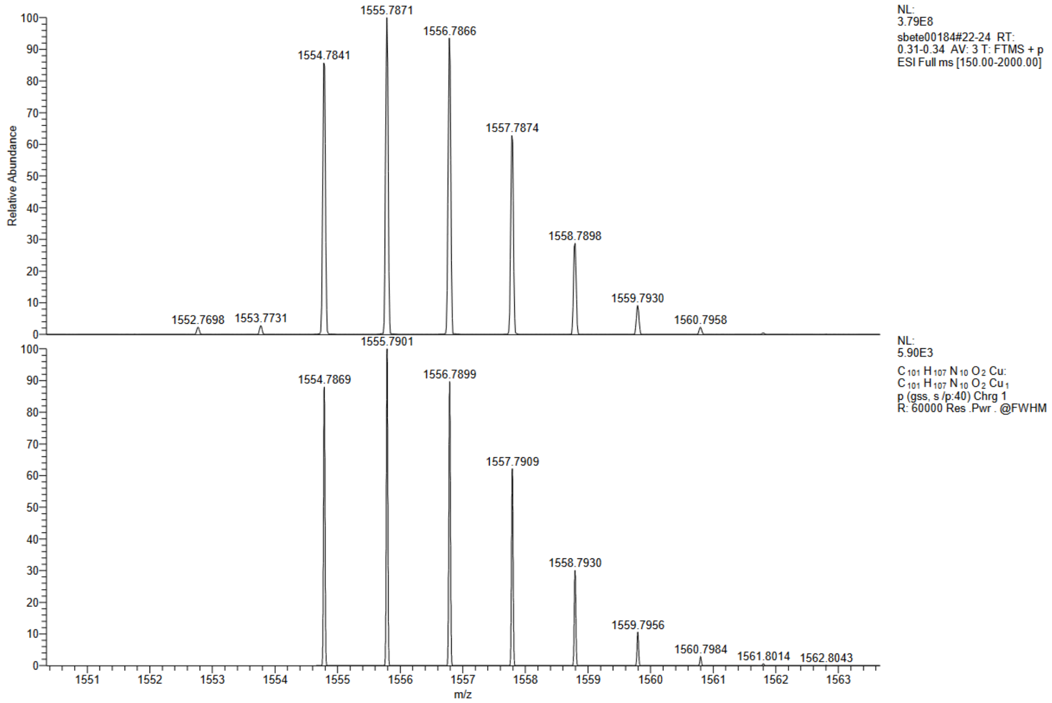

Figure S23. Measured and calculated ESI-MS of [1-Cu]PF_6 for $[M]^+$.

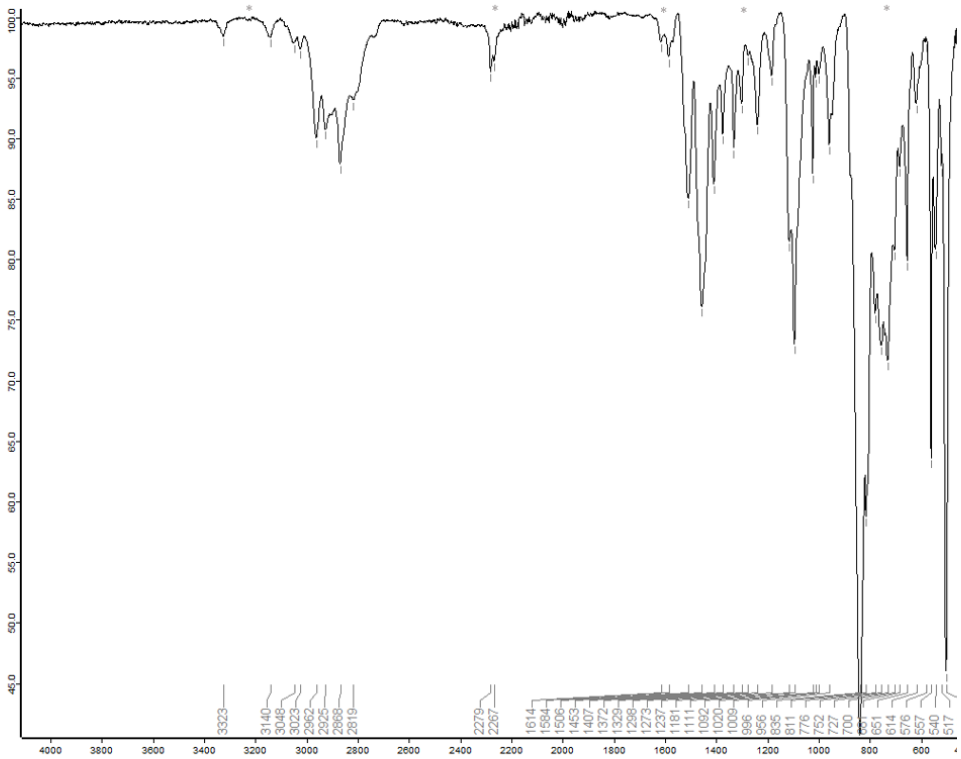

Figure S24. FTIR transmission spectrum of **[1-Cu]PF_6** at rt. Asterisks remark residual C_6D_6 signals, direct evaporation of the solvent significantly enhances signal intensity.

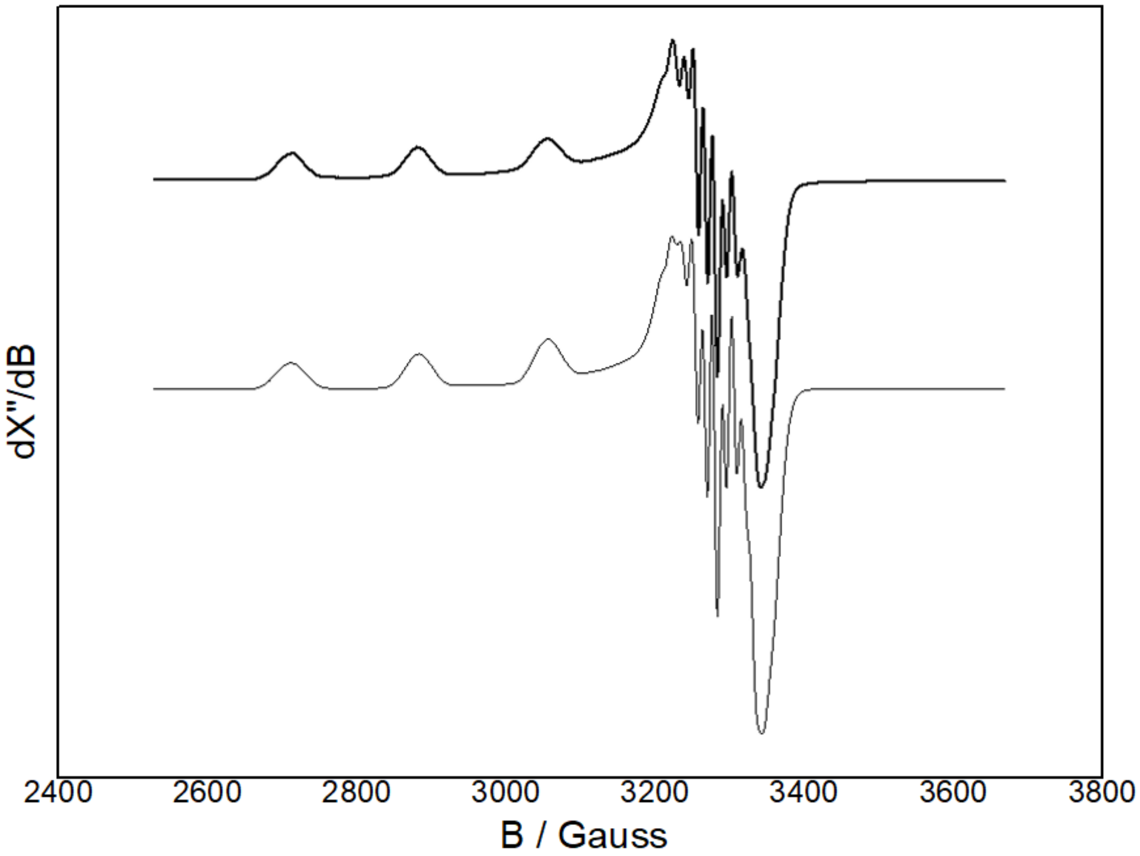

Figure 25. CW X-band EPR spectra (bold) and simulations (light) of **[1-Cu]PF$_6$** in toluene at 147 K and 9.43 GHz.

Simulation data: S = 1/2; Nucs = 'Cu,N,N'; g values = [2.05587 2.05742 2.26716]; gStrain = [0.00460769, 0.0233163, 0.0183456]; A tensors = [60.6739, 23.1466, 532.521; 33.1719, 38.3373, 32.5408; 37.8525, 45.6205, 37.3442]).

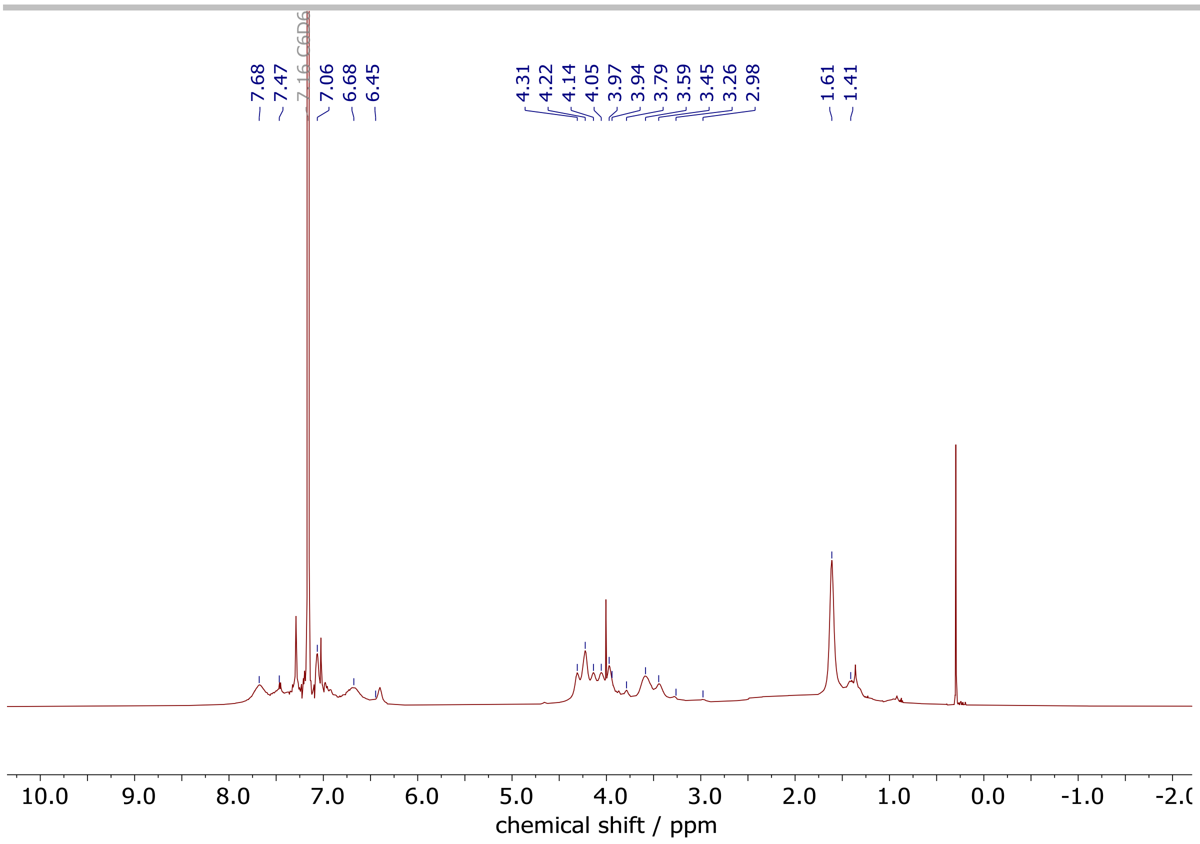

Figure S26. ^{1}H NMR Spectrum of [**1-Cu]PF$_6$** in the presence of water in C_6D_6 at rt.

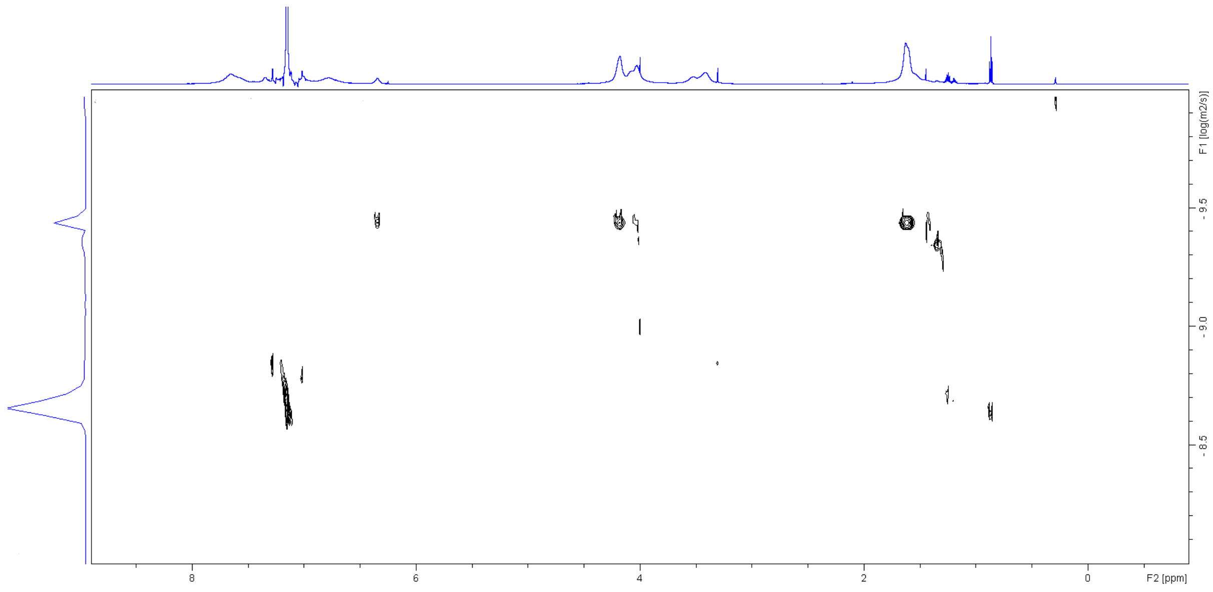

Figure S27. ^{1}H DOSY NMR Spectrum of [**1-Cu]PF$_6$** in the presence of water in C_6D_6 at rt.

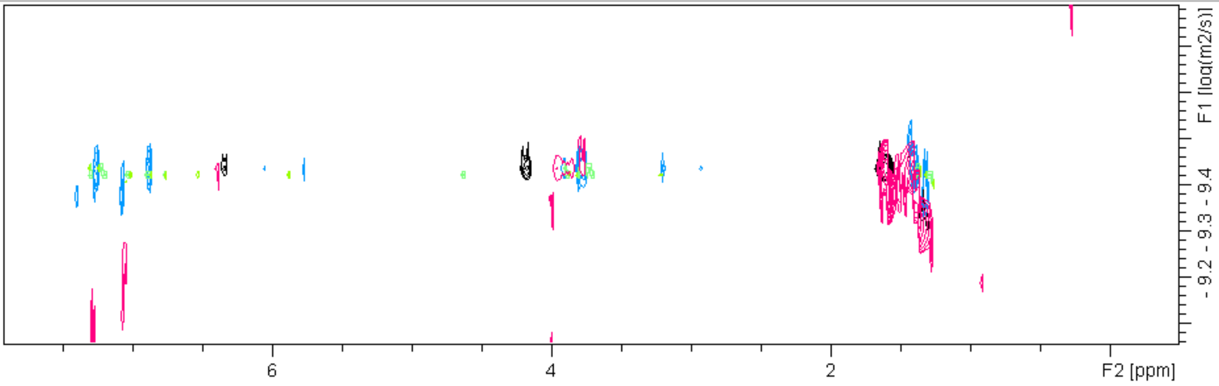

Figure S28. Stacked ^{1}H DOSY NMR Spectra of **1** (green), **1-Cu** (blue), [**1-Cu]PF$_6$** in absence (black) and in the presence of water (pink) in C_6D_6 at rt.

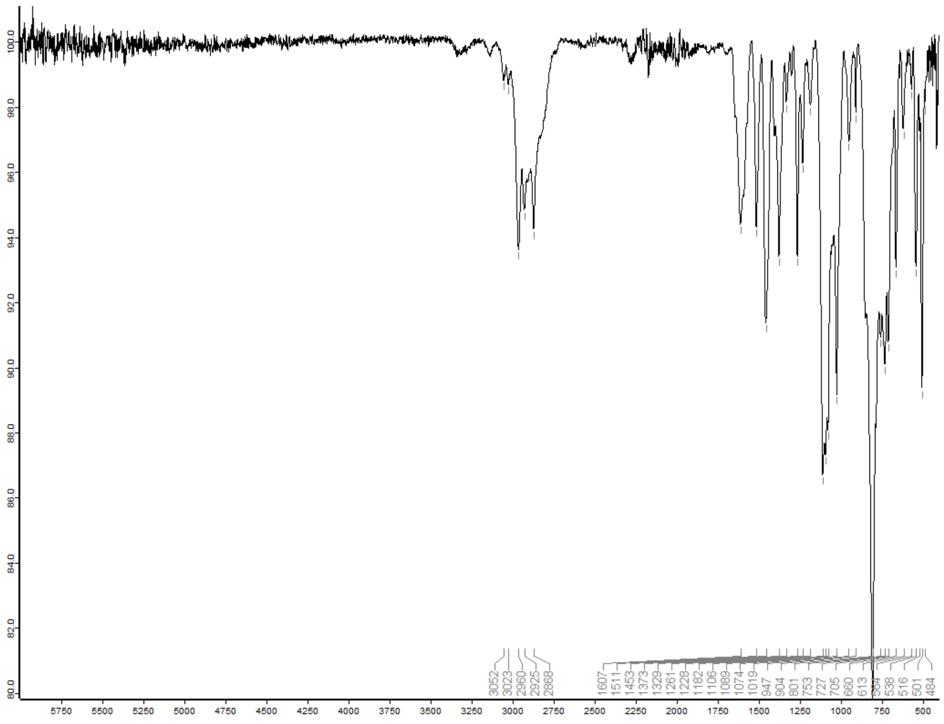

Figure S29. FTIR transmission spectrum of **1-CuPF$_6$** in the presence of water in C_6D_6 at rt.

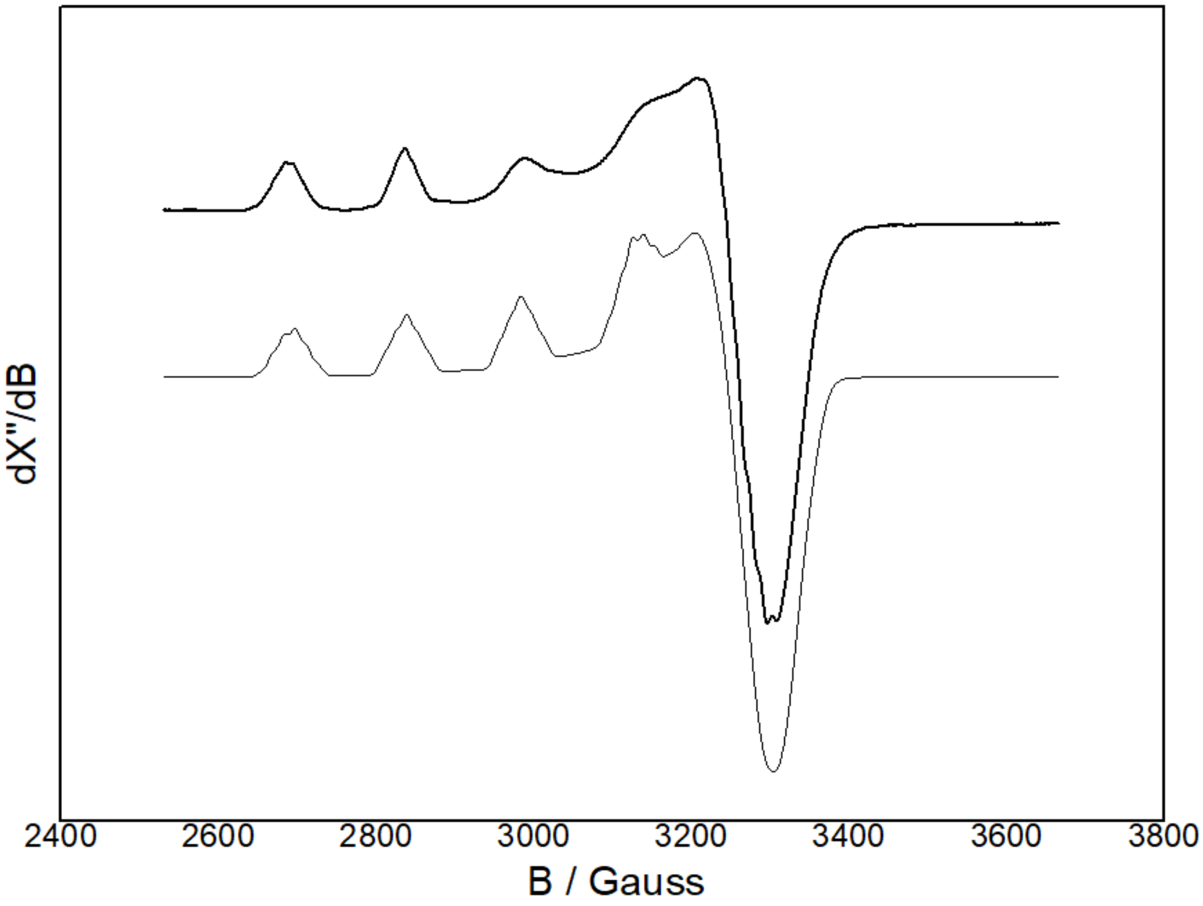

Figure S30. CW X-band EPR spectra (bold) and simulations (light) of **[1-Cu]PF$_6$** in the presence of water in toluene at 147 K and 9.43 GHz.

Simulation data: S = 1/2; Nucs = 'Cu,N,N'; g values = [2.05486 2.084331 2.3131]; gStrain = [0.0165 0.0467 0.0114]; A tensors = [7.9812 3.8669 461.1190; 49.1699 66.6259 43.7617; 63.3519 58.0004 47.4570]).

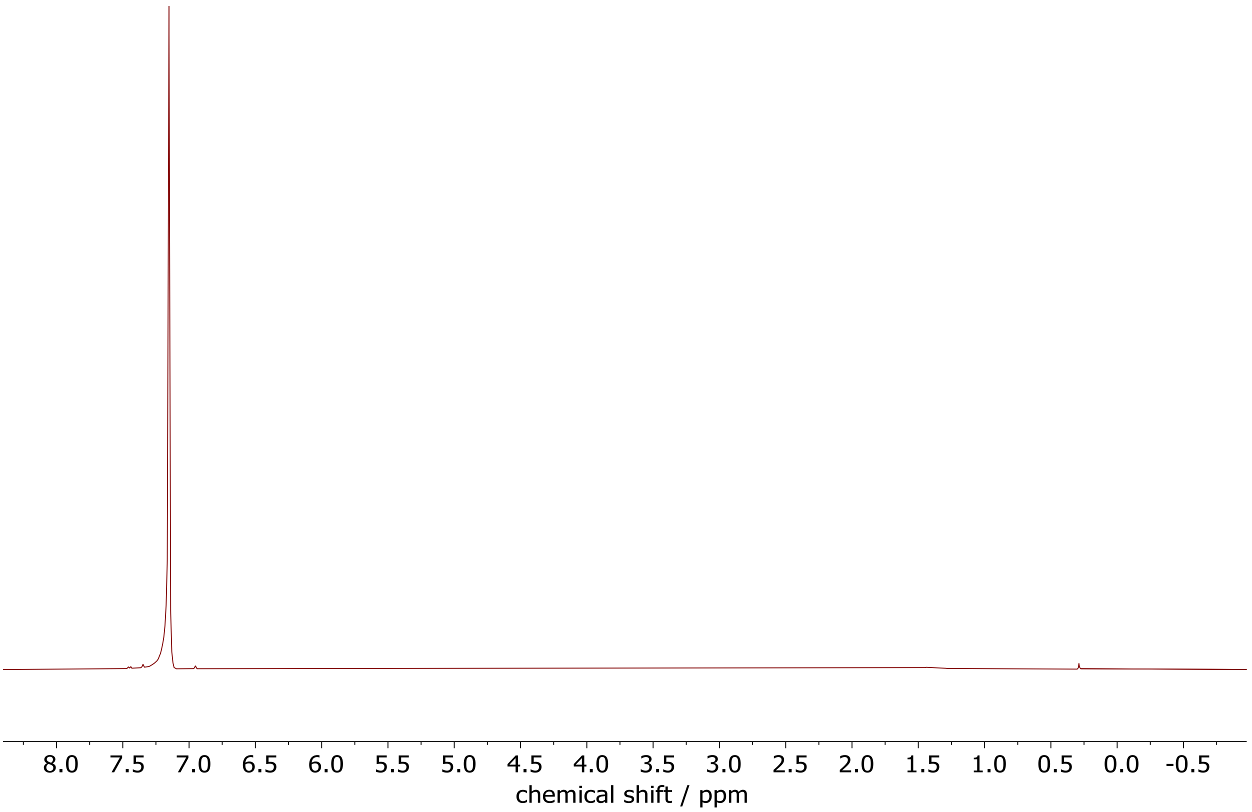

Figure S31. ^{1}H NMR spectrum of **1-Cu** after oxygen exposure.

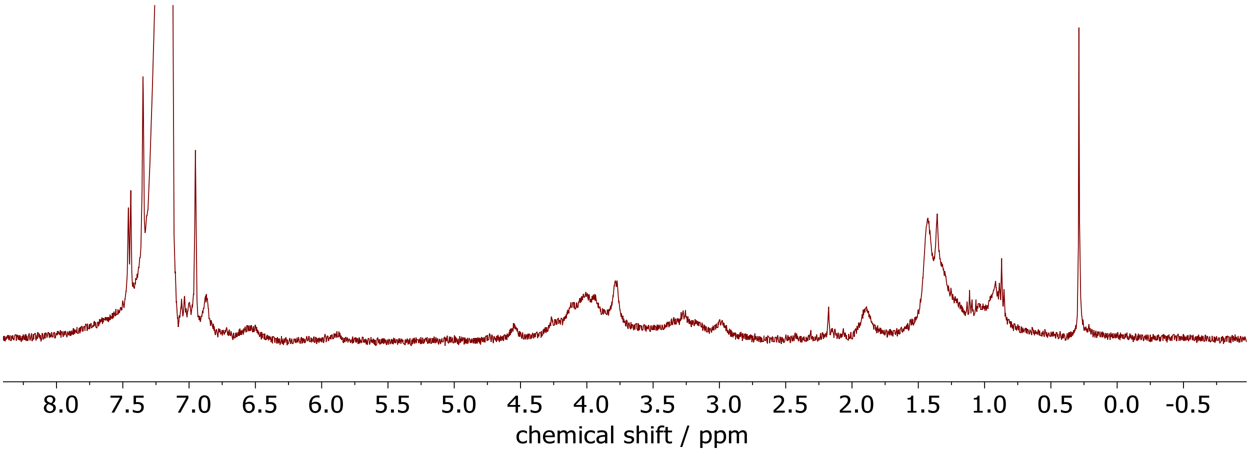

Figure S32. ^{1}H NMR spectrum of **1-Cu** after oxygen exposure.

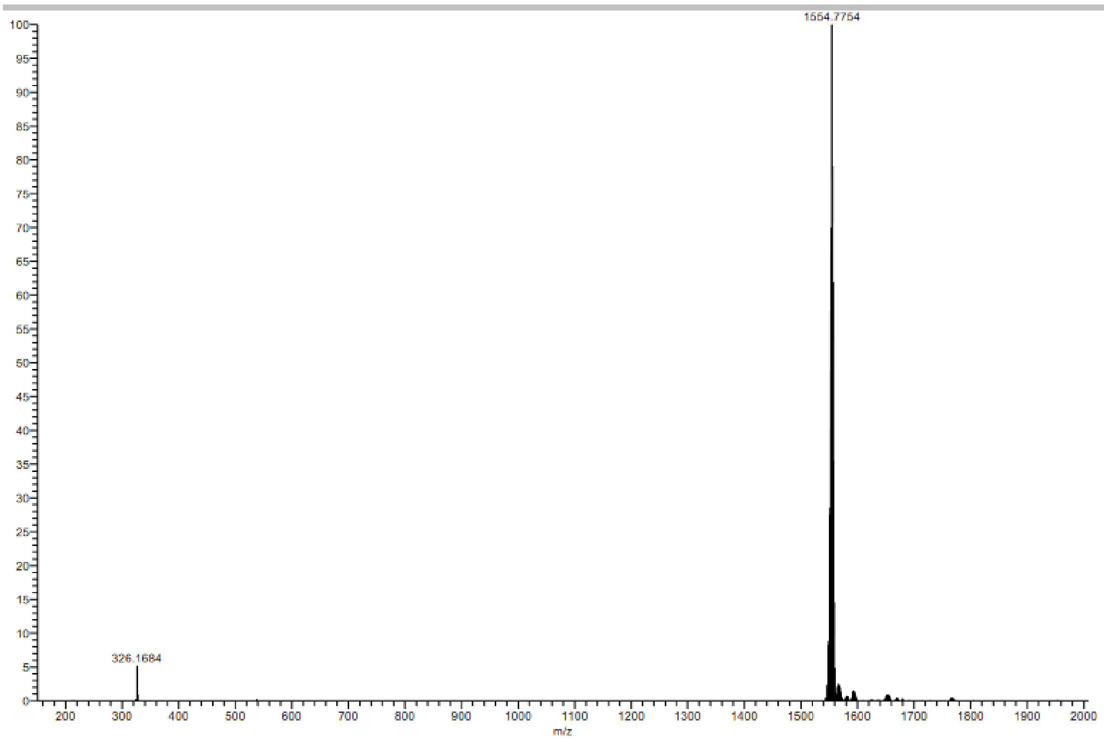

Figure S33. ESI-MS of **1-Cu** after oxygen exposure.

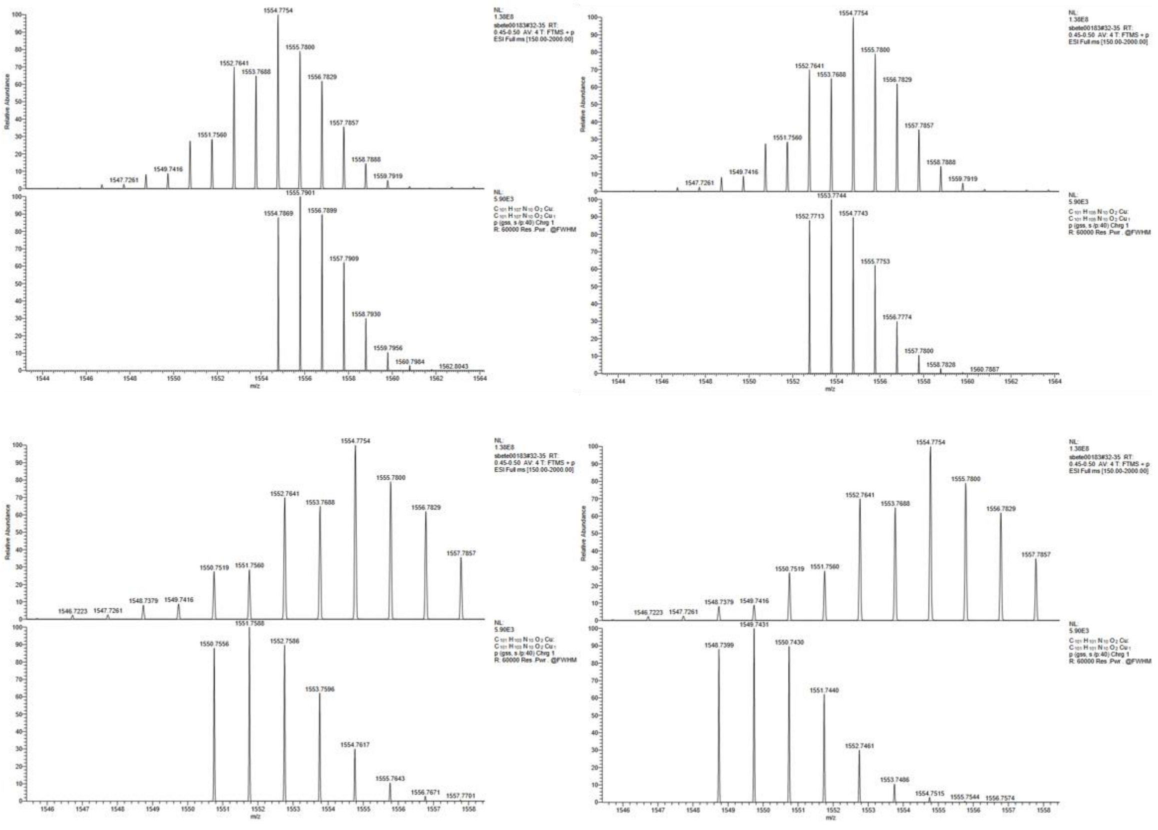

Figure S34. Measured and calculated ESI-MS of **1-Cu** after oxygen exposure for [M-e]$^+$ (top left), [M-e-2H]$^+$ (top right), [M-e-4H]$^+$ (bottom left) and [M-e-6H]$^+$ (bottom right).

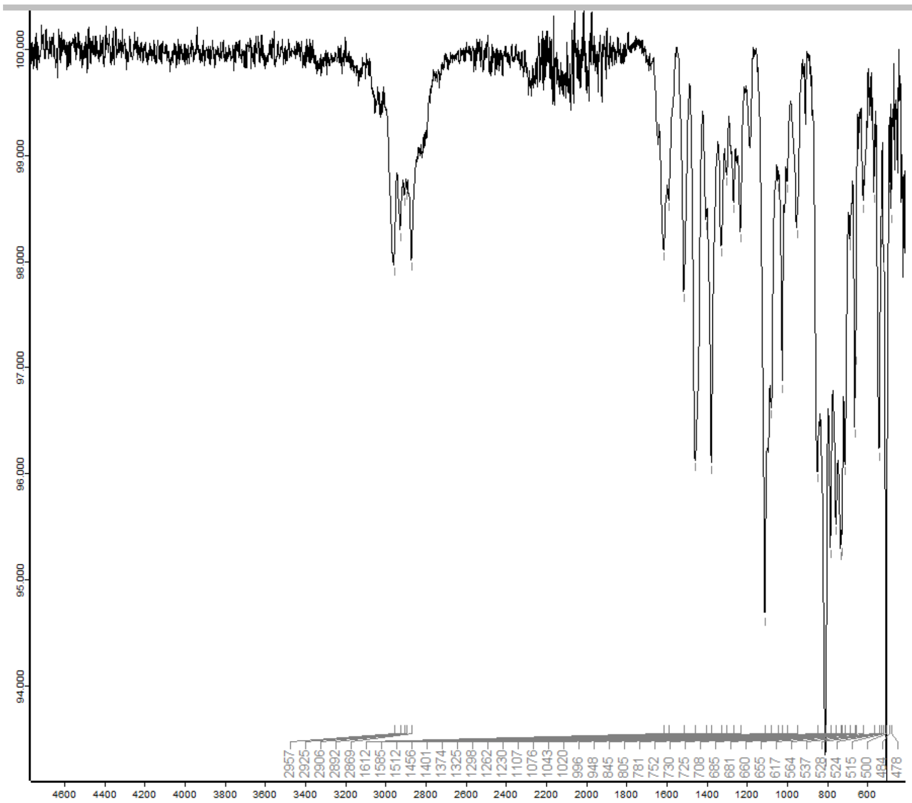

Figure S35. FTIR transmission spectrum of **1-Cu** after oxygen exposure.

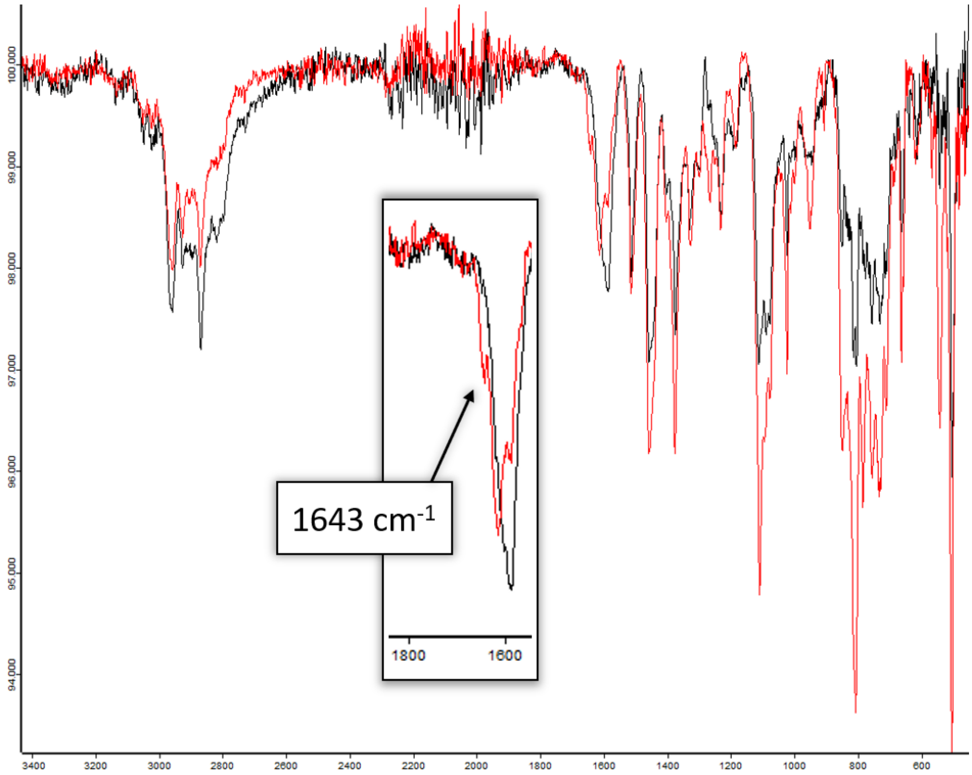

Figure S36. FTIR transmission spectrum of **1-Cu** (black) and after oxygen exposure (red).

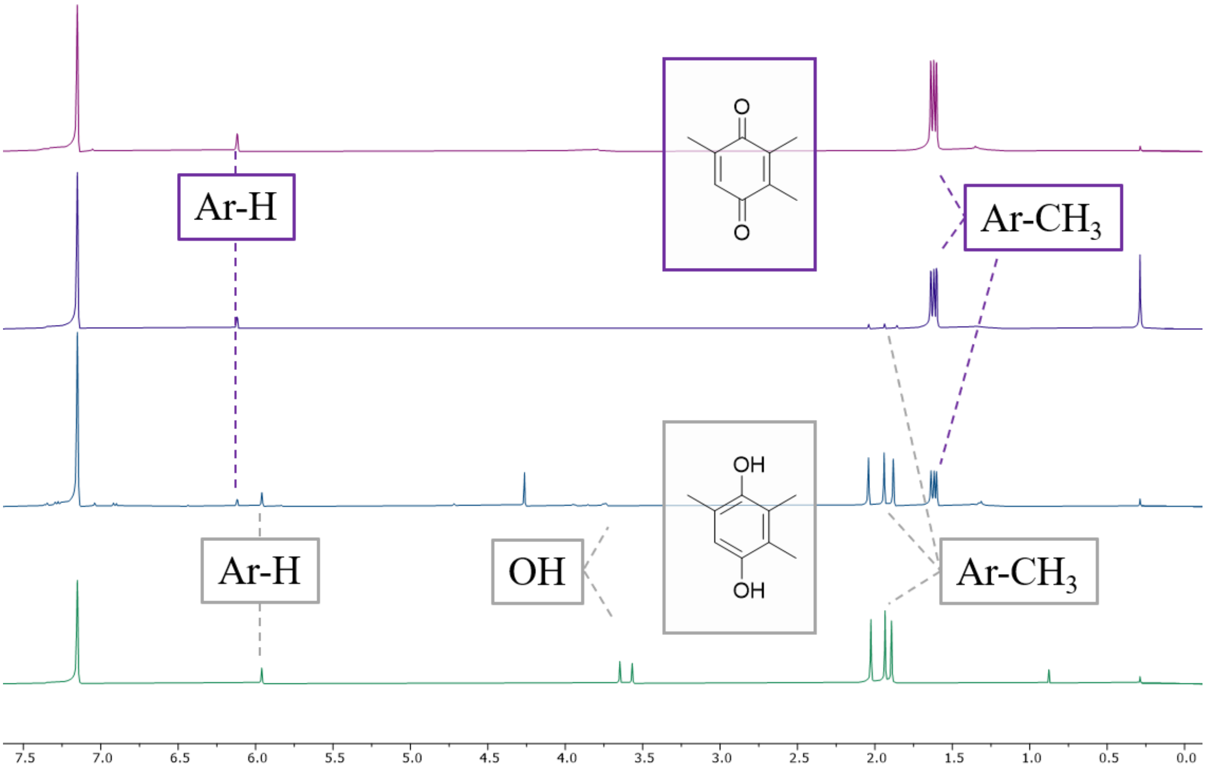

Figure S37. ^{1}H NMR spectra of the reaction of TMHQ with air at -20 °C, in presence of **1-Cu** (pink), **[1-Cu]PF$_6$** (purple), **1** (blue) and Cu(MeCN)$_4$PF$_6$ (green), spectra in C_6D_6 at rt.

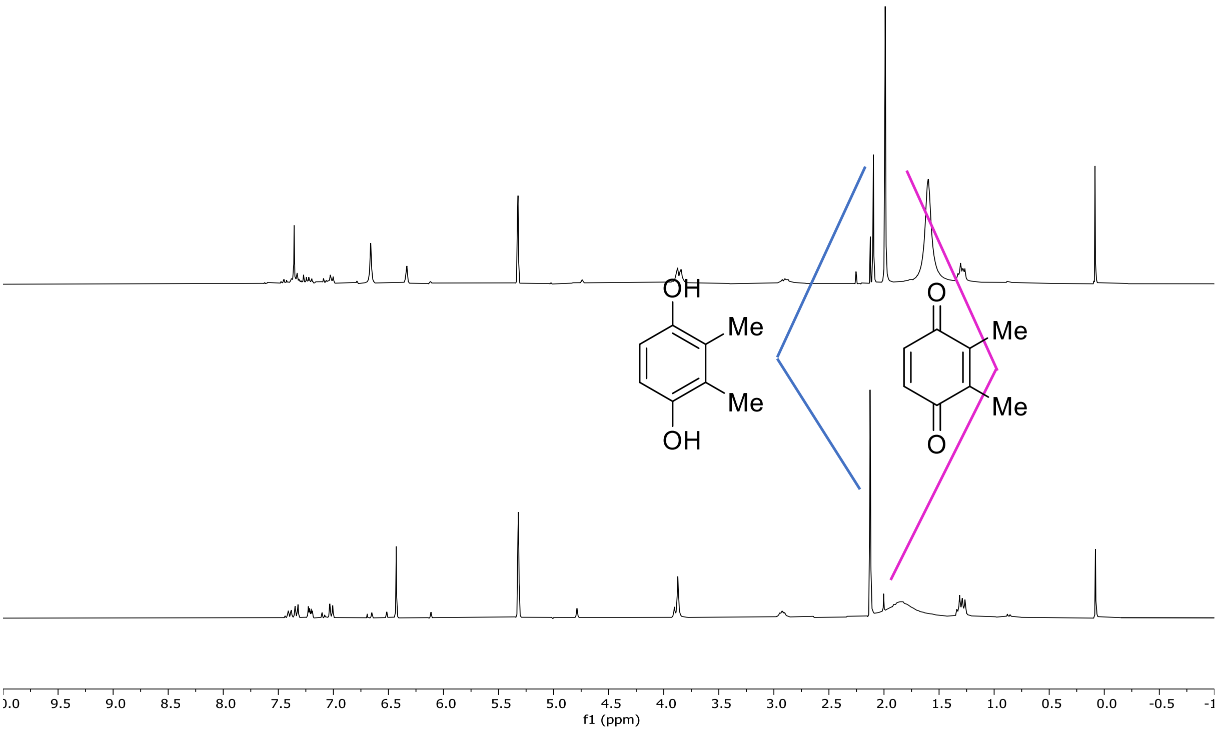

Figure S38. ^{1}H NMR spectra of the reaction of 2,3-dimethylhydroquinone with air at -20 °C, in presence of **1-Cu** (top) and **1** (bottom), spectra in CD_2Cl_2 at rt.

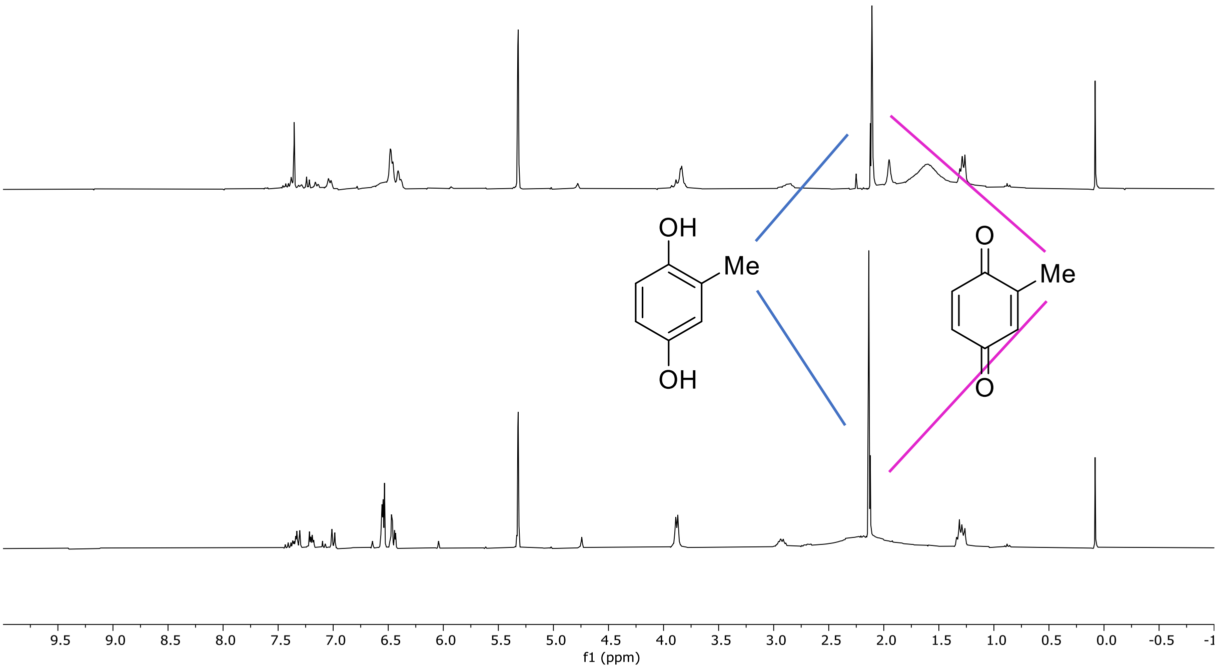

Figure S39. ^{1}H NMR spectra of the reaction of 2-methylhydroquinone with air at -20 °C, in presence of **1-Cu** (top) and **1** (bottom), spectra in CD_2Cl_2 at rt.

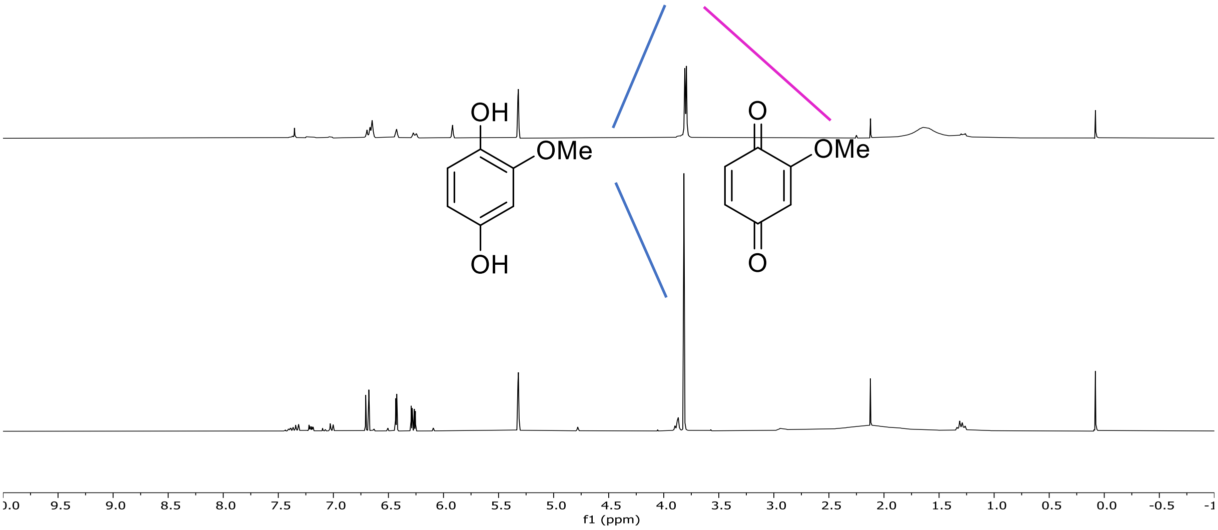

Figure S40. ^{1}H NMR spectra of the reaction of 2-methoxyhydroquinone with air at -20 °C, in presence of **1-Cu** (top) and **1** (bottom), spectra in CD_2Cl_2 at rt.

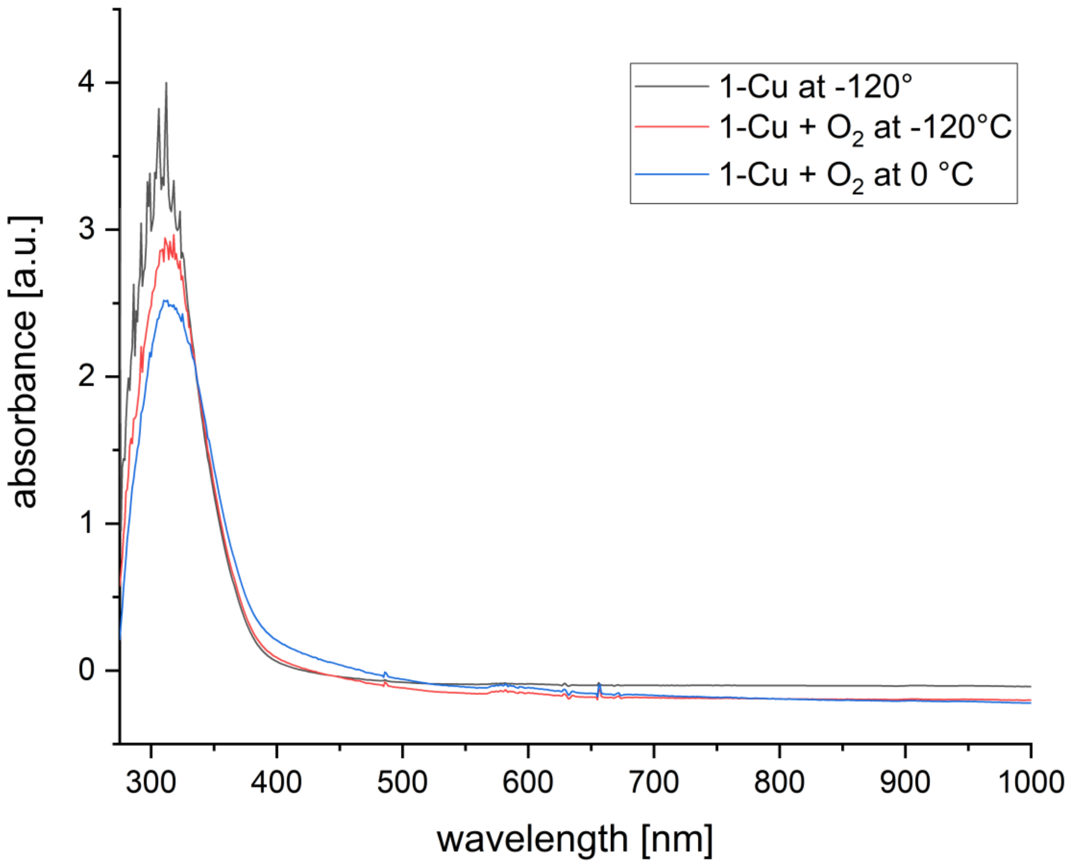

Figure S41. UV/Vis absorptions spectra of **1-Cu** at -120 °C (black), **1-Cu** + O_2 at -120 °C (red) and **1-Cu** + O_2 at 0 °C (blue). All spectra in Me-THF.

X-ray Single-Crystal Structure Analysis

CCDC-2165314 (1-Cu) and CCDC-2165315 ([1-Cu]PF6· 2(THF)) contain the supplementary crystallographic data for this paper. This data can be obtained free of charge via http://www.ccdc.cam.ac.uk/ products/csd/request/ (or from Cambridge Crystallographic Data Centre, 12 Union Road, Cambridge, CB2 1EZ, UK. Fax: +44-1223-336-033; e-mail: deposit@ccdc.cam.ac.uk)

Crystallographic Details

Suitable single crystals for X-ray structure determination were selected from the mother liquor under an inert gas atmosphere and transferred in protective perfluoro polyether oil on a microscope slide. The selected and mounted crystals were transferred to the cold gas stream on the diffractometer. The diffraction data were obtained at 100 K on a Bruker D8 three-circle diffractometer, equipped with a PHOTON III detector and an INCOATEC microfocus source with Quazar mirror optics (Mo-Kα radiation, λ= 0.71073 Å).
The data obtained were integrated with SAINT and a semi-empirical absorption correction from equivalents with SADABS was applied. The structure was solved and refined using the Bruker SHELX 2014 software package.[6] All non-hydrogen atoms were refined with anisotropic displacement parameters. All C-H hydrogen atoms were refined isotropically on calculated positions by using a riding model with their U_{iso} values constrained to 1.5 U_{eq} of their pivot atoms for terminal sp^3 carbon atoms and 1.2 times for all other atoms.

X-ray Single-Crystal Structure Analysis of 1-Cu.

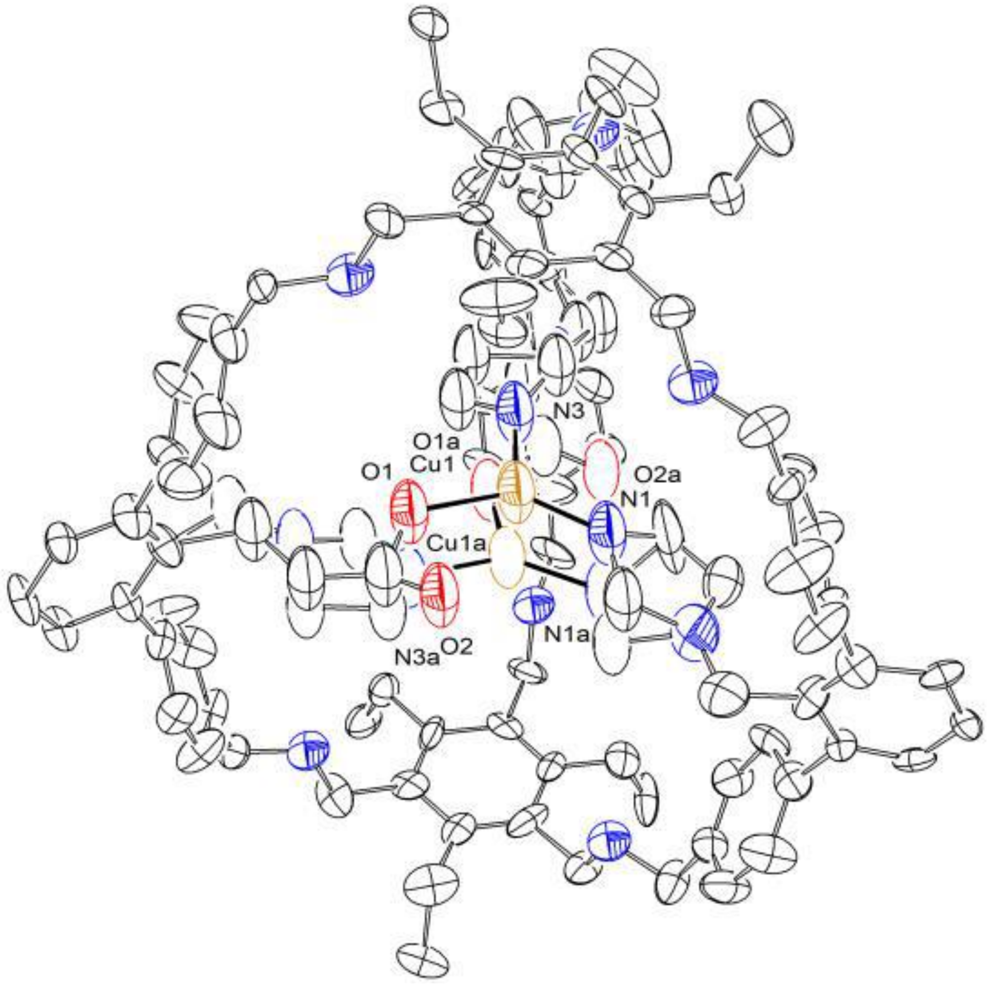

Figure S41. Thermal ellipsoid plot of **1-Cu** with the anisotropic displacement parameters drawn at the 50% probability level. The data were obtained in a resolution of 0.95 Å. The asymmetric unit contains one disordered complex molecule. The coordinating units are disordered. The disordered complex molecule was refined

with population of 0.55(9) on the main domain using restraints and constraints (RIGU, SIMU and EADP). Further DFIX, SADI and FREE commands have been applied. Further SQUEEZE routine has been applied.[7]

Table S1. Crystal data and structure refinement for **1-Cu**.

CCDC deposit number	2165314	
Empirical formula	C101 H107 Cu N10 O2	
Formula weight	1556.50	
Temperature	100(2) K	
Wavelength	0.71073 Å	
Crystal system	Monoclinic	
Space group	C 1 2 1	
Unit cell dimensions	a = 25.785(2) Å	a= 90°.
	b = 22.8050(18) Å	b= 101.118(3)°.
	c = 18.2362(14) Å	g = 90°.
Volume	10522.1(15) $Å^3$	
Z	4	
Density (calculated)	0.983 Mg/m^3	
Absorption coefficient	0.254 mm^{-1}	
F(000)	3312	
Crystal size	0.057 x 0.044 x 0.044 mm^3	
Crystal shape and color	Block,	light yellow
Theta range for data collection	2.12 to 22.08°.	
Index ranges	-27<=h<=27, -24<=k<=24, -19<=l<=19	
Reflections collected	70197	
Independent reflections	12813 [R(int) = 0.0432]	
Completeness to theta = 22.08°	99.0 %	
Absorption correction	Semi-empirical from equivalents	
Max. and min. transmission	0.99 and 0.91	
Refinement method	Full-matrix least-squares on F^2	
Data / restraints / parameters	12813 / 441 / 956	
Goodness-of-fit on F^2	1.084	
Final R indices [I>2sigma(I)]	R1 = 0.1085	wR2 = 0.2895
R indices (all data)	R1 = 0.1304	wR2 = 0.3132
Absolute structure parameter	0.508(9)	
Largest diff. peak and hole	0.707 and -0.754 e.$Å^{-3}$	

X-ray Single-Crystal Structure Analysis of [1-Cu]PF_6· 2(THF).

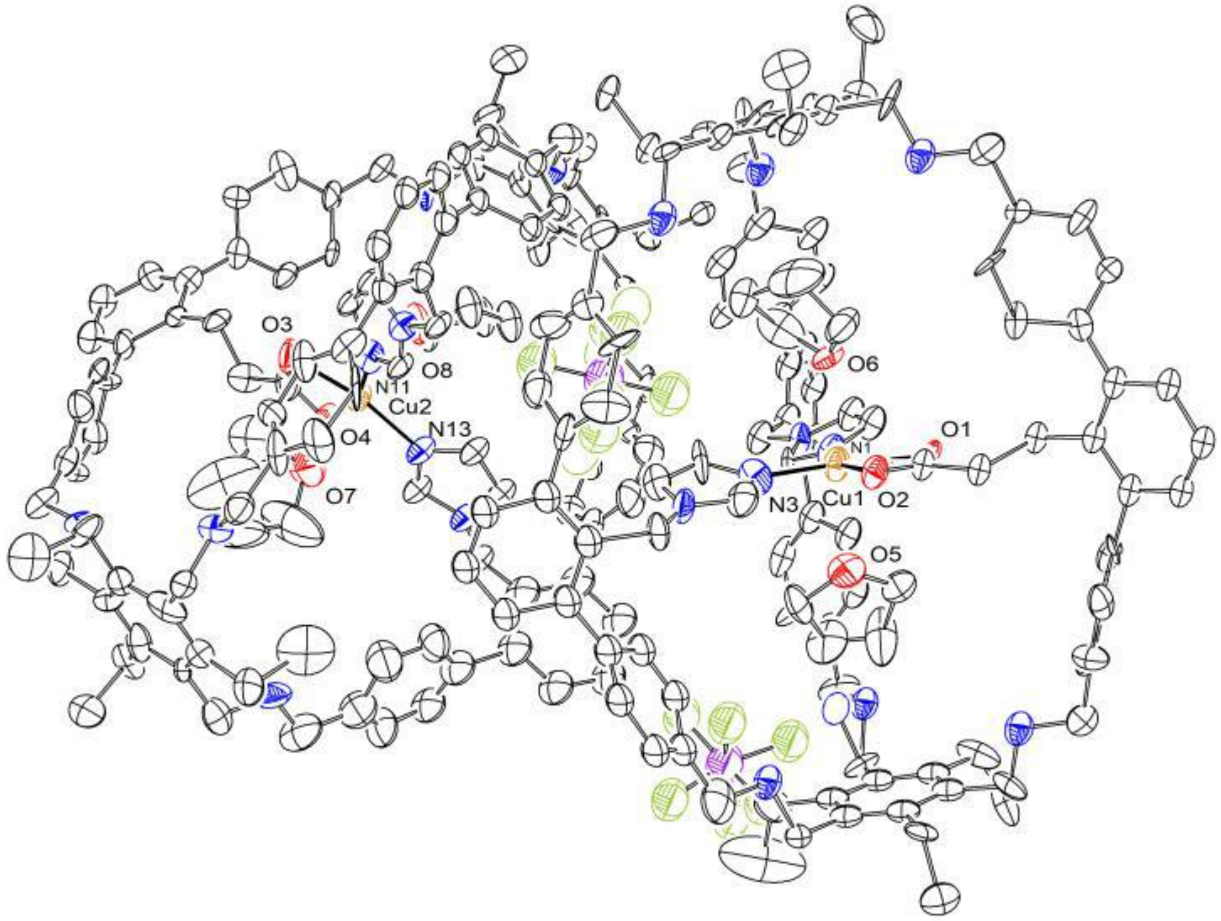

Figure S42. Thermal ellipsoid plot of [**1-Cu]PF_6· 2(THF)** with the anisotropic displacement parameters drawn at the 50% probability level. The data were obtained in a resolution of 1.04 Å. The asymmetric unit contains two complex molecules, two counter ions and four solvent molecules. One counter ion and parts of one complex molecule are disordered. The disordered counter ion was refined with a population of 0.79(7) on the main domain. The cage complex with was refined with a population of 0.61(8) on the main domain. Restraints and constraints (RIGU and EADP) were used. Further DFIX, SADI and FREE commands have been applied. Further SQUEEZE routine has been applied.[7]

Table S2. Crystal data and structure refinement for [**1-Cu]PF₆· 2(THF)**.

CCDC deposit number	2165315	
Empirical formula	$C_{218}H_{246}Cu_2F_{12}N_{20}O_8P_2$	
Formula weight	3691.35	
Temperature	100(2) K	
Wavelength	0.71073 Å	
Crystal system	Triclinic	
Space group	P-1	
Unit cell dimensions	a = 21.8689(18) Å	a = 94.580(4)°
	b = 23.944(2) Å	b = 108.970(4)°
	c = 26.510(2) Å	g = 99.957(4)°
Volume	12790.9(19) $Å^3$	
Z	2	
Density (calculated)	0.958 Mg/m^3	
Absorption coefficient	0.236 mm^{-1}	
F(000)	3908	
Crystal size	0.237 x 0.158 x 0.082 mm^3	
Crystal shape and color	Block,	light blue
Theta range for data collection	1.812 to 20.569°.	
Index ranges	-21<=h<=21, -23<=k<=23, -25<=l<=25	
Reflections collected	149317	
Independent reflections	24753 [R(int) = 0.0682]	
Completeness to theta = 20.569°	95.6 %	
Refinement method	Full-matrix least-squares on F^2	
Data / restraints / parameters	24753 / 100 / 1916	
Goodness-of-fit on F^2	3.037	
Final R indices [I>2sigma(I)]	R1 = 0.2060,	wR2 = 0.5637
R indices (all data)	R1 = 0.2377,	wR2 = 0.5932
Extinction coefficient	n/a	
Largest diff. peak and hole	3.929 and -2.722 $eÅ^{-3}$	Largest diff. peak and hole

Comparison of data obtained from X-ray Single-Crystal Structure Analysis of 1-Cu and [1-Cu]PF₆· 2(THF) with data obtained from pMMO

Table S3. Comparison of M-Ligand bond length of **1-Cu** and **1-Cu[PF6]** with Cu and Zn occupied Cu_C sites in pMMO.[8,9]

Species	M-N1 [Å]	M-N2 [Å]	M-O1 [Å]	M-O2 [Å]
**1-Cu**	1.8	2.1	2.0	2.8
**1-Cu[PF6]**	1.9	1.9	2.1	2.1
pMMO Cu[8]	2.0	2.2	2.4	2.5
pMMO Zn[9]	2.1	2.2	2.5	2.6

Values for pMMO Cu taken from PDB ID 7S4J; Values pMMO Zn taken from 3RGB.

Table S4. Comparison of Ligand-M-Ligand bond angles of **1-Cu** and **1-Cu[PF6]** with Cu and Zn occupied Cu_C sites in pMMO.[8,9]

Species	N1-M-N2 [°]	O1-M-N1 [°]	O1-M-N2 [°]	O2-M-N1 [°]	O2-M-N2 [°]
**1-Cu**	111	140	108	--	---
**1-Cu[PF6]**	105	96	159	95	159
pMMO Cu[8]	171	93	95	91	94
pMMO Zn[9]	95	80	79	111	114

Values for pMMO Cu taken from PDB ID 7S4J; Values pMMO Zn taken from 3RGB.

Computational Methods

All reported calculations were performed with the ORCA program package in its version 5.01.[10] Geometry Optimizations were performed utilizing the TPSS density functional together with the def2-SVP basis set.[11,12] To achieve an increased accuracy around the active center of the investigated compounds, the def2-TZVP basis set was assigned to the Cu atom. The RI approximation in conjunction with the def2/j auxiliary basis set was used to accelerate the formation of Fock matrices.[13,14] Dispersion effects were modeled using Grimme's D3 approximation with Becke-Johnson dampening.[15] Solvation effects were taken into account in an implicit fashion using the C-PCM,[16] employing gaussian charge distributions which yield smoother PES's.[17] Furthermore, the SMD model was employed.[18] Due to the complexity of the PES, convergence could only be reached for geometry optimizations with the aid of numerical hessians that were computed every 10-20 steps using Grimme's GFN2-XTB method in its version 6.3.3.[19]

Table S5. Comparison of experimentally determined and computed bond distances [Å] in the first coordination shell of **1-Cu**.

Bond	Experimental	Computed
Cu-O1	2.0	2.00
Cu-N1	1.8	2.00
Cu-N3	2.1	1.94

Table S6. Comparison of experimentally determined and computed bond distances [Å] in the first coordination shell of **[1-Cu]PF$_6$·2(THF)**.

Bond	Experimental	Computed
Cu-O1	2.1	2.03
Cu-O2	2.1	2.04
Cu-N1	1.9	1.96
Cu-N3	1.9	1.96
Cu-O5	2.4	2.38
Cu-O6	2.4	2.41

References

[1] T. A. Rano, G.-H. Kuo, *Org. Lett.* **2009***, 11*, 2812–2815.

[2] S. C. Bete, M. Otte, *Angew. Chem. Int. Ed.* **2021**, *60*, 18582-18586.

[3] S. Stoll, A. J. Schweiger, *Magn. Reson.* **2006***, 178*, 42-55.

[4] CRC Handbook of Chemistry and Physics. CRC Press, Cleveland, Ohio, **1977**.

[5] M. Ohashi, T. Adachi, N. Ishida, K. Kikushima, S. Ogoshi, *Angew. Chem. Int. Ed.* **2017**, *56*, 11911-11915.

[6] a) APEX3 v2016.9-0 (SAINT/SADABS/SHELXT/SHELXL), Bruker AXS Inc., Madison, WI, USA, **2016**. b) G. M. Sheldrick, *Acta Cryst.* **2015**, *A71*, 3-8. c) G. M. Sheldrick, *Acta Cryst*., **2015**, *C71*, 3-8. d) G. M. Sheldrick, *Acta Cryst.,* **2008**, *A64*, 112-122.

[7] A. L. Spek, *Acta Cryst* **2015**, *C71*, 9-18.

[8] C. W. Koo, F. J. Tucci, Y. He, A. C. Rosenzweig, *Science* **2022**, *375*, 1287-1291.

[9] S. M. Smith, S. Rawat, J. Telser, B. M. Hoffman, T. L. Stemmler, A. C. Rosenzweig, *Biochemistry* **2011**, *50*, 10231.

[10} F. Neese, F. Wennmohs, U. Becker, C. Riplinger,, *J. Chem. Phys.* **2020**, *152*, 224108.

[11] J. Tao, J. P. Perdew, V. N. Staroverov, G. E. Scuseria, *Phys. Rev. Lett.* **2003**, *91*, 146401.

[12] F. Weigend, R. Ahlrichs, *Phys. Chem. Chem. Phys.* **2005**, *7*, 3297.

[13] B. I. Dunlap, J. W. D. Connolly, J. R. Sabin, *J. Chem. Phys.* **1979**, *71*, 3396.

[14] O. Vahtras, J. Almlöf, M. W. Feyereisen, *Chem. Phys. Lett.* **1993**, *213*, 514.

[15] S. Grimme, J. Antony, S. Ehrlich, H. Krieg, *J. Chem. Phys.* **2010**, *132*, 154104.

[16] V. Barone, M. Cossi, *J. Phys. Chem. A* **1998**, *102*, 1995.

[17] M. Garcia-Ratés, F. Neese, *J. Comp. Chem.* **2020**, *41*, 922.

[18] A. V. Marenich, C. J. Cramer, D. G. Truhlar, *J. Phys. Chem. B* **2009**, *113*, 6378.

[19] C. Bannwarth, S. Ehlert, S. Grimme, *J. Chem. Theory Comput.* **2019**, 15, 1652, https://doi.org/10.1021/acs.jctc.8b01176.

Author Contributions

S.C.B. and L.K.M. carried out the synthetic work and spectroscopic characterization, supervised by M.O. M.O. carried out the crystallographic work. S.C.B. carried out the EPR simulations. P.W. and M.R. carried out the computational studies. The manuscript was written through contributions of all authors. All authors have given approval to the final version of the manuscript.
